# p38 (Mapk14/11) occupies a regulatory node governing entry into primitive endoderm differentiation during preimplantation mouse embryo development

**DOI:** 10.1098/rsob.160190

**Published:** 2016-09-07

**Authors:** Vasanth Thamodaran, Alexander W. Bruce

**Affiliations:** 1 Laboratory of Developmental Biology and Genetics (LDB&G), Department of Molecular Biology, Faculty of Science, University of South Bohemia, Branišovská 31, 37005 České Budějovice, Czech Republic; 2 Biology Centre of the Czech Academy of Sciences, Institute of Entomology, Branišovská 31, 37005 České Budějovice, Czech Republic

**Keywords:** preimplantation mouse embryo, cell-fate, primitive endoderm, mitogen-activated protein kinase, p38α/p38β Mapk14/Mapk11, cell signalling

## Abstract

During mouse preimplantation embryo development, the classically described second cell-fate decision involves the specification and segregation, in blastocyst inner cell mass (ICM), of primitive endoderm (PrE) from pluripotent epiblast (EPI). The active role of fibroblast growth factor (Fgf) signalling during PrE differentiation, particularly in the context of Erk1/2 pathway activation, is well described. However, we report that p38 family mitogen-activated protein kinases (namely p38α/Mapk14 and p38β/Mapk11; referred to as p38-Mapk14/11) also participate in PrE formation. Specifically, functional p38-Mapk14/11 are required, during early-blastocyst maturation, to assist uncommitted ICM cells, expressing both EPI and earlier PrE markers, to fully commit to PrE differentiation. Moreover, functional activation of p38-Mapk14/11 is, as reported for Erk1/2, under the control of Fgf-receptor signalling, plus active Tak1 kinase (involved in non-canonical bone morphogenetic protein (Bmp)-receptor-mediated PrE differentiation). However, we demonstrate that the critical window of p38-Mapk14/11 activation precedes the E3.75 timepoint (defined by the initiation of the classical ‘salt and pepper’ expression pattern of mutually exclusive EPI and PrE markers), whereas appropriate lineage maturation is still achievable when Erk1/2 activity (via Mek1/2 inhibition) is limited to a period after E3.75. We propose that active p38-Mapk14/11 act as enablers, and Erk1/2 as drivers, of PrE differentiation during ICM lineage specification and segregation.

## 1. Background

The appropriate formation of the murine blastocyst during the preimplantation period of development results in the derivation of three distinct cell lineages, defined as the two differentiating lineages of the trophectoderm (TE) and primitive endoderm (PrE) and the third undifferentiated and pluripotent epiblast (EPI) lineage. While the EPI population, residing deep within the inner cell mass (ICM), serves as a progenitor pool for all subsequently derived fetal cells, outer TE and PrE cells (lining the surface of the ICM facing the blastocoel) contribute to the respective placental and yolk sac extraembryonic tissues, that together form the developing conceptus *in utero* [1–3]. Exactly how extraembryonic TE and PrE initiate and maintain their differentiation, and EPI cells retain pluripotency, in a characteristically flexible and potentially regulative developmental landscape, has been the subject of many years of intense research. For example, much intensive effort has uncovered the central role of intracellular apical–basolateral polarization in regulating the differential activation of Hippo signalling, and thus appropriate cell identity, in generated outer-residing TE progenitors and inner ICM cell populations (reviewed in [4]). Similarly, key transcription factors responsible for generating blastocyst cell lineage-specific gene expression patterns have also been described (e.g. Tead4 [5,6] and Cdx2 [7] in the TE, Nanog [8] in EPI and the sequential activation of Gata6, Sox17 and Gata4 in PrE [9–14]). Additionally, intercellular signalling has emerged as an important regulatory factor, as exemplified by the results of multiple studies either inhibiting (e.g. by direct small compound mediated inhibitor blockade of fibroblast growth factor (Fgf)-receptors (Fgfr) and/or downstream extracellular signal-regulated kinase 1/2 (Erk1/2; also known as Mapk3/1) pathway activation or genetic ablation of the *Fgf4* gene) or potentiating (by exogenous addition of Fgf4 ligand) the Fgf signalling pathway leading to, respectively, impaired or increased PrE differentiation within the ICM of late blastocyst stage (E4.5) embryos [15–18]. Indeed, recent evidence also suggests a role for autocrine Fgf signalling in the derivation of functional TE [19] and, moreover, it has also been shown that bone morphogenetic protein (Bmp) signalling is important for the emergence of both the extraembryonic lineages [20]. However, a broader knowledge of how such mechanisms are integrated during mammalian preimplantation development is only just beginning to emerge.

Using *Grb^−/−^* knockout mice, Chazaud *et al.* [21] first described the necessity of the Grb2-mediated mitogen-activated protein kinase (Mapk) pathway for successful PrE formation, as evidenced by ICM cells of such blastocysts failing to establish the characteristic and mutually exclusive ‘salt and pepper’ cell expression pattern of Nanog (EPI marker) and Gata6 (an early PrE marker) (*Grb2^−/−^* knockout-derived embryos retained Nanog expression in all ICM cells, by the late-blastocyst stage [21]). It was later demonstrated, using pharmacological inhibitors for Fgfr, Mek1/2 (also known as Mkk1/2 or Map2k1/2; members of the wider mitogen-activated protein kinase kinase (Mapkk) class of kinases responsible for Erk1/2 activation) and glycogen synthase kinase 3β (Gsk3β) (together representing the so-called 3i-treatment), that establishment of the PrE programme requires activation of Mek1/2, because Mek1/2 inhibition phenocopied the *Grb2^−^*^/*−*^ knockout with all ICM cells expressing Nanog [17]. Moreover, single cell transcriptome analyses have shown that Fgfr and Fgf4 display an inverse correlative expression prior to the emergence of the salt and pepper pattern and that inhibition of Fgfr causes the downregulation of later PrE markers, *Sox17* and *Gata4*, within the ICM [22]. The dependency of Fgfr activation and the associated downstream activation of Mek1/2 (and Erk1/2) on successful PrE derivation has been further substantiated by reports showing how Fgf4 ligand from Nanog-positive EPI progenitors, themselves depleted of Fgfr, acts non-cell autonomously on PrE progenitors to promote their differentiation [15,16,18]. Indeed, recent reports [23,24] illustrate the indispensable and antagonistic tristable network relationship between Nanog, Mek1/2 and Gata6 in appropriately regulating specification and segregation of the ICM cell lineages during blastocyst maturation.

The p38 mitogen-activated protein kinases (p38-Mapk) are a class of four paralogous mammalian genes (termed p38α/Mapk14, p38β/Mapk11, p38γ/Mapk12 and p38δ/Mapk13; herein generally referred to as ‘p38-Mapks') that, together with extracellular signal-regulated kinases (e.g. Erk1/2 and Erk5) and c-Jun N-terminal kinases (Jnk), belong to the wider family of serine–threonine and tyrosine kinases regulating a wide variety of cellular functions [25]. As opposed to the Mek1/2-induced activation of Erk1/2 that results from growth factor receptor tyrosine kinase (RTK) activation, p38-Mapk isoforms are classically known to be activated by extracellular stimuli such as proinflammatory cytokines and physical stresses, mediated by specific phosphorylation events, catalysed by distinct Mapkks (e.g. Mek6/Mkk6/Map2k6 or Mek3/Mkk3/Map2k3 [26]). In turn, activated p38-Mapks are known to be able to phosphorylate (at serine and threonine residues) and activate a wide variety of downstream targets, estimated to be in the 200–300 range, that include other regulatory kinases, proteins involved in protein synthesis and turnover, transcription factors/cofactors and chromatin remodellers [27]. Moreover, in embryonic stem (ES) cell cultures, the relative activity of the p38-Mapk pathway has been found to regulate the commitment of ES cells towards either cardiac or neurogenic differentiation [28] as well as being the target of Bmp4-derived inhibition (from underlying feeder cells) that results in the promotion of self-renewal [29]. Such ES cell-derived data place p38-Mapks, and their regulation, as important and functionally relevant cell-fate mediators, acting at the interface, and governing the balance between, retention of pluripotency and the ability of cells to initiate differentiation. Indeed, just such a fundamental role may potentially apply to a broad range of *in vivo* developmental contexts, including the emergence of the three distinct preimplantation mouse embryo blastocyst cell lineages from initially totipotent cell populations. Consistently, all four p38-Mapk isoforms are known to be expressed during the preimplantation developmental period, with p38α/Mapk14 and p38δ/Mapk13 transcripts displaying robust expression levels throughout, p38β/Mapk11 having relatively lower yet steady-state levels and p38γ/Mapk12 mRNA expression steadily increasing and peaking at p38α/Mapk14 and p38δ/Mapk13 equivalent levels by the blastocyst stage [30]. Furthermore, previous work conducted using a specific small chemical compound inhibitor of p38α/Mapk14 and p38β/Mapk11 (herein referred to together as p38-Mapk14/11) has demonstrated eight- to 16-cell arrest phenotypes, associated with defects in embryo compaction, filamentous actin formation and glucose uptake, or compromised blastocyst formation typified by failures in appropriate blastocoel formation (for example, associated with tight-junction failure and reduced aquaporin expression), depending upon the exact timing of drug administration relative to the onset of embryo compaction [31–34]. A very recent study has also implicated a role for active p38-Mapk signalling in blastocyst TE formation via mediating autocrine Fgf2/Fgfr2-based signalling [19], and interesting evidence from experiments investigating the molecular regulators of canonical Wnt3a-signalling, using the mouse F9 teratocarcinoma cell model, suggests a potential role for p38-Mapks in regulating PrE formation [35]; indeed, the formation of definitive endoderm at gastrulation is known to require p38-Mapk activity [36].

Given that the majority of p38-Mapk-related work in the preimplantation mouse embryo to date has focused on developmental windows prior to the emergence of correctly specified and segregated PrE and EPI ICM populations, we decided to investigate the potential role of the p38-Mapk pathway during this latter period. Accordingly, we employed a small chemical compound inhibitor (SB220025) that specifically targets the kinase activity of p38α/Mapk14 and p38β/Mapk11, but does not affect p38γ/Mapk12 or p38δ/Mapk13 or any other member of the greater Mapk gene superfamily [37] (herein referred to as ‘p38-Mapk14/11 inhibition’), to treat embryos from the early (E3.5) to late (E4.5) blastocyst stages and assay for EPI and PrE marker protein expression. We find that after such p38-Mapk14/11 inhibition, the number of maturing or matured PrE cells, as measured using Sox17 and Gata4 protein expression, is severely diminished compared with DMSO-treated vehicle controls (further verified using a second p38-Mapk inhibiting compound, SB203580 [38], and a non-biologically active compound analogue control, SB202474 [39]). Moreover, the number of cells expressing the EPI marker Nanog is significantly increased, but this latter phenotype is also profoundly associated with co-expression of the early PrE marker, Gata6, indicating a failure of cells to commit to one of the two ICM cell-fates. We also identify the critical window of p38-Mapk14/11 activity as being early in the blastocyst maturation, manifest between the E3.5 and E3.75 developmental timepoints. Furthermore, we demonstrate that p38-Mapk14/11 functions downstream of Fgfr signalling and the non-canonically and Bmp-ligand-activated kinase, Tak1. Collectively, we interpret our findings to demonstrate a role for p38-Mapk14/11 in mediating the full entry into differentiation/maturation of PrE progenitor cells within the ICM, via as yet uncharacterized mechanisms, that are then enhanced and driven via Mek1/2-dependent pathways. Consequently, p38-Mapk14/11 occupies an important ‘PrE regulatory node’, potentially integrating multiple cell-signalling inputs that act to permit germane differentiation and segregation of PrE from EPI cell populations, during mouse embryo blastocyst ICM maturation.

## 2. Material and methods

### 2.1. Embryo culture, specific inhibitor and microinjection treatments

Two-cell stage (E1.5) embryo collection and *in vitro* culture, in KSOM supplemented with amino acids (non-essential amino acids and amino acid solution, from Gibco, diluted to working concentration of 0.5× from 100× and 50× stock solutions, respectively) was conducted as previously described [40]. Specific chemical inhibitor embryo treatments were administered in KSOM media supplemented with amino acids and targeted the stated proteins/pathways and were conducted at the follow concentrations: (i) p38-Mapk14/11; SB220025 (Calbiochem, Millipore) at 20 µM from either E2.5 to E4.5 or E3.5 to E4.5 and in experiments aimed at identifying the developmental timeframe of p38-Mapk14/11 inhibition sensitivity, during blastocyst maturation, from E3.5 to E4.0 or from E3.75/E4.0 to E4.5; we also conducted experiments from E3.5 to E4.5 using 5 µM and 10 µM concentrations (see Discussion). (ii) p38-Mapk14/11; SB203580 (plus non-biologically active analogue control SB202474, Calbiochem, Millipore) at 20 µM from E3.5 to E4.5. (iii) Mek1/2; PD0325901 (Sigma-Aldrich) at 1 µM, in the same time periods as described for p38-Mapk14/11 inhibition using SB220025, plus E2.5–E4.5. (iv) Tak1; (5Z)-7-oxozeaenol (Tocris) at 700 nM from E2.5 to E4.5. (v) Fgfr; SU5402 (Calbiochem, Millipore) at 10 µM from E3.0 to E4.5. Note that in the cases of SB220025 and PD0325901 inhibition from E3.5 to E4.0, if embryos were not to be immediately fixed, they were washed through 25 approximately 9 µl drops of normal pre-warmed KSOM growth media (to wash out the drug) and returned to *in vitro* culture and fixed at the late-blastocyst (E4.5) stage (to assay if inhibition effects were irreversible). As all inhibitors used were either supplied or reconstituted in DMSO solvent, parallel vehicle control condition embryo groups to each experimental/inhibition condition group were also established; these contained equivalent concentrations of DMSO diluted in the same KSOM media (and were therefore slightly different in relation to which experimental/inhibition group they acted as control for). Single blastomere microinjections were performed on two-cell stage embryos (in both blastomeres) according to previously described and defined protocols [41] using apparatus previously described [40]. Mutant mRNA representing constitutively active mouse-derived Mkk6 (Mkk6-EE; where serine-207 and threonine-211 were replaced with glutamate) was microinjected at a concentration of 500 ng µl^−1^ (GFP mRNA microinjection control groups were also included at the same concentration). All mRNAs were co-microinjected with Oregon green-conjugated dextran beads (OGDBs; 1 µg µl^−1^, to confirm successful microinjection). Microinjected embryos were then *in vitro* cultured and potentially exposed to defined chemical inhibitor treatments, as described above, until the desired developmental stage, in 20 µl culture drops overlaid with mineral oil at 37°C in a 5% CO_2_ containing atmosphere. Non-microinjected embryos (2–3 per experiment, per plate) served as culture sentinels to confirm successful *in vitro* development. Note that the number of individual microinjected and/or inhibitor/control-treated embryos in each specific experimental or control group is explicitly stated in the appropriate figure or electronic supplementary material figure legend and is also shown in the relevant electronic supplementary material table, which also detail information on every individual embryo analysed (the range being from 9 to 33 and an average of 18 per group).

### 2.2. Preparation of microinjected mRNA

The microinjected mRNA constructs were derived, and purified, by *in vitro* transcription of linearized (2 µg; using *SfiI*) pRN3P [42] plasmid containing the appropriate cDNA cloned downstream of the T3 bacteriophage-derived RNA polymerase promoter with 5′ and 3′ flanking UTR (untranslated region) sequences from the frog *β-globin* gene, according to the kit manufacturer's protocols (T3MessageMACHINE, Ambion). pRN3P:GFP is described elsewhere [41], but pRN3P:Mkk6-EE was newly derived for this study by PCR amplifying wild-type mouse Mkk6 cDNA insert from a brain-derived cDNA library and cloning into the multiple cloning site of pRN3P (by virtue of included *Bam*HI and *Not*I recognition sequences in the forward and reverse PCR primers, respectively), followed by PCR-based site-directed mutagenesis to derive the two required activating mutations (S207E and T211E; designated as Mkk6-EE and DNA sequence verified), using standard molecular biology techniques.

### 2.3. Immunofluorescent staining and confocal microscopy image capture

Embryos of appropriate developmental stage were prepared, fixed, immunofluorescently stained and imaged by confocal microscopy as previously detailed [40]. Primary antibodies targeting specific proteins were obtained from the following suppliers and used at the stated concentration: (i) eBioscience, Nanog (14-5761-80); 1 : 200 dilution. (ii) Abcam, Nanog (ab80892); 1 : 200 dilution. (iii) Cell Signalling Technologies, phospho-p38-Mapk14/11 (9216), phospho-Erk1/2 (9106) and cleaved caspase 3 (9661); 1 : 200 dilution. (iv) R&D Systems, Gata6 (AF1700) and Sox17 (AF1924); 1 : 200 dilution. (v) Santa Cruz, Gata4 (sc-9053) and Gata4 (sc-1237); 1 : 200 dilution. The following secondary antibodies were purchased from Life Technologies and used at 1 : 500 dilutions to detect the following epitopes: (i) Alexa647-conjugated donkey anti-rabbit (A31573); Nanog (ab80892). (ii) Alexa488-conjugated donkey anti-goat (A11055); Gata4 (sc-1237) and Sox17. (iii) Alexa647-conjugated donkey anti-rabbit (A31573); Nanog (ab80892), phospho-p38-Mapk14/11, Gata4 (sc-9053) and caspase-3. (iv) Alexa488-conjugated donkey anti-goat (A11055); Gata6. (v) Alexa488-conjugated goat anti-mouse (A11029); phospho-Erk1/2. (vi) Cy3-conjugated rat anti-mouse (A10521); Nanog (14-5761-80). Please note that owing to the non-specific interaction between microinjected OGDBs and the goat-derived anti-Gata4 antibody (sc-1237), any embryos that had been microinjected and were to be co-immunofluorescently stained for Nanog and Gata4 were done so using the following explicit primary antibody combination (hence the reason why two Nanog and Gata4 primary antibodies are listed; in non-microinjected embryos immuno-stained for Nanog and Gata4, the alternative primary antibody combination was used): mouse-derived anti-Nanog (14-5761-80) and rabbit-derived anti-Gata4 (sc-9053).

### 2.4. Image analysis/cell counting

Individual E4.5 (and E4.0) stage blastocyst cell contributions to specific cell lineages (deduced by presence and/or absence of specific immunofluorescent (IF) signal for the relevant marker protein expression), or within inner- or outer-cell populations, were determined in both experimental and control embryos by inspection of confocal micrograph z-sections using Fluoview v. 1.7.a (Olympus), Imaris (Bitplane) and ImageJ software. These contributions were individually tabulated (see electronic supplementary material, tables S1–S15) and the mean of cells within defined populations calculated and the standard error of means (mean ± s.e.m.) calculated (and presented in graphical format). The statistical significance between relevant experimental and control groups was determined using two-tailed Student's *t*-tests (see tables in the electronic supplementary material for a comprehensive review of each group and individual embryo data).

## 3. Results

### 3.1. Active p38-Mapk14/11 is required for primitive endoderm differentiation

Previous studies investigating the role of p38-Mapk14/11 during preimplantation mouse embryo development have used small compound pharmacological inhibition strategies and uncovered a requirement during the morula/blastocyst transition stage, with p38-Mapk14/11 inhibited embryos typically exhibiting arrested development, associated with failures in blastocoel formation and associated TE cell function [19,30,31]. Given that both the TE and PrE can be considered as somewhat similar extraembryonic and highly polarized epithelial blastocyst tissues, we hypothesized that p38-Mapk14/11 may also have a role in the differentiation of PrE and hence blastocyst maturation, and, moreover, that this potential role could be assayed using a similar pharmacological inhibition approach, only modified to provide the p38-Mapk14/11 inhibiting drug (SB220025) after the described sensitive morula-to-blastocyst transition. However, we first sought to confirm the results of previous studies, plus the efficacy of the used p38-Mapk14/11 inhibitor SB220025 in our laboratory, by *in vitro* culturing eight-cell stage (E2.5) embryos to the late-blastocyst (E4.5) stage in the presence of the inhibitor or DMSO vehicle control (*n.b.* we used the same concentrations as previously reported [30]). As shown in the electronic supplementary material, figure S1, we did indeed observe arrested development specifically in the SB220025-treated group; DMSO-treated embryos developed normally to form expanded/hatching blastocysts. Moreover, this phenotype was consistent with blocked development at the morula-to-blastocyst transition, as embryos typically consisted of approximately 30 cells and failed to form a blastocoel. These data hence agree with previous reports and demonstrate the efficacy of the p38-Mapk14/11 inhibition. However, we also performed IF staining, using antibodies specific for pairwise combinations of EPI and TE, or EPI and PrE lineage markers (electronic supplementary material, figure S1). These analyses showed that while the majority of outer cells in p38-Mapk14/11 inhibited embryos did express the TE marker Cdx2, indicating TE had been correctly specified, virtually all ICM cells were positive for both the EPI marker Nanog and the early PrE marker Gata6, but not the later PrE marker Sox17. This result suggested that there could indeed be a block in PrE differentiation within the ICM of p38-Mapk14/11 inhibited embryos but was tempered by the fact that the embryos were arrested, in terms of cell total number, at the morula-to-blastocyst transition. Therefore, having now confirmed the efficacy of the inhibition approach, we next sought to address the question of PrE differentiation sensitivity to p38-Mapk14/11 inhibition more directly.

**Figure 1.**
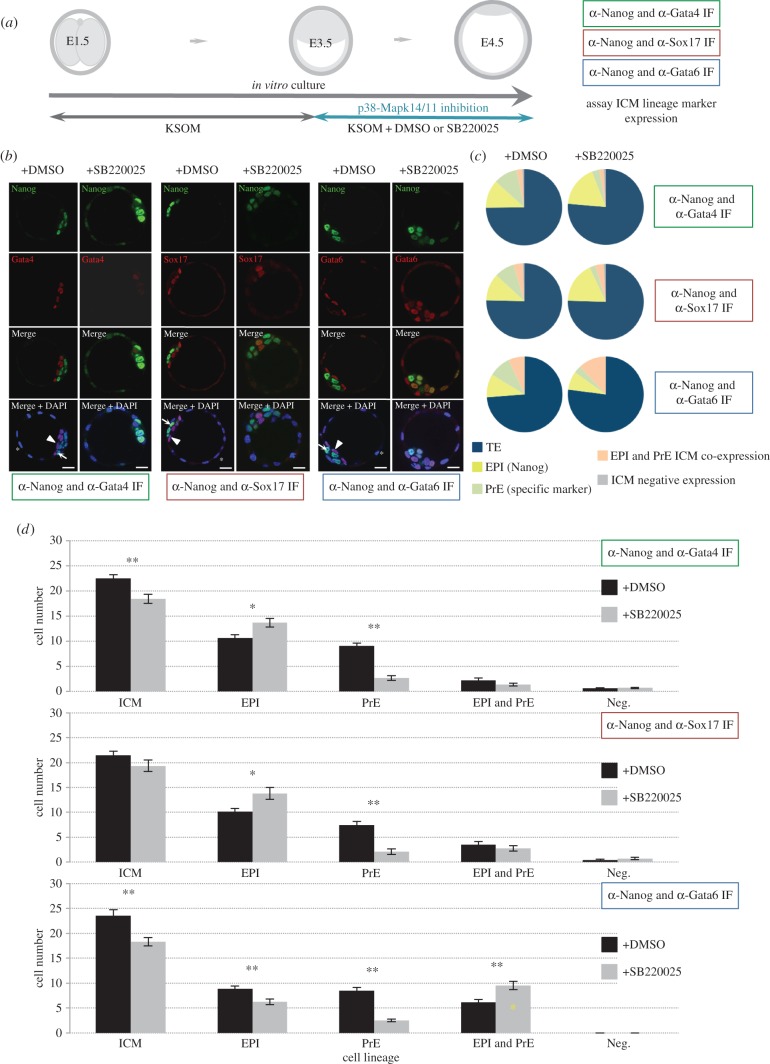
p38-Mapk14/11 inhibition during blastocyst maturation blocks PrE differentiation/maturation. (*a*) Experimental schema of p38-Mapk14/11 inhibition (+SB220025), plus vehicle control (+DMSO), and the details of antibodies used to analyse ICM cell lineage marker protein expression by immunofluorescence (IF) in late blastocysts (E4.5); Nanog and Gata4 (+DMSO *n* = 27, +SB220025 *n* = 33)—green, Nanog and Sox17 (+DMSO *n* = 18, +SB220025 *n* = 20)—red, and Nanog and Gata6 (+DMSO *n* = 24, +SB220025 *n* = 27)—blue. (*b*) Representative single confocal z-plane micrographs of vehicle control-treated (+DMSO) or p38-Mapk14/11 inhibited (+SB220025) late-blastocyst stage/equivalent embryos, immunofluorescently stained for indicated ICM cell lineage markers (Nanog in green and Gata4, Sox17 and Gata6 in red, plus DAPI DNA stain in blue). Examples of cells classified as TE, PrE and EPI are marked with an asterisk, arrowhead and arrow, respectively. Scale bar, 15 µm. (*c*) Pie charts of the relative cell lineage contribution in vehicle control (+DMSO) and p38-Mapk14/11 inhibited (+SB220025) blastocysts as judged by IF to detect the stated ICM lineage marker proteins. Blue, trophectoderm (TE); yellow, epiblast (EPI—ICM exhibiting exclusive Nanog expression); green, primitive endoderm (PrE—ICM exhibiting exclusive Gata4/6 or Sox17 expression, as appropriate); orange, uncommitted ICM cells (exhibiting co-expression of both Nanog and Gata4/6 or Sox17, as appropriate); and grey, ICM cells negative for either assayed marker. (*d*) Bar charts show average number of cells allocated to each specified ICM lineage, as judged by the indicated IF staining regime employed. Error bars represent s.e.m. and asterisks denote statistical significant differences in cell number between the vehicle control (+DMSO, black bars) and p38-Mapk14/11 inhibited (+SB220025, grey bars) embryo groups, according to two-tailed Student's *t*-test, with *p < 0.05 and **p < 0.005 confidence intervals. Yellow asterisk denotes increase in cells positively immunofluorescently staining for both EPI and PrE ICM markers using anti-Nanog and anti-Gata6 (an early PrE marker) antibodies. All individual embryo data used in the preparation of this figure are contained within the electronic supplementary material, table S3.

As referenced above, cavitated early-blastocyst stage embryos (E3.5), that had therefore successfully transited the p38-Mapk14/11 sensitive morula/blastocyst stage, were transferred into culture conditions supplemented with SB220025 or control DMSO and allowed to develop to the late-blastocyst (E4.5) stage. Embryos were then fixed and processed for IF staining for lineage-specific marker proteins, whereby Nanog was assayed in combination with either Gata6, Sox17 or the late PrE marker Gata4 (figure 1) or Cdx2 (electronic supplementary material, figure S2), to permit blastocyst cell lineage allocation to be compared. In regard to the ICM cell lineages, p38-Mapk14/11 inhibition was associated with a striking and statistically robust reduction in the number/proportion of cells expressing any of the three assayed PrE lineage markers alone (i.e. not also expressing Nanog; this effect was also observable in the ICMs of blastocysts immunofluorescently stained for Nanog and Cdx2, when one assumes Nanog-negative ICM cells belong to the PrE). Indeed, on average, the percentage contribution of such cells to the ICMs of each assayed and p38-Mapk14/11 inhibited group only represented 14.2%, 10.8% and 14.1% (when assaying for Gata6, Sox17 and Gata4, respectively) compared with an average of 36.9% in control embryo groups (when considered together across the three assayed conditions); PrE cell number reductions were associated with highly significant *p*-values (i.e. < 0.005, two-tailed Student's *t*-test; *p* = 2.21 × 10^−12^, 8.40 × 10^−7^ and 2.54 × 10^−12^, when respectively assaying Gata6, Sox17 and Gata4). We also observed a similar and significant reduction in the number of PrE cells (judged by immunofluorescently staining for Nanog and Gata4) when we employed the alternative p38-Mapk14/11 inhibitor SB203580 [30], corresponding to 10.4% of the ICM (*p* = 3.82 × 10^−9^ compared with DMSO vehicle control and *p* = 2.96 × 10^−8^ compared with non-active drug analogue SB202474-treated groups; electronic supplementary material, figure S3 and tables S5), thus independently confirming the SB220025-derived phenotype. Additionally, the readily observed cells of control embryos that solely expressed the assayed PrE markers had appropriately sorted to the blastocoel facing surface of the ICM in a manner indicative of successful PrE differentiation (figure 1), therefore confirming the suitability of our *in vitro* culture conditions. Accordingly, we interpret these data as demonstrating a significant failure in PrE differentiation/blastocyst ICM maturation that is associated with the inhibition of p38-Mapk14/11 activity. Importantly, this phenotype is not associated with altered levels of activated phospho-Erk1/2 kinase (Erk1/2(p)), extensively implicated in PrE differentiation via reports using inhibitors of its upstream activating mitogen-activated kinases, Mek1/2 [15–18], as judged by specific IF after p38-Mapk14/11 inhibition (electronic supplementary material, figure S4). However, it should be noted that the PrE differentiation inhibition phenotype was not completely penetrant, at the concentration of SB220025 used, and probably reflects heterogeneity within ICM cells to successfully integrate PrE-promoting cues. Additionally and consistent with observations made of embryos inhibited from the eight-cell (E2.5) stage, we notably recorded a statistically significant increase (*p* = 1.83 × 10^−3^) in the number/proportion of cells expressing both Nanog and Gata6, indicative of ICM cells of uncommitted fate (figure 1), manifest in an increased ICM percentage contribution of such uncommitted cells to 52% versus 26.4% in DMSO-treated controls (a similarly significant increase, *p* = 2.00 × 10^−7^, to 70% uncommitted ICM cells was also observed using the alternative SB203580 p38-Mapk14/11 inhibitor; electronic supplementary material, figure S5 and table S6). We did not observe any similar increase in co-expressing cells in p38-Mapk14/11 inhibited groups immunofluorescently stained for Nanog and the later PrE markers Sox17 or Gata4 (although we did observe enhanced levels of ectopic Nanog co-expression with Cdx2 in outer TE cells; electronic supplementary material, figure S2). Therefore, we conclude that blastocyst-stage p38-Mapk14/11 inhibition is associated with a profound cell-fate commitment failure of ICM cells. Interestingly, in the Nanog/Gata6 assayed experimental group, we observed statistically significant reductions in the number of cells expressing Nanog or Gata6 alone (*p* = 3.18 × 10^−3^ and 2.21 × 10^−12^, respectively), suggesting an inhibition to commit to both EPI and PrE cell fates. However, p38-Mapk14/11 inhibition was also associated with overall reductions in total ICM cell number (on average 3.5 fewer cells). Therefore, when the proportional effect is considered, the phenotype is stronger in regard to PrE than EPI (i.e. despite exhibiting smaller ICMs, the percentage of EPI cells within Mapk14/11 inhibited blastocyst ICMs is largely maintained, whereas the percentage of PrE cells is reduced; figure 1*c*). Indeed, when plotted as a function of overall ICM cell size (electronic supplementary material, figure S6), the number of Gata6-alone positive (i.e. PrE) cells in p38-Mapk14/11 inhibited blastocysts are clearly underrepresented compared with controls, whereas there is no difference in the representation of Nanog-alone positive (i.e. EPI) cells. Moreover, there is a consistent overrepresentation of uncommitted Nanog/Gata6 positive cells in such ICMs. Thus, these data demonstrate that while p38-Mapk14/11 inhibited blastocysts do on average present with smaller ICMs, accounting for fewer EPI cells, there is also a profound block in the commitment of cells to differentiate to PrE that is independent of this effect and reflected in the increased number/proportion of uncommitted ICM cells (as described above). The precise reason for the observed reduced ICM numbers in p38-Mapk14/11 inhibited blastocysts is not known and could arise from reduced cell proliferation and/or increased apoptosis, potentially of uncommitted cells. Indeed, IF staining for cleaved caspase 3 indicates significantly elevated levels of apoptosis in p38-Mapk14/11 inhibited embryos versus vehicle controls (electronic supplementary material, figure S7) that could account for this difference.

Therefore, in overall summary, we conclude that p38-Mapk14/11 activity is required during blastocyst ICM maturation to promote appropriate differentiation of the PrE by participating in the resolution of the uncommitted state by which cells co-express the EPI marker Nanog and the early PrE marker Gata6 (i.e. the formation of the salt and pepper pattern of mutually exclusive EPI and PrE marker expression).

### 3.2. p38-Mapk14/11 activity is required during the early stages of blastocyst maturation to permit uncommitted cells to initiate primitive endoderm differentiation

**Figure 2.**
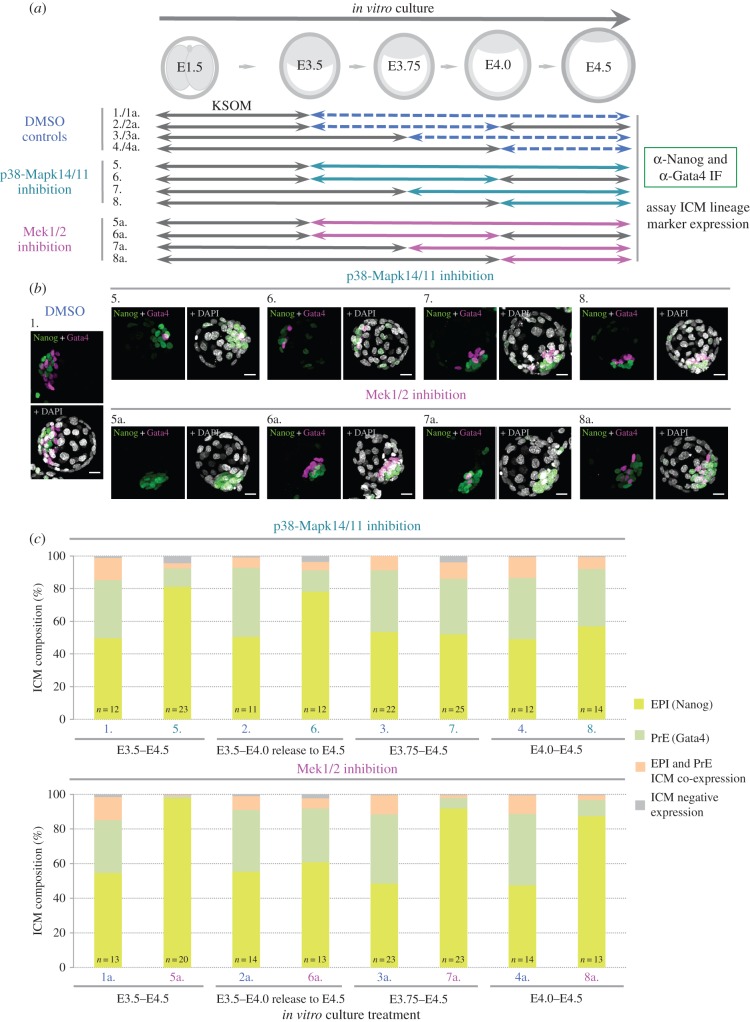
p38-Mapk14/11 is required for appropriate PrE derivation during early-blastocyst maturation and precedes Mek1/2 mediated PrE differentiation. (*a*) Experimental schema employed to identify the developmental timepoint at which p38-Mapk14/11 is required for PrE differentiation, compared with Mek1/2 activity. Two-cell (E1.5) stage embryos were *in vitro* cultured to varied blastocyst stages (ranging from E3.5 to E4.0, as indicated) and transferred into media supplemented with either p38-Mapk14/11 inhibitor (SB220025) or Mek1/2 inhibitor (PD0325901) or DMSO vehicle controls (note that the required concentrations of DMSO to control the p38-Mapk14/11 and Mek1/2 inhibition were not the same; hence the nomenclature of DMSO controls relating to Mek1/2 inhibition is suffixed here, and throughout the figure, with ‘a’). Embryos were then cultured to the late-blastocyst (E4.5) stage and fixed for IF staining against the ICM cell lineage markers Nanog and Gata4. Note that a second embryo group transferred at the early-blastocyst (E3.5) stage was removed from vehicle control/inhibitor treatment at the mid-blastocyst (E4.0) stage and returned to normal growth media before being similarly processed at the late-blastocyst (E4.5) stage. (*b*) Representative examples of immunofluorescently stained embryos as projected confocal z-stacks, from each of the above described treatment regimes (*a*); note the consistent use of nomenclature (for ease of presentation only one example of a DMSO-treated embryo is provided). Pseudo-coloured merges of detected Nanog (green) and Gata4 (magenta) protein are provided together with a further merge containing DAPI-derived DNA counterstain (pseudo-coloured white). Scale bar, 20 µm. (*c*) Percentage bar charts detailing the averaged relative cell lineage composition of ICMs from embryos derived from each of the above described treatment regimes (*a*); note the consistent use of nomenclature (and the change in the order of the treatments—for ease of interpretation DMSO vehicle control embryos are labelled in blue, p38-Mapk14/11 inhibited embryos in sea green and Mek1/2 inhibited embryos in magenta). The averaged percentage contribution of ICM cells to each lineage, within the chart, is highlighted: EPI (yellow), PrE (green), EPI and PrE ICM co-expression (orange) and ICM negative expression (grey). The number of embryos analysed in each group is highlighted within each percentage bar. Additionally, the same data are presented as the average number of cells contributing to each blastocyst lineage in the electronic supplementary material, figures S8 and S9. All individual embryo data used in the preparation of this figure are contained within the electronic supplementary material, tables S7 and S8.

As described above, the role of active Mek1/2 during PrE differentiation in the ICM has been well characterized. Indeed, pharmacological experiments investigating the developmental timing of Mek1/2 requirement, albeit in combination with concomitant Fgfr inhibition, demonstrate a developmental plasticity of ICM cells up to a point between E4.0 and E4.5, when cell-fate becomes irreversibly committed (i.e. the inhibitor treatment is no longer able to convert, nearly all, ICM cells to an EPI fate) [18]. Accordingly, we wanted to similarly assay the temporal requirement for p38-Mapk14/11 activity, during PrE formation, and to directly compare it with that for active Mek1/2 (assayed by targeting Mek1/2 alone, i.e. not in combination with Fgfr inhibition). We therefore, conducted a series of experiments in which embryos were *in vitro* cultured to the late-blastocyst/E4.5 stage in the presence of either p38-Mapk14/11 (SB220025) or Mek1/2 (PD0325901) inhibitor, from either the early-blastocyst/E3.5 (as previously assayed for p38-Mapk14/11 inhibition—see figure 1 and electronic supplementary material, figure S2), E3.75 or mid-blastocyst/E4.0 stages and assayed for EPI (Nanog) and late-PrE (Gata4) lineage marker expression within the ICM (*n.b.* Gata4 marker was selected to ensure we assayed for specified PrE). An additional condition in which embryos were cultured in the presence of inhibitor from E3.5 to E4.0 and then returned into normal growth media for continued culture until the assayed late-blastocyst (E4.5) stage was also included (i.e. an experimental condition assaying the potential reversibility of inhibitor-induced phenotypes), as were control conditions for all inhibitor permutations in which embryos were exposed to appropriate concentrations of vehicle control DMSO (figure 2 and electronic supplementary material, figures S8 and S9 and tables S7 and S8). Consistent with our previous experiments (figure 1), we found that p38-Mapk14/11 inhibition from E3.5 to E4.5 was again associated with a profound, although not entirely penetrant, block in PrE formation (also associated with an increased number/proportion of Nanog-alone positive ‘EPI-like’ cells, represented by an average 11.2% PrE and 81.1% EPI-like make-up, the remaining ICM cells either co-expressing or negative for the assayed proteins). The identical treatment regime targeting Mek1/2 resulted in near 100% conversion of all ICM cells to Nanog-alone expression status, with virtually no evidence of Gata4 expression (on average yielding 1.0% PrE and 97.4% EPI-like ICM cell contribution), in marked similarity to previously reported data inhibiting Mek1/2 (plus Fgfr) from E2.5 to E4.5 [18]. While both treatments highlight the requirement for both p38-Mapk14/11 and Mek1/2 activity during the appropriate derivation of PrE, the latter results suggest that Mek1/2 activity has a more profound effect than p38-Mapk14/11, at least at the inhibitor concentrations used. Moreover, given that both p38-Mapk14/11 inhibition does not alter detectable levels of activated Erk1/2(p) (electronic supplementary material, figure S4) and Mek1/2 inhibition does not affect activated phosphorylated-p38-Mapk14/11 (p38-Mapk14/11(p)) levels (electronic supplementary material, figure S10), thereby excluding cross-reactivity effects of the chemical inhibitors used, the data suggest that even in the presence of normal levels of activated p38-Mapk14/11, inhibition of Mek1/2 is sufficient to block PrE differentiation, whereas even if the p38-Mapk14/11 pathway is inhibited some PrE differentiation, potentially driven by active Mek1/2, is able to occur. PD0325901-treated, Mek1/2-inhibited embryos remained sensitive to Mek1/2 inhibition administered from E3.75 or E4.0, leading to appreciable and statistically significantly increased levels of EPI conversion, compared with vehicle control (*p* = 7.00 × 10^−7^ and 1.91 × 10^−8^, respectively), as represented by the respective averaged PrE and EPI percentage contributions of 6.1% and 91.9% in embryos treated from E3.75 and 9.1% and 87.4% in groups treated from E4.0 (*n.b.* some Gata4-alone positive and lineage marker co-expressing ICM cells did appear, in these latter two treatment conditions, suggesting some cells had already committed, or begun commitment, to PrE fate before the inhibitor was provided). However, the administration of p38-Mapk14/11 inhibitor (SB220025) after E3.75 had no significant effect on ICM cell lineage specification/segregation, when compared with appropriate DMSO-treated control embryos; for example, the PrE and EPI percentage contributions in embryo groups treated from E3.75 to E4.5 were 37.6% and 53.7% for DMSO controls and 38% and 52.1% for p38-Mapk14/11 inhibited embryos. These results suggested that the developmental window at which p38-Mapk14/11 activity is required for appropriate PrE differentiation is before E3.75 (i.e. in the context of the experiment, it is between E3.5 and E3.75, but as it is not possible to study the effect of p38-Mapk14/11 inhibition on PrE differentiation by providing the inhibitor before E3.5, owing to the induced morula-to-blastocyst transitional arrest (see electronic supplementary material figure S1) it could start before E3.5), whereas the Mek1/2 inhibition-sensitive window extends beyond E4.0, again in agreement with previous studies [18]. This conclusion was confirmed when embryos that had been exposed to inhibitor treatment from E3.5 to E4.0 and then transferred back to normal growth media were assayed. In such embryos exposed to a pulse of p38-Mapk14/11 inhibition, the classically observed PrE deficit (i.e. as achieved when inhibiting continuously from E3.5 to E4.5) was still robustly and statistically significantly evident; the averaged percentage ICM contribution of PrE cells was 12.6% versus 42.2% in similarly treated DMSO control groups (*p* = 1.10 × 10^−8^). However, in embryos treated with a pulse of Mek1/2 inhibition, the number of Nanog-alone (EPI) and Gata4-alone (PrE) cells (plus co-expressing cells and cells negative for expression of either marker) were statistically equivalent to DMSO vehicle control-treated embryos, indicative of the reversible and plastic nature of Mek1/2-driven PrE differentiation, not shared by p38-Mapk14/11. Therefore, we interpret these data as demonstrating a requirement for active p38-Mapk14/11-mediated signalling during the earliest stages of blastocyst ICM maturation that is needed to appropriately derive the PrE and EPI cell lineages.

**Figure 3.**
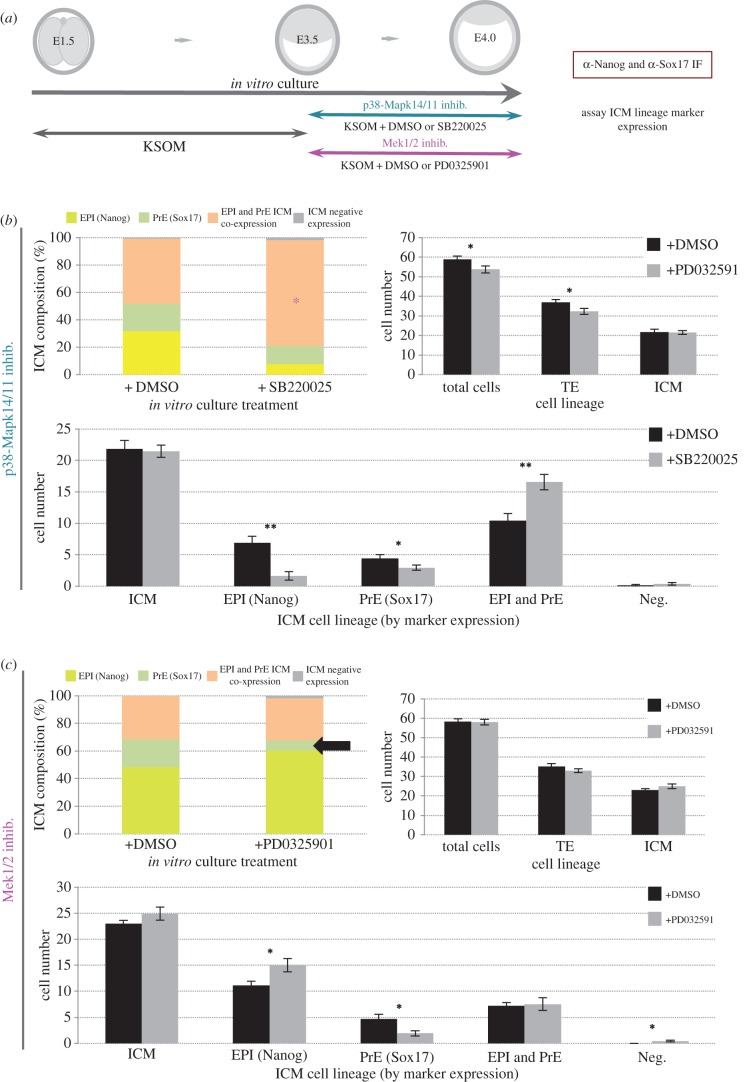
p38-Mapk14/11 inhibition during early-blastocyst maturation produces ICM cells of uncommitted fate, whereas Mek1/2 inhibition prevents differentiation to the PrE lineage. (*a*) Experimental schema employed to study the effect on ICM lineage separation of p38-Mapk14/11 and Mek1/2 inhibition during early-blastocyst maturation. Cultured embryos were transferred to growth media supplemented with vehicle control (+DMSO; note two concentrations used depending on the specific inhibitor to be used—see below) or inhibitor against p38-Mapk14/11 (+SB220025) or Mek1/2 (PD0325901) and permitted to develop to the mid-blastocyst (E4.0) stage, fixed and immunofluorescently stained for EPI (Nanog) and PrE (Sox17) marker proteins. (*b*) Relating to p38-Mapk14/11 inhibition (*n* = 13 and 13 for control and inhibitor treated groups, respectively), and (*c*) relating to Mek1/2 inhibition experiments (*n* = 12 and 13 for control and inhibitor treated groups, respectively); percentage bar charts show the relative contribution of ICM cells to respective lineages (EPI in yellow, PrE in green, uncommitted cells expressing both EPI and PrE markers in orange and cells not expressing either marker in grey). Note, decreased maturation of EPI and PrE in p38-Mapk14/11 inhibited embryos is due to increased proportion of uncommitted cells (highlighted by magenta asterisk), whereas in Mek1/2 inhibited embryos the proportion of uncommitted cells is no different to controls but the contribution of solely *Sox17* expressing PrE cells is diminished (highlighted by black arrow) and solely *Nanog* expressing EPI cells is increased; the average number of cells in each treatment and accompanying control regime, contributing to total embryo cell number, outer TE and inner ICM lineages are shown as bar charts, as is the average number of cells in each ICM lineage. Errors are representative of s.e.m. and asterisks denote statistical significant differences in cell number between the vehicle control (black bars) and p38-Mapk14/11 or Mek1/2 inhibited (grey bars) embryo groups, according to two-tailed Student's *t*-test, with *p < 0.05 and **p < 0.005 confidence intervals. All individual embryo data used in the preparation of this figure are contained within the electronic supplementary material, tables S9 and S10.

Given that we had identified germane differentiation of the PrE is not sensitive to p38-Mapk14/11 inhibition subsequent to the E3.75 stage (figure 2), the stage at which the salt and pepper pattern of EPI and PrE marker expression begins to resolve [13,21], and that the ICMs of blastocysts *in vitro* cultured in the presence of p38-Mapk14/11 inhibitor from E3.5 to E4.5 display significantly increased numbers/proportions of Nanog positive and Gata6 positive uncommitted co-expressing cells (figure 1 and electronic supplementary material figure S5), we hypothesized that p38-Mapk14/11 inhibition during the sensitive window would also result in significantly increased levels of uncommitted ICM cells. We therefore treated early blastocysts from E3.5 until E4.0 with either p38-Mapk14/11 or Mek1/2 inhibitor and then fixed and immunofluorescently stained for Nanog and Sox17 (figure 3 and electronic supplementary material, tables S9 and S10). We found that DMSO vehicle control-treated embryo ICMs contained a substantial number/proportion of Nanog and Sox17 co-expressing uncommitted cells (representing an average of 39.7% of ICM cells, between the two control groups), appropriate to the temporal midpoint of blastocyst maturation (*n.b.* in relation to p38-Mapk14/11 and Mek1/2-treated blastocyst groups, different concentrations of DMSO vehicle control were administered). However, the number/proportion of similarly uncommitted cells observed in the p38-Mapk14/11 group was significantly increased (to 76.7% of all ICM cells, *p* = 1.23 × 10^−3^), mostly at the expense of specified EPI (i.e. Nanog-alone expressing), but also specified PrE (i.e. Sox17-alone expressing) cells, whereas there were no uncommitted cell differences in Mek1/2 inhibited embryo ICMs, despite the fact the number/proportion of EPI and PrE specified cells was, respectively, increased (to an ICM average contribution of 60.2% from 48.3%) and decreased (to 7.6% from 20.4%). We therefore interpret these data as supporting a relatively early role for p38-Mapk14/11 in facilitating the resolution of uncommitted cells and a role for Mek1/2 in driving PrE differentiation (without itself affecting the fate resolution of uncommitted ICM cells), that is subsequent to, or concomitant with, the emergence of the salt and pepper expression pattern of ICM lineage markers.

### 3.3. Fgf-receptor-mediated cell signalling and the activation of the non-canonically activated Bmp-related kinase Tak1 functionally activates p38-Mapk14/11 during PrE derivation

**Figure 4.**
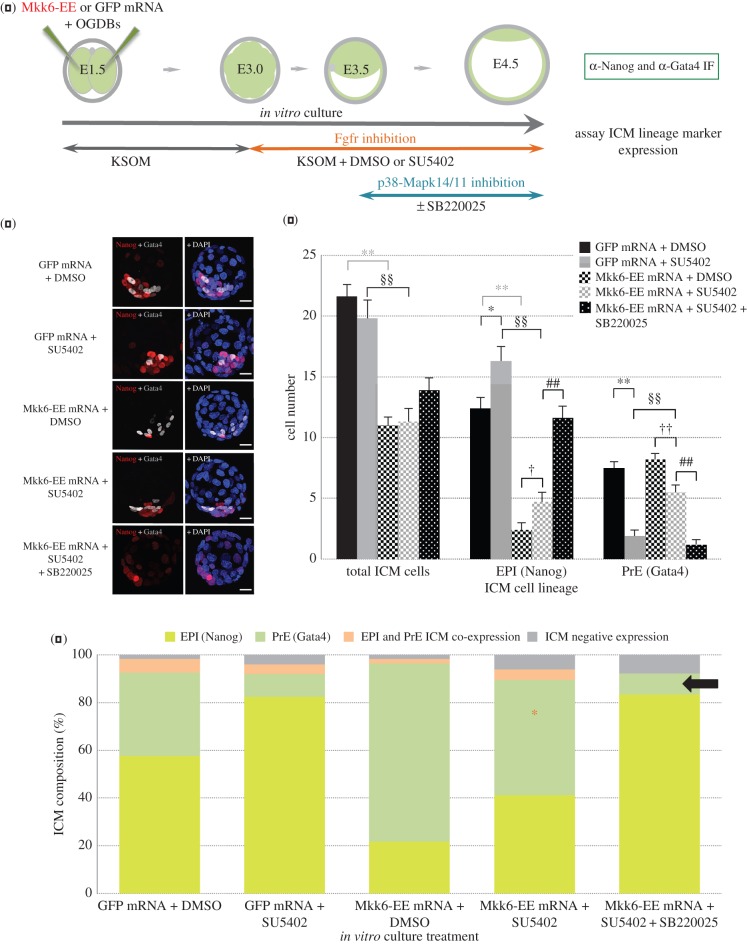
Inhibition of Fgf-receptors (Fgfr) inhibits PrE formation in a p38-Mapk14/11 dependent manner. (*a*) Experimental schema detailing the regime of Fgfr inhibition (+SU5402), with attendant vehicle control (+DMSO) condition, from the 16-cell to late-blastocyst (E3.0-E4.5) stages and optional p38-Mapk14/11 co-inhibition (±SB220025, from E3.5 to E4.5), employed. Also highlighted are mRNAs microinjected (together with Oregon-green conjugated dextran beads (OGDBs), to confirm successful mRNA delivery) into both blastomeres at the two-cell (E1.5) stage: the constitutively active, p38-Mapk14/11 targeting kinase, ‘Mkk6-EE’ mutant or microinjection control ‘GFP’. Immunofluorescence (IF) antibody details used to analyse ICM cell lineage marker protein expression in late blastocysts (E4.5) are also given. (*b*) Representative confocal z-plane projections of ICM lineage marker expression (Nanog, for EPI, in red and Gata4, for PrE, in grey-scale, plus DAPI DNA counter-stain in blue) in each of the studied conditions in late-blastocyst (E4.5) stage embryos: GFP microinjection control plus DMSO vehicle control (GFP mRNA + DMSO; *n* = 23), GFP microinjection control plus Fgfr inhibition (GFP mRNA + SU5402; *n* = 23), Mkk6-EE microinjection plus DMSO vehicle control (Mkk6-EE mRNA + DMSO; *n* = 24), Mkk6-EE microinjection plus Fgfr inhibition (Mkk6-EE mRNA + SU5402; *n* = 23) and Mkk6-EE microinjection plus Fgfr and p38-Mapk14/11 inhibition (Mkk6-EE mRNA + SU5402 + SB220025; *n* = 22). Scale bar, 15 µm. (*c*) Averaged contribution of cells to each ICM cell lineage, based on exclusive expression of either EPI (Nanog) or PrE (Gata4) lineage marker, in each of the stated experimental conditions. Errors are represented as s.e.m. and appropriate statistically significant differences, derived from two-tailed Student's *t*-tests, highlighted by one or two significance markers (one representing *p* < 0.05 and two denoting *p* < 0.005) described thus; asterisks (*) show differences between the ‘GFP mRNA + DMSO’ and ‘GFP mRNA + SU5402’ or ‘Mkk6-EE mRNA + DMSO’ (in grey) groups, the section symbol (§) highlights the significant difference between the ‘GFP mRNA + SU5402'and ‘Mkk6-EE + SU5402’ groups, crosses (†) between the ‘Mkk6-EE + DMSO’ and ‘Mkk6-EE + SU5402’ groups, and hashtags (#) denote divergence between the ‘Mkk6-EE + SU5402’ and ‘Mkk6-EE + SU5402 + SB220025’ groups. Data are also presented in the electronic supplementary material, table S11 (note, extra data relating to total cell and TE cell numbers are similarly described in the electronic supplementary material, figure S12). (*d*) Averaged percentage make-up of the ICMs of each stated condition in relation to each specified ICM lineage: EPI or PrE (yellow and green, exclusively immunostained for either Nanog or Gata4, respectively), EPI and PrE co-expressing cells (orange, representing cells uncommitted to either lineage) and cells negative for either studied lineage marker (grey). Orange asterisk denotes the rescue of the PrE component of ICM cells in Fgfr inhibited embryos expressing the p38-Mapk14/11 activating kinase mutant, Mkk6-EE (‘Mkk6-EE mRNA + SU5402’ group) compared with the appropriate Fgfr inhibited condition (the ‘GFP mRNA + SU5402’ group). Similarly, the black arrow highlights the ablation of this rescue effect by additional p38-Mapk14/11 inhibition (in the ‘Mkk6-EE mRNA + SU5402 + SB220025’ group).

As previously discussed, the role of Fgf-ligands in the specification of the PrE cell lineage is well described [15–18]. We therefore wanted to investigate if Fgfr-mediated cell signalling during specification of the PrE was acting, in part, via the activation of p38-Mapk14/11, as has recently been shown during TE specification [19]. Accordingly, we used a well-characterized and specific chemical inhibitor, SU5402 [43], that has been widely used in preimplantation mouse embryo studies [17,22] to functionally block Fgfr, and assayed for p38-Mapk14/11-dependent PrE phenotypes. Treatment of *in vitro* cultured embryos from the eight-cell (E2.5) to the late-morula (E3.25) stage with SU5402 revealed reduced levels of activated p38-Mapk14/11(p), as detected by confocal microscopy-based IF staining (see electronic supplementary material, figure S10), confirming p38-Mapk14/11 activation is susceptible to Fgfr-mediated cell signalling levels in the preimplantation mouse embryo. We next sought to confirm, using identical inhibitor regimes to those previously described for SU5402 [22], that Fgfr inhibition results in defective PrE formation and to assay if any component of this phenotype was dependent on active p38-Mapk14/11. Therefore, isolated two-cell (E1.5) stage embryos were microinjected, in both blastomeres, with recombinant control GFP mRNA or transcript for a constitutively active form of the p38-Mapk14/11 targeting and activating kinase Mkk6, also known as Map2k6 (*n.b.* Oregon-green conjugated dextran beads (OGDBs) were also microinjected to confirm construct delivery). The mutant, mouse sequence-derived, Mkk6 kinase contains two phosphomimetic amino acid substitutions, S207E and T211E (designated Mkk6-EE), and when expressed in the preimplantation mouse embryo results in increased activated p38-Mapk14/11(p) levels (electronic supplementary material, figure S10) without affecting activated Erk1/2(p) levels (electronic supplementary material, figure S11); moreover, extensive structural and biochemical studies have confirmed that Mkk6 specifically targets all p38-Mapks (and preferentially targets p38-Mapk14/11) and does not affect extracellular signal-regulated kinases (e.g. Erk1/2 and Erk5) or c-Jun N-terminal kinase (Jnk) substrates [44,45]. Microinjected embryos, from each group, were then cultured until the 16-cell (E3.0) stage and transferred into media containing either Fgfr inhibitor (+SU5402) or vehicle control (+DMSO) and further cultured until the late-blastocyst (E4.5) stage. An additional group of Mkk6-EE microinjected and SU5402-treated embryos was subject to an extra experimental step, whereby the Fgfr inhibitor containing media was further supplemented with p38-Mapk14/11 inhibitor (+SB220025) at the early-blastocyst (E3.5) stage, before continued culture to the late-blastocyst (E4.5) stage. ICM cell lineage derivation was then determined in each experimental and control group by confocal microscopy-based IF staining for Nanog (as an EPI marker) and Gata4 (as a late PrE marker). As can be seen (figure 4 and electronic supplementary material, figure S12 and tables S11), the treatment of GFP microinjected control embryos with Fgfr inhibitor caused a statistically significant decrease and increase in the number/proportion of derived PrE (from an average ICM percentage contribution of 34.7% in DMSO-treated controls to 9.6%, *p* = 8.08 × 10^−10^) and EPI (from 57.4% to 82.3%, *p* = 1.18 × 10^−2^), respectively, without significantly altering overall ICM cell number; this phenotype is in agreement with previous studies [17,22]. Strikingly, this Fgfr inhibitor-mediated PrE deficit phenotype could be largely rescued, in terms of total PrE cell number and the proportion of PrE specified ICM cells (increasing to an ICM contribution of 48.8%, *p* = 5.28 × 10^−5^), by the overexpression of the Mkk6-EE construct, albeit Mkk6-EE microinjected embryos presented with fewer total and ICM cells (compared with GFP microinjected controls). This result suggests that p38-Mapk14/11 is functionally activated downstream of Fgfr-mediated PrE specification; indeed expression of the Mkk6-EE construct in DMSO vehicle control embryos alone was enough to result in 74.5% of derived ICM cells to adopt a PrE fate. This interpretation is confirmed by the fact that when Fgfr-inhibited and Mkk6-EE microinjected embryos were subject to additional p38-Mapk14/11 inhibition, the PrE rescue effect of Mkk6-EE expression was completely ablated, with both the proportional PrE make-up of the ICM and the total PrE cell number returning to statistically equal levels observed in control GFP mRNA microinjected embryos treated with Fgfr inhibitor; this phenotype was also accompanied by a similar increase in EPI (Nanog-alone positive) cells. Collectively, we interpret these data as demonstrating a functional role for p38-Mapk14/11 that is downstream of PrE-promoting cell signalling mediated by activated Fgfr in the maturing ICM of preimplantation mouse blastocysts.

**Figure 5.**
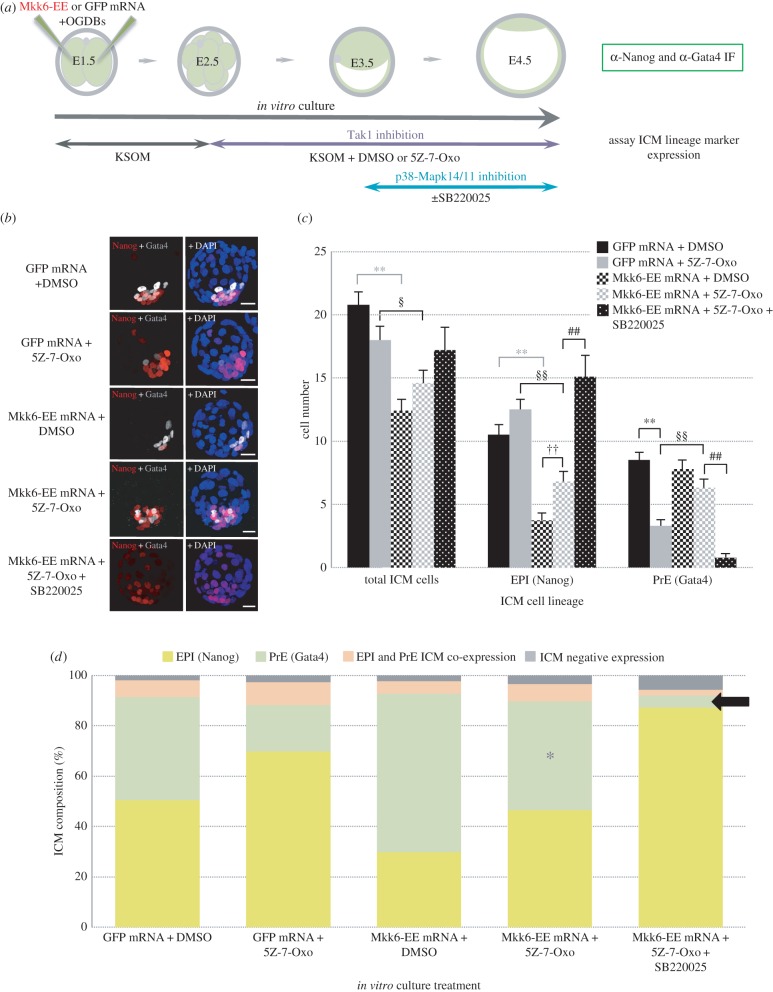
Inhibition of Tak1 inhibits PrE formation in a p38-Mapk14/11 dependent manner. (*a*) Experimental schema detailing the regime of Tak1 inhibition (+5Z-7-Oxo), with attendant vehicle control (+DMSO) condition, from the eight-cell to late-blastocyst (E2.5–E4.5) stages and optional p38-Mapk14/11 co-inhibition (±SB220025, from E3.5 to E4.5), employed. Also highlighted are mRNAs microinjected (together with Oregon-green conjugated dextran beads (OGDBs), to confirm successful mRNA delivery) into both blastomeres at the two-cell (E1.5) stage: the constitutively active, p38-Mapk14/11 targeting kinase, ‘Mkk6-EE’ mutant or microinjection control ‘GFP’. Immunofluorescence (IF) antibody details used to analyse ICM cell lineage marker protein expression in late blastocysts (E4.5) are also given. (*b*) Representative confocal z-plane projections of ICM lineage marker expression (Nanog, for EPI, in red and Gata4, for PrE, in grey scale, plus DAPI DNA counterstain in blue) in each of the studied conditions in late-blastocyst (E4.5) stage embryos: GFP microinjection control plus DMSO vehicle control (GFP mRNA + DMSO; *n* = 22), GFP microinjection control plus Tak1 inhibition (GFP mRNA + 5Z-7-Oxo; *n* = 23), Mkk6-EE microinjection plus DMSO vehicle control (Mkk6-EE mRNA + DMSO; *n* = 25), Mkk6-EE microinjection plus Tak1 inhibition (Mkk6-EE mRNA + 5Z-7-Oxo; *n* = 25) and Mkk6-EE microinjection plus Tak1 and p38-Mapk14/11 inhibition (Mkk6-EE mRNA + 5Z-7-Oxo + SB220025; *n* = 21). Scale bar, 15 µm. (*c*) Averaged contribution of cells to each ICM cell lineage, based on exclusive expression of either EPI (Nanog) or PrE (Gata4) lineage marker, in each of the stated experimental conditions. Errors are represented as s.e.m. and appropriate statistically significant differences, derived from two-tailed Student's *t*-tests, highlighted by one or two significance markers (one representing *p* < 0.05 and two denoting *p* < 0.005) described thus; asterisks (*) show differences between the ‘GFP mRNA + DMSO’ and ‘GFP mRNA + 5Z-7-Oxo'or ‘Mkk6-EE mRNA + DMSO’ (in grey) groups, the section symbol (§) highlights the significant difference between the ‘GFP mRNA + 5Z-7-Oxo'and ‘Mkk6-EE + 5Z-7-Oxo’ groups, crosses (†) between the ‘Mkk6-EE + DMSO’ and ‘Mkk6-EE + 5Z-7-Oxo’ groups, and hashtags (#) denoting divergence between the ‘Mkk6-EE + 5Z-7-Oxo’ and ‘Mkk6-EE + 5Z-7-Oxo + SB220025’ groups. Data are also presented in the electronic supplementary material, table S12 (note, extra data relating to total cell and TE cell numbers are similarly described in the electronic supplementary material, figure S13). (*d*) Averaged percentage make-up of the ICMs of each stated condition in relation to each specified ICM lineage: EPI or PrE (yellow and green, exclusively immunostained for either Nanog or Gata4, respectively), EPI and PrE co-expressing cells (orange, representing embryos uncommitted to either lineage) and cells negative for either studied lineage marker (grey). Purple asterisk denotes the rescue of the PrE component of ICM cells in Tak1 inhibited embryos expressing the p38-Mapk14/11 activating kinase mutant, Mkk6-EE (‘Mkk6-EE mRNA + 5Z-7-Oxo’ group) compared with the appropriate Tak1 inhibited condition (the ‘GFP mRNA + 5Z-7-Oxo’ group). Similarly, the black arrow highlights the ablation of this rescue effect by additional p38-Mapk14/11 inhibition (in the ‘Mkk6-EE mRNA + 5Z-7-Oxo + SB220025’ group).

A relatively recent study identified a role for secreted Bmp-ligands and activated Bmp-receptors (Bmpr), in the derivation the PrE (and TE) during mouse blastocyst ICM cell lineage specification and segregation. In addition to classical Smad-dependent mechanisms, this study also uncovered a non-canonical/Smad-independent mechanism of PrE derivation that functionally requires the active kinase, Tak1 (also known as Mkk7/Map3k7) [20]. As Tak1 kinase is able to target and activate both Mkk6 and Mkk3, that themselves are known to be the only targeting and activating enzymes of p38-Mapks [46,47], we wanted to test if any of the reported Tak1-mediated inhibitory effects on PrE derivation [20] were mediated by p38-Mapk14/11. As with embryos cultured in the presence of Fgfr inhibitor, we found incubation of eight-cell (E2.5) stage embryos *in vitro* cultured to the late-morula (E3.25) stage in the specific Tak1 inhibitor (5*Z*)-7-oxozeanol [48] (abbreviated herein to 5*Z*-7-Oxo), resulted in reduced levels of activated p38-Mapk14/11(p) protein (electronic supplementary material, figure S10), demonstrating p38-Mapk14/11 activation is susceptible to functional Tak1 levels in the preimplantation mouse embryo. Therefore, we next decided to conduct a Tak1/Mkk6-EE/p38-Mapk14/11 inhibition experiment, conceptually similar to that described above for Fgfr inhibition but with the difference that the 5*Z*-7-Oxo Tak1 inhibitor was provided from the eight-cell (E2.5) stage rather than from the 16-cell (E3.0) stage, as has previously been reported for uncovering PrE deficit phenotypes [20] (figure 5 and electronic supplementary material, figure S13 and table S12). As can be observed, we obtained extremely similar results to the Fgfr inhibition experiment (figure 4), in that Tak1 inhibition in GFP microinjected control embryos caused a significant reduction in overall PrE cell number and the proportional make-up of the ICM (down to 18.3% from the 40.9% observed in DMSO-treated control embryos, *p* = 5.53 × 10^−8^). This PrE deficit phenotype was concomitant with an increase in the number/proportion of ICM cells solely expressing the EPI marker Nanog (up to 69.5% versus 50.5% in controls). Moreover, the PrE phenotype caused by Tak1 inhibition was rescued by over expression of the p38-Mapk14/11 activating Mkk6-EE construct, but the rescue could also be ablated by subsequent direct inhibition of p38-Mapk14/11 itself, thereby confirming that Tak1 activity, itself functionally downstream of activated Bmpr [20], functions to promote PrE derivation in the mouse blastocyst ICMs by contributing to the activation of p38-Mapk14/11.

### 3.4. Concluding summary

**Figure 6.**
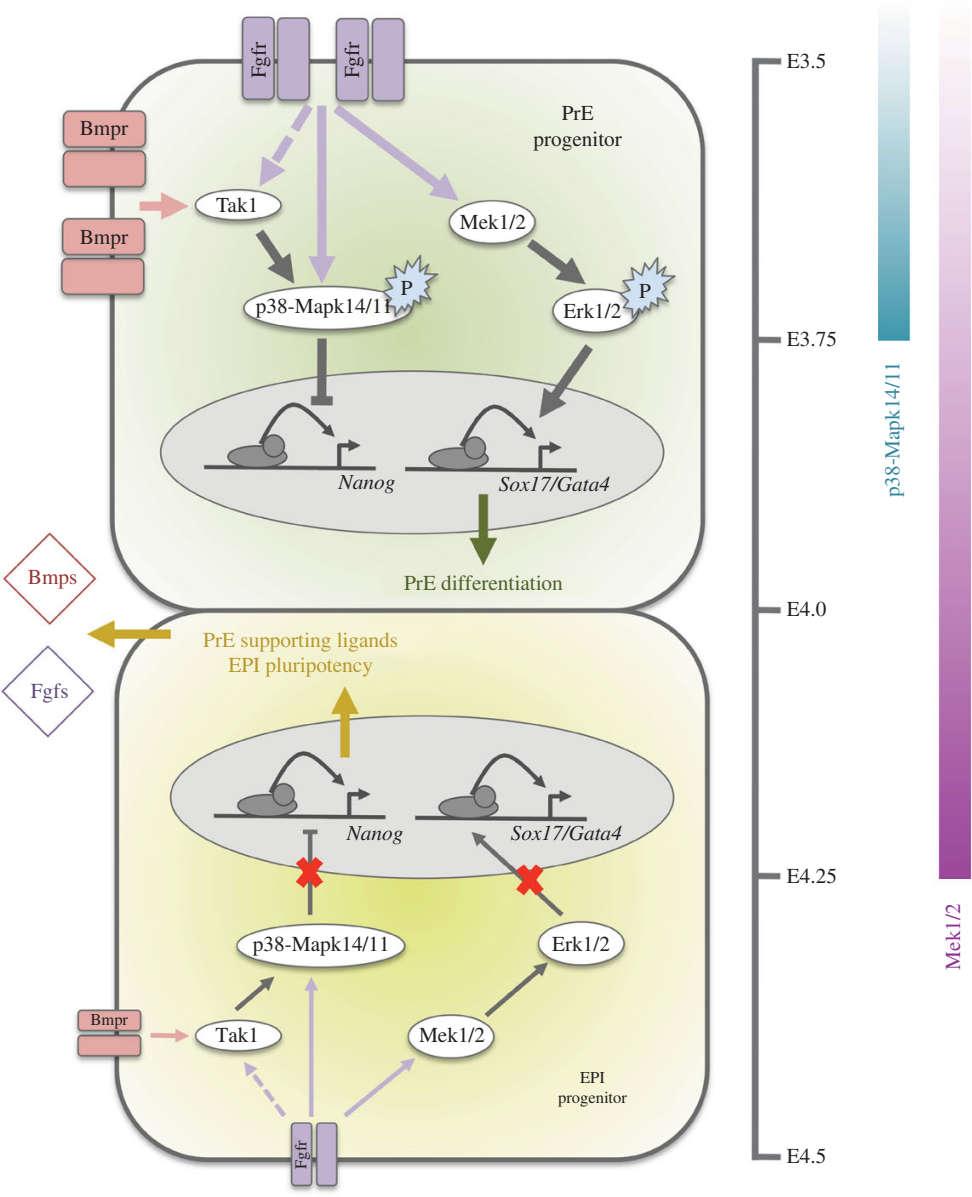
Revised model of PrE and EPI cell-fate specification and segregation during mouse blastocyst ICM maturation. In PrE progenitors, activated/liganded Bmp- and Fgf-receptors cause the activation of p38-Mapk14/11, via a mechanism at least partly dependent on Tak1 (note, theorized activation of Tak1 functionally downstream of Fgf-receptors is denoted by dashed line). Activation of p38-Mapk14/11, before E3.75, inhibits the expression of the pluripotency marker Nanog. Simultaneously, and for a period after E3.75 (until E4.25), activation of Mek1/2 and downstream Erk kinases (Erk1/2), also functionally downstream of Fgf-receptor signalling, promotes the expression of PrE markers (e.g. *Sox17* and *Gata4*) required to drive PrE cell-fate. Hence, the combined effect of activating both p38-Mapk14/11 and Mek1/2 contributes to the emergence of the so-called salt and pepper pattern of exclusive PrE and EPI marker protein expression, that arises from initially uncommitted cells expressing both *Nanog* and the early PrE marker *Gata6*, at around the E3.5–E4.0 developmental window. Ultimately, the derived salt and pepper pattern resolves into the two segregated ICM lineages by the late-blastocyst (E4.5) stage. Alternatively, in EPI progenitors an insufficiency of p38-Mapk14/11 and Mek1/2 activating signalling (due to relatively reduced expression levels of Fgf- and Bmp-receptors) fails to block Nanog or induce required PrE-related gene expression, respectively. Consequently, Nanog levels remain high and PrE differentiation is resisted in favour of retention of pluripotency. This effect also augments the expression of secreted Bmp- and Fgf-related ligands that further reinforce the promotion of PrE differentiation of neighbouring receptive cells, in a paracrine manner.

Collectively, we have demonstrated that p38-Mapk14/11 is required during mouse blastocyst ICM maturation to appropriately ensure the specification and segregation of the EPI and PrE lineages. Specifically we showed that functional p38-Mapk14/11 ensures the resolution of cells of uncommitted cell-fate, expressing both Nanog and earlier PrE markers (e.g. Gata6 and Sox17), in a developmental time window coinciding with the earliest stages of ICM maturation (i.e. before the E3.75 stage) and the ordinarily observed adoption of the classical salt and pepper pattern of mutually exclusive EPI and PrE marker expression. Moreover, we demonstrated that it is the resolution of uncommitted cells towards the PrE fate that is most severely affected. Indeed, we showed that the regulation of p38-Mapk14/11 activity, and hence function, is downstream of already known and well-characterized PrE-promoting cell signalling pathways represented by Bmpr/Tak1 and Fgfr, and that in the case of the latter, p38-Mapk14/11 probably cooperates with the Mek1/2-activated component of the same pathway to allow the driving of PrE cell-fate (see model; figure 6).

## 4. Discussion

At the early-blastocyst (E3.5) stage, the ICM of mouse embryos presents as a population of apparently bi-potent cells expressing both EPI (Nanog) and PrE (Gata6) markers. Although the extent to which all cells are indeed truly bipotent or biased towards one or the other ICM cell lineages by their previous developmental history (e.g. by the relative timing of ICM founder cell internalization) remains open to debate [18,40,49–51] and further research. However, by the mid-blastocyst stage, the ICM resolves into a heterogeneous population of cells expressing mutually exclusive EPI or PrE markers (i.e. the salt and pepper pattern) that then further segregates into the deeply residing EPI population, overlaid by the blastocoel facing PrE monolayer, by the late-blastocyst (E4.5) stage. As has been extensively reviewed elsewhere [52], such ICM maturation is mediated by the antagonistic interactions of Nanog and the active Erk1/2 pathway (functionally downstream of Mek1/2), that is itself potentiated by active receptor tyrosine kinase signalling. Although many studies have elegantly demonstrated, by means of genetic knockout and chemical inhibitor based strategies, some of the key components involved, the initial mechanism that promotes the emergence of the salt and pepper state remains relatively poorly understood. In this study, we show that p38-Mapk14/11 is an important player in this context and functions prior to, or at least concomitantly with, Erk1/2 pathway activation to promote germane ICM maturation.

In our study, we find that inhibition of the p38-Mapk14/11 from E3.5 to E4.5 resulted in a severe PrE deficit. However, not withstanding such a profound PrE defect, we were nevertheless able to observe some solely Gata4-positive PrE cells (on average 2.6 cells per embryo). The reason why these cells appear insensitive to the administered p38-Mapk14/11 inhibition regime is not clear. However, it may be explained by such cells having already passed a temporal PrE commitment point, governed by p38-Mapk14/11, by the developmental time the inhibitor was provided, a commitment point not passed for the remaining ICM cells. Indeed, to avoid causing arrested development at the morula to blastocyst transition (as observed when we provided the p38-Mapk14/11 inhibitor from the eight-cell/E2.5 stage, see electronic supplementary material, figure S1 and in other reported literature [19,30,31]), we were compelled to provide the inhibitor from the E3.5 developmental timepoint at the earliest (i.e. after specification of the TE and formation of the blastocoel). It is therefore possible that the observed p38-Mapk14/11 insensitive cells had embarked on a path of PrE differentiation before E3.5, and moreover, as the activity of the Erk1/2 pathway remains unaffected by p38-Mapk14/11 inhibition (electronic supplementary material, figure S4), such cells were free to respond to these PrE differentiation promoting cues. The fact that a couple of cells, on average, were insensitive to the p38-Mapk14/11 inhibition also supports the notion that functionally significant heterogeneity among cells of the same embryo ICM already exists by the early-blastocyst (E3.5) stage. Furthermore, that such p38-Mapk14/11 insensitive cells may have already committed to a path towards PrE differentiation may reflect their ancestral cell history. For example, reports suggest that ICM cells derived from founder cells internalized during the eight- to 16-cell stage transition are biased to form EPI, but that if atypically large numbers of cells are internalized (i.e. greater than three) the extra cells will contribute to PrE, potentially by an Fgf4-dependent mechanism [49,50,53]. It is therefore possible the p38-Mapk14/11 inhibition insensitive cells we observe may be derived from such supernumerary inner cells derived during the eight- to 16-cell transition, that have had a comparatively longer time to integrate PrE-promoting cues and commit to their fate comparatively early.

Our experiments also showed that p38-Mapk14/11 inhibition from the early (E3.5) to late blastocyst (E4.5) stages resulted in a significant increase in the number/proportion of uncommitted ICM cells co-expressing both Nanog and Gata6 (figure 1 and electronic supplementary material, figure S5), indicating p38-Mapk14/11 activity is required to permit germane progression of ICM cells, through the mutually exclusive salt and pepper expression pattern of EPI and PrE markers, to the fully segregated tissue layers of the mature ICM. However, because this p38-Mapk14/11 inhibition regime also led to late blastocysts containing on average fewer overall and specifically fewer ICM cells, it could be argued the observed effect of increased numbers/proportion of uncommitted cells was due to a general delay in development. There are multiple lines of evidence that refute this interpretation. First, while p38-Mapk14/11 inhibited embryos on average presented with fewer ICM cells they still contained an appropriate number of EPI cells, equivalent to vehicle control-treated embryos, per unit of total ICM cell number, but fewer PrE specified and more uncommitted cells (electronic supplementary material, figure S6). Second, there are examples of embryos in the p38-Mapk14/11 inhibited group that do contain equivalent numbers of ICM cells compared with controls, indicating similar developmental progression, yet these also present with significantly greater numbers of uncommitted cells, fewer PrE specified cells and equivalent EPI-specified cells (electronic supplementary material, figure S6). Third, when the p38-Mapk14/11 inhibition was given between the early (E3.5) and mid (E4.0) blastocyst stages and ICM lineage marker expression immediately assayed (figure 3), the p38-Mapk14/11 inhibited embryos comprised significantly enhanced levels of uncommitted/co-expressing cells, versus the vehicle-treated controls, despite presenting with ICMs of statistically equivalent size. Fourth, the incidence of reduced ICM cell number in p38-Mapk14/11-inhibited embryos is also accompanied by increased incidence of apoptotic cell death in both the ICM and TE (electronic supplementary material, figure S7), potentially accounting for the smaller observed numbers, rather than generally delayed development. Indeed, the total number of ICM cells in the embryos treated with vehicle DMSO control from the early (E3.5) to either mid (E4.0) or late (E4.5) blastocyst stages is statistically equal (approx. 22 cells, indicating a lack of ICM cell division during this 12 hour period), and this number is statistically equal to that observed in p38-Mapk14/11 inhibited embryos cultured to the mid (E4.0) blastocyst stage; thus, the reduced number of ICM cells associated with prolonged p38-Mapk14/11 inhibition to the late (E4.5) blastocyst stage (approx. two to three cells versus DMSO control) is most probably accounted for by the observed increase in ICM apoptosis causing a loss of cells and not generally delayed development. Moreover, when we repeated SB220025 mediated p38-Mapk14/11 inhibition experiments (from E3.5 to E4.5) using lower concentrations of the compound (i.e. 5 or 10 µM versus the originally used 20 µM, employed in previous studies [30]), we observed not only similarly reduced numbers of Gata4 positive specified PrE cells (electronic supplementary material figure S14 and tables S13) as before, but also that the average ICM cell number was not significantly reduced versus pair-matched DMSO controls. Hence, we interpret the data as truly indicating a p38-Mapk14/11 mediated role for resolving the fate of uncommitted ICM cells towards the PrE.

We report that the timing of the p38-Mapk14/11 inhibition-sensitive developmental window, in relation to separation of ICM cell lineage fate, overlaps with the earliest stages of blastocyst maturation, becoming insensitive by the E3.75 stage (although as acknowledged above, it may also precede the E3.5 stage, but the observed inhibitor-induced morula-to-blastocyst transition block, associated with temporally earlier drug administration, precludes a direct assay). Moreover, we showed that a pulse treatment of p38-Mapk14/11 inhibition between the early (E3.5) and mid (E4.0) blastocyst stage is sufficient to achieve the same PrE/ICM defects at the late (E4.5) blastocyst stage as observed by continual inhibition. However, we also discovered that during this same developmental window, Mek1/2 activity appears dispensable, as evidenced by the fact embryos can be cultured in Mek1/2 inhibitor between the early (E3.5) and mid (E4.0) blastocyst stages and then released back into normal growth media and still correctly specify and segregate the late (E4.5) blastocyst ICM cell lineages (figure 2). Moreover, we found that Mek1/2 inhibition-sensitive effects on PrE derivation were also observable (although progressively weaker) at developmental timepoints after which p38-Mapk14/11 inhibition had no effect, for example when the drug was administered from E3.75 or E4.0 to the late-blastocyst (E4.5) stage (figure 2), in agreement with previous data combining Mek1/2 and Fgfr inhibition in similar experiments [18]. It is of note that the emergence of p38-Mapk14/11 inhibition insensitivity at E3.75 is temporally coincident with the initial appearance of the salt and pepper expression pattern of mutually exclusive EPI (Nanog) and PrE (Gata6/Sox17) markers [21]. Moreover, p38-Mapk14/11 inhibition also results in statistically significant elevated levels of uncommitted cells (be it at the mid/E4.0 or late/E4.5 blastocyst stages, figures 3 and 1 and electronic supplementary material, figure S5, respectively) that are apparently unable to fully differentiate (evidenced by the substantially reduced numbers/ICM proportion of cells expressing the late PrE marker, Gata4, at the late-blastocyst stage, figures 1 and 2 and electronic supplementary material, figure S3). Such uncommitted cells appear to resist differentiation, despite the presence of a non-inhibited and temporally downstream active Mek1/2 (Erk1/2) pathway (*n.b.* p38-Mapk14/11 inhibition does not alter levels of activated Erk1/2(p), electronic supplementary material, figure S4), known to ordinarily induce PrE formation. Given that the uncommitted cells by definition express Nanog, and that continued Nanog expression in mouse embryos and ES cells is known to prevent differentiation [8,54], our data support a role for p38-Mapk14/11 in governing the commitment of ICM cells to fully enter into the PrE differentiation programme by downregulating Nanog expression and becoming receptive to Mek1/2-directed mechanisms of differentiation. Indeed, other ES cell studies have identified just such a role for p38-Mapks in regulating entry into cardiac or neurogenic differentiation [28] or being the targets for functional Bmp4-derived inhibition, resulting in the promotion of self-renewal [29]. Moreover, other reports have shown that small molecule inhibition of p38-Mapks results in the promotion of naive pluripotency in primate and human stem cell cultures, *in vitro* [55–57]. Interestingly, although we found that functional Mek1/2 sensitivity temporally extended beyond that of p38-Mapk14/11, we did find that the extreme PrE deficit phenotype (resulting in a virtual absence of Gata4 positive ICM cells) observable after prolonged Mek1/2 inhibition alone (from either the eight-cell/E2.5 or early/E3.5 to late/E4.5 blastocyst stages, figure 2 and electronic supplementary material, figure S15, respectively) could be partially rescued using the microinjected, p38-Mapk14/11 activating, Mkk6-EE mRNA construct (electronic supplementary material, figure S15). Such data imply that while Mek1/2 inhibition is very successful in blocking full PrE differentiation to Gata4-alone expressing ICM cells, some otherwise uncommitted cells, co-expressing Nanog and early PrE markers (e.g. Gata6), may exist after Mek1/2 inhibition and that enhanced levels of p38-Mapk14/11, caused by Mkk6-EE expression, may be sufficient to drive these cells to fully differentiate to PrE. Consistent with this theory, we find that after Mek1/2 inhibition from the early (E3.5) to late (E4.5) blastocyst stages, the ICM of treated embryos do indeed contain a number of uncommitted Nanog and Gata6 co-expressing cells (electronic supplementary material, figure S16). In addition, it is interesting that the profound PrE-deficit phenotypes associated with Mek1/2 inhibition are associated with large increases in EPI number/proportion, despite the presence of non-inhibited p38-Mapk14/11. Therefore, it is probable that some ICM cells that had resolved from their uncommitted state, by the action of p38-Mapk14/11 causing reduced Nanog levels, may reactivate *Nanog* expression as a default response to the lack of active and Mek1/2-driven PrE differentiation.

The role of Mek1/2 in activating the Erk1/2 pathway downstream of Fgfr activation during the emergence of PrE has been well described [15–18], but the data presented here suggest that activation of the same receptor also leads to functional activation of p38-Mapk14/11 that is relevant for full entry into PrE differentiation. Indeed, a retrospective review of the literature supports a mechanism by which activated Fgfr activates two distinct mitogen-activated protein kinase pathways during PrE differentiation. For example, it is known that the ICMs of *Fgf4^−/−^*null embryos fail to express Gata6 or a transgenic Pdgfrα promoter-driven early PrE reporter gene at the early-blastocyst (E3.5) stage, indicative of a block in PrE specification. However, if wild-type embryos are treated with Mek1/2 inhibitor from the eight-cell (E2.5) stage in combination with a Gsk3β inhibitor, they are able to express the same early PrE reporter gene [16]. Hence, the functional inhibition of just one component downstream of the Fgfr is not able to phenocopy the absence of the activating ligand, thus suggesting the existence of at least one other functional pathway acting beneath the receptor itself, most likely to involve p38-Mapk14/11 as described here. Indeed, there are numerous non-preimplantation mouse embryo-related studies reporting incidences of p38-Mapk pathway activation consequent to Fgf-based signalling [58–60], and more recently, Fgfr2-mediated Fgf2 signalling has been demonstrated to depend on the p38-Mapk pathway to regulate TE development in mouse preimplantation embryos [19]. Furthermore, genetic evidence from models of T-cell activation suggest that Grb2, most often considered in the preimplantation mouse embryo context for its potential role of activating the Erk1/2 pathway (via Mek1/2) to promote PrE differentiation in the ICM [21], can also activate p38-Mapks (plus Jnks) in a manner that is preferential to its activation of Erk1/2 [61]. Hence, there exists precedent for Fgfr-mediated activation of the p38-Mapk pathway, from both the earlier stages of preimplantation mouse embryo development and other cellular/developmental systems. Moreover, these precedents are similar to the Fgfr results described, in conjunction with Mek1/2 (Erk1/2) activation and in the context of ICM cell-fate resolution, herein. Interestingly, we found that chemical inhibition of Fgfr in preimplantation embryos only reduced, rather than abolished, detectable levels of activated p38-Mapk14/11(p) (electronic supplementary material, figure S10), suggesting multiple activating inputs are in play, as would be in keeping with the known general biology of p38-Mapks [27]. Indeed, we find that chemical inhibition of the catalytic activity of the serine/threonine kinase Tak1 (also known as Mekk7/Map3k7) also reduces the levels of activated p38-Mapk14/11(p) in the preimplantation mouse embryo (electronic supplementary material, figure S10). Moreover, Tak1 has previously been described as being a component of the non-canonical and Smad-independent pathway of Bmpr-mediated/directed PrE differentiation in mouse blastocyst ICMs [20], and the results of the Tak1 inhibition (using 5*Z*-7-Oxo) presented here (figure 5) are consistent with this earlier study. Despite having a broad range of substrate specificity, Tak1 is known in other cell contexts to target and activate the p38-Mapk14/11 specific activating kinases Mkk6 and Mkk3 [46,62], suggesting that it is by failing to activate one or both of these intermediate kinases that Tak1 inhibition leads to reduced levels of activated p38-Mapk14/11(p); this assertion is substantiated by the fact that PrE deficits caused by Tak1 inhibition can be rescued by overexpression of the constitutively active Mkk6-EE mutant (figure 5). Hence, it would appear that p38-Mapk14/11 activation, and the attendant full entry of mouse blastocyst ICM cells into PrE differentiation, is under the control of at least two independent cell signalling pathways, based on secreted Fgf- and Bmp-ligands. It is noteworthy, however, that activated Fgfr3-based signalling has been shown to be Tak1 dependent in the context of multiple myeloma and bladder cancers [63], therefore not excluding the possibility that mouse blastocyst Fgf-signalling may also activate Tak1 (indicated by the dashed arrow in the model present in figure 6).

Activated p38-Mapk14/11(p) have been implicated in the phosphorylation of a plethora of downstream target proteins, in a wide range of cellular contexts, with functional consequences for such fundamental processes as protein turnover, transcription and chromatin remodelling and cytoskeleton structure [27]. With such a broad spectrum of potential downstream functional effects, it is, and has proved, difficult to identify those responsible for the p38-Mapk14/11 inhibition-mediated effects we observe, in relation to ICM cell-fate specification and segregation. As discussed above, the phenotype we observe is best described as a block of uncommitted cells to resolve their fate and, in particular, fully initiate their differentiation towards PrE. Consequently, we have conducted a series of experiments (not described here), with this commitment block in mind, to try and resolve the mechanism behind the p38-Mapk14/11 inhibition phenotype, but to no avail. These include assaying for the relative abundance of phospho-specific forms of Nanog (Nanog(p)), that in ES cells has been shown to destabilize the protein and thus potentiate differentiation (by the action of Erk1/2, [64]), but no differences could be observed between control and p38-Mapk14/11 inhibited embryo groups (although we did not have all the necessary antisera to probe all the known Nanog(p) isoforms). Similarly, we could find no differences in the protein expression of Zscan10, a known ES cell pluripotency-related factor [65] whose paralogous gene, *Mzf1*, is listed as a p38-Mapk14 substrate in the ‘phosphosite.org’ database. We also tried overexpressing the known p38-Mapk14/11 effector kinase, 3pK (also known as Mapkapk3), in an attempt to overcome the p38-Mapk14/11 inhibition-mediated uncommitted ICM cell phenotype. This was because 3pk has been shown to phosphorylate the Gata6 interacting and stabilizing poly-comb group (PcG) protein Bmi1 [66], leading to its instability and degradation [67]; hence, we speculated p38-Mapk14/11 inhibition might be stabilizing Bmi1 and so Gata6, thus contributing to the uncommitted cell fate state, by a failure to activate 3pK. However, overexpression of constitutively activated 3pK had no effect on the p38-Mapk14/11 inhibition-mediated ICM phenotype. We also attempted to rescue the phenotype by overexpressing a constitutively activated form of another confirmed p38-Mapk14/11 effector, Msk1, known to promote transcriptional activation (potentially of target genes required for full entry into PrE differentiation) by phosphorylating chromatin on histone H3 (at serines 10 and 28) to repel PcG protein binding [68]. However, Msk1 overexpression was associated with cell division arrest and direct pharmacological inhibitory targeting of the Msk1 protein, during blastocyst maturation, had no effect on ICM lineage specification and segregation. Similarly, the overexpression of the histone acetyl-transferase Atf2 (actually a constitutively active fusion protein of human ATF2 linked to the transcriptional activation domain of CREB [69]), a well-characterized downstream p38-Mapk14/11 effector [70], was also unable to rescue the p38-Mapk14/11 inhibition-mediated uncommitted ICM phenotype. Lastly, given that PrE-like differentiation in F9 teratocarcinoma cells was shown to require p38-Mapk14/11 mediated inhibition of Gsk3β function [35], we tested if our observed uncommitted ICM phenotype could be rescued by direct pharmacological inhibition of Gsk3β itself and found that it could not. As referenced above, the potential wide range of downstream p38-Mapk14/11 effectors and pathways makes it difficult to identify any one in isolation as a candidate for the observed ICM specification defects we report. Notwithstanding, we have made a substantial and concerted effort to mechanistically assay the components functionally downstream of p38-Mapk14/11 activation. We accordingly suggest that the failed attempts to rescue the observed ICM phenotype, as described above, may largely reside in the fact that p38-Mapk14/11 touches many related and important effector pathways and that the perturbation of just one of many may not be sufficient to reveal a mechanistic role.

In conclusion, we report how Fgfr and non-canonical Bmpr/Tak1-mediated cell signalling converge upon and activate p38-Mapk14/11 during a critical early window of blastocyst maturation contributing to the germane specification and segregation of the ICM cell lineages. We also show how this activation is indispensable and required for the resolution of uncommitted cells to begin their full differentiation towards a PrE fate, concomitantly and then subsequently facilitated by the classically appreciated active Mek1/2 (and hence Erk1/2) signalling pathway. Therefore, within the context of the maturing mouse blastocyst ICM, the activation of p38-Mapk14/11 can be considered as the enabler of PrE differentiation, while activation of Mek1/2 is the driver (summarized in figure 6). It will be of great interest to uncover the functionally downstream mechanisms by which p38-Mapk14/11 activity executes this enabling role in future studies.

## Supplementary Material

TV_2016_supp_data
