# Supplementary material for: p38 (Mapk14/11) occupies a regulatory node governing entry into primitive endoderm differentiation during preimplantation mouse embryo development
Source: Open Biol. 2016 Sep 7;6(9):160190. doi: 10.1098/rsob.160190 (PMC5043583; doi:10.1098/rsob.160190)
Supplement: TV_2016_supp_data [file rsob160190supp1.pdf]

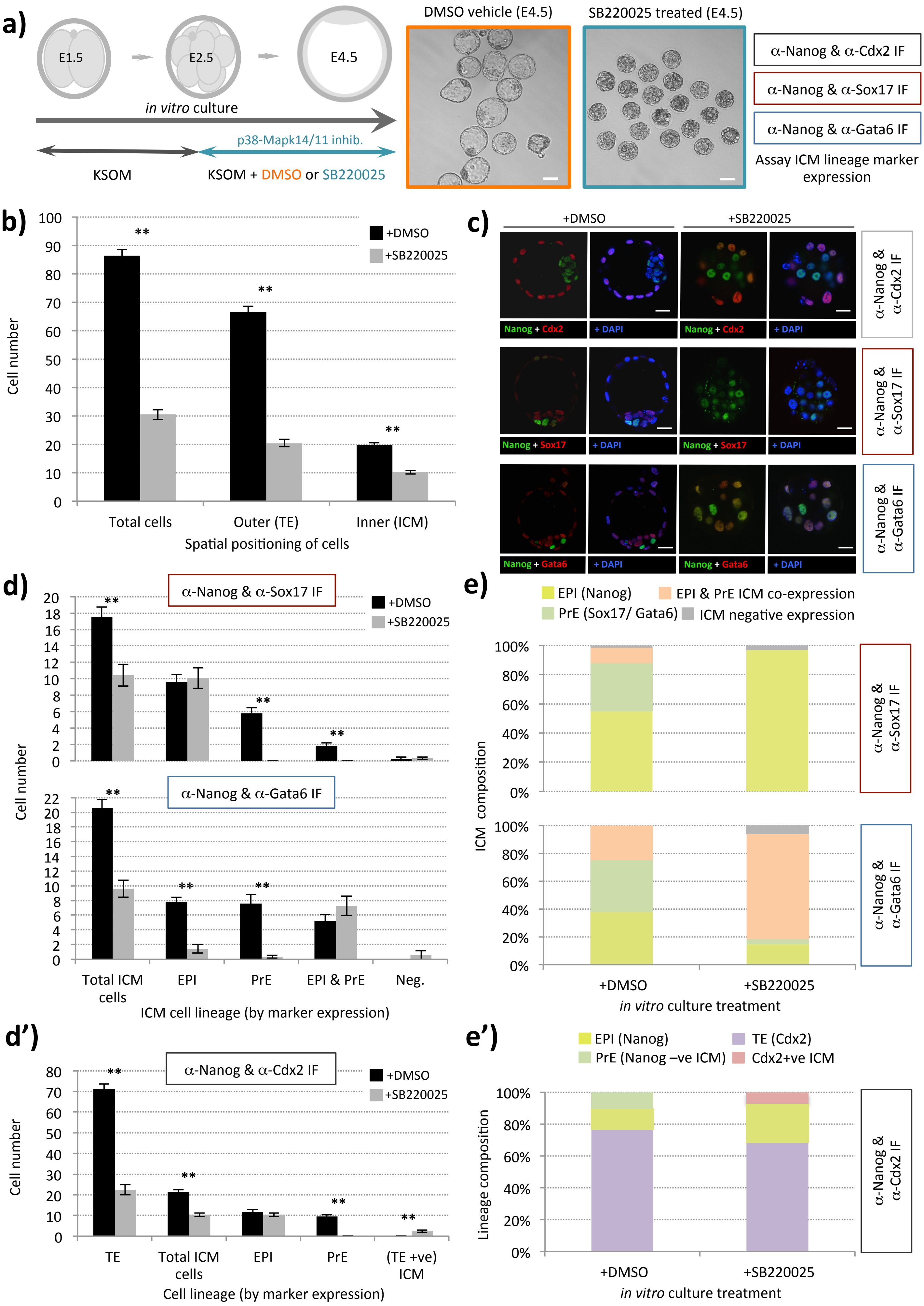

Supplementary figure S1.

Supplementary figure S1: p38-Mapk14/11 inhibition from the 8-cell (E2.5) stage. a) Experimental schema of *in vitro* cultured embryos subject to p38-Mapk14/11 inhibition (SB220025; highlighted in sea-green), or vehicle control treatment (DMSO; highlighted in orange), from the 8-cell (E2.5) to late-blastocyst (E4.5) stage and the details of the antibodies used to analyse ICM cell lineage marker protein expression by immuno-fluorescence (IF); denoted by grey box, Nanog and Cdx2 (+DMSO n=14, +SB220025 n=18), denoted by red box, Nanog and Sox17 (+ DMSO n=15, + SB220025 n=15) and denoted by blue box, Nanog and Gata6 (+DMSO n=13, +SB220025 n=12). Bright-field micrographs detail exemplar late-blastocyst (E4.5) stage embryos at the end of the *in vitro* culture/ inhibition period, prior to fixation and IF. Note defective blastocoel formation in SB220025 group. Scale bar = 50μm. **b)** Average number of outer- (defined as TE) and inner- (ICM) cells in embryos stained by IF for indicated ICM markers [*i.e.* as in **a)**] in either control (+ DMSO, black bars) or p38-Mapk14/11 inhibited (+ SB220025, grey bars) conditions. Error bars reflect s.e.m and statistically significant divergence between conditions, determined by 2-tailed student t-test, are highlighted (* $p<0.05$ and ** $p<0.005$). Data represent the composite of the three IF regimes employed. **c)** Representative single z-plane confocal micrographs of embryos stained by IF for the indicated ICM makers [*i.e.* as in **a)**] in control (+ DMSO) or p38-Mapk14/11 inhibited (+ SB220025) conditions. Nanog derived staining in green, Cdx2, Sox17 and Gata6 signal in red are shown in merged images with additional DNA DAPI counterstain in blue. Scale bars = 15μm. **d)** and **d')** Bar charts showing average number of cells allocated to each specified ICM lineage, as judged by the indicated IF staining regime employed [as in **a)**]. Error bars represent s.e.m. and * and ** denote statistical significant differences in cell number between the vehicle control (+DMSO, black bars) and p38-Mapk14/11 inhibited (+SB220025, grey bars) embryo groups, according to 2-tailed students t-test, with $p<0.05$ and $p<0.005$ confidence intervals, respectively. Note ICM cells solely expressing Nanog or Sox17/ Gata6 were classified as EPI or PrE respectively or EPI & PrE if both markers were detectable. Cells negative for both ICM markers were designated negative (Neg.). A similar classification was applied to the embryo group immuno-stained for Nanog andCdx2 [see **d')**], although PrE cells were classified as those ICM cells expressing neither Nanog or Cdx2 and instead of Neg. cells, ICM cells exhibiting ectopic expression of Cdx2 were noted (TE +ve ICM). The total number of outer-residing Cdx2 positive TE cells is additionally provided. **e)** and **e')** Percentage bar charts detailing the averaged relative cell lineage composition of ICMs from vehicle control (+DMSO) and p38-Mapk14/11 inhibited (+SB220025) embryo groups, [classified according to the scheme described for **d)** and **d')**], immuno-stained for the indicated cell lineage marker proteins. All individual embryo data used in the preparation of this figure are contained within supplementary tables ST1 and ST2.

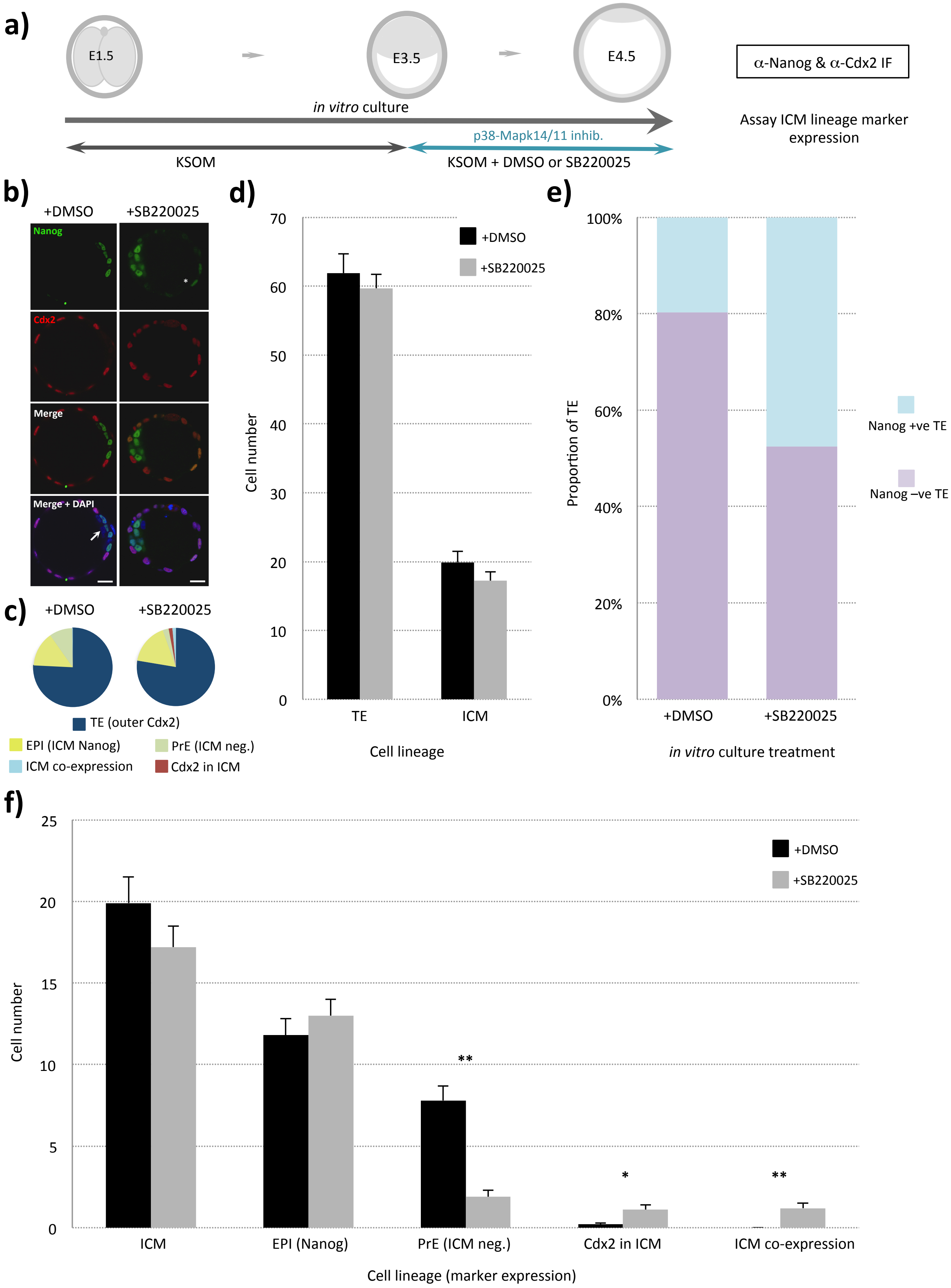

Supplementary figure S2.

Supplementary figure S2: p38-Mapk14/11 inhibition from the early- (E3.5) to late- blastocyst (E4.5) stage. a) Experimental schema of *in vitro* cultured embryos subject top38-Mapk14/11 inhibition (+SB220025) or vehicle control treatment (+DMSO) from the early- (E3.5) to late-blastocyst (E4.5) stage and immuno-fluorescently (IF) stained for Nanog and Cdx2 (+DMSO n=17, +SB220025 n=26). **b)** Representative single z-plane confocal micrographs of IF stained embryos in control (+ DMSO) or p38-Mapk14/11 inhibited (+ SB220025) groups. Nanog derived staining in green, Cdx2 signal in red (also merged) plus additional merged image with DNA DAPI counterstain in blue. Scale bars = 15μm. Note, arrow in the control image (+DMSO) denotes ICM cells devoid of detectable Nanog signal, designated as PrE; asterisk denotes ectopic Nanog expression in the outer TE cells of p38-Mapk14/11 inhibited embryos. **c)** Pie charts of the relative cell lineage contribution in vehicle control (+DMSO) and p38-Mapk14/11 inhibited (+SB220025) blastocyst as judged by anti-Nanog/ Cdx2 IF. Blue = trophectoderm (TE), yellow = Epiblast (EPI – ICM exhibiting exclusive Nanog expression), green = Primitive Endoderm (PrE - ICM devoid of staining), light blue = co-expressing ICM cells (exhibiting co-expression of both Nanog and Cdx2) and maroon = ICM cells ectopically expressing Cdx2. **d)** The averaged number of outer/ TE and inner/ ICM cells in p38-Mapk14/11 inhibition (SB220025; grey bars) or vehicle control treatment (DMSO; black bars) groups. Errors stated as s.e.m. **e)** Percentage bar charts detailing the averaged relative ectopic expression of Nanog in outer TE cells in p38-Mapk14/11 inhibition (+SB220025) or vehicle control treatment (+DMSO) groups. Light blue denoting ectopic expression in the TE and purple an absence of detectable TE Nanog. **f)** Bar chart showing average number of cells allocated to each indicated ICM lineage, as judged by the indicated IF staining pattern detected. Error bars represent s.e.m. and * and ** denote statistical significant differences in cell number between the vehicle control (+DMSO, black bars) and p38-Mapk14/11 inhibited (+SB220025, grey bars) embryo groups, according to 2-tailed students t-test, with $p<0.05$ and $p<0.005$ confidence intervals, respectively. All individual embryo data used in the preparation of this figure are contained within supplementary tables ST4.

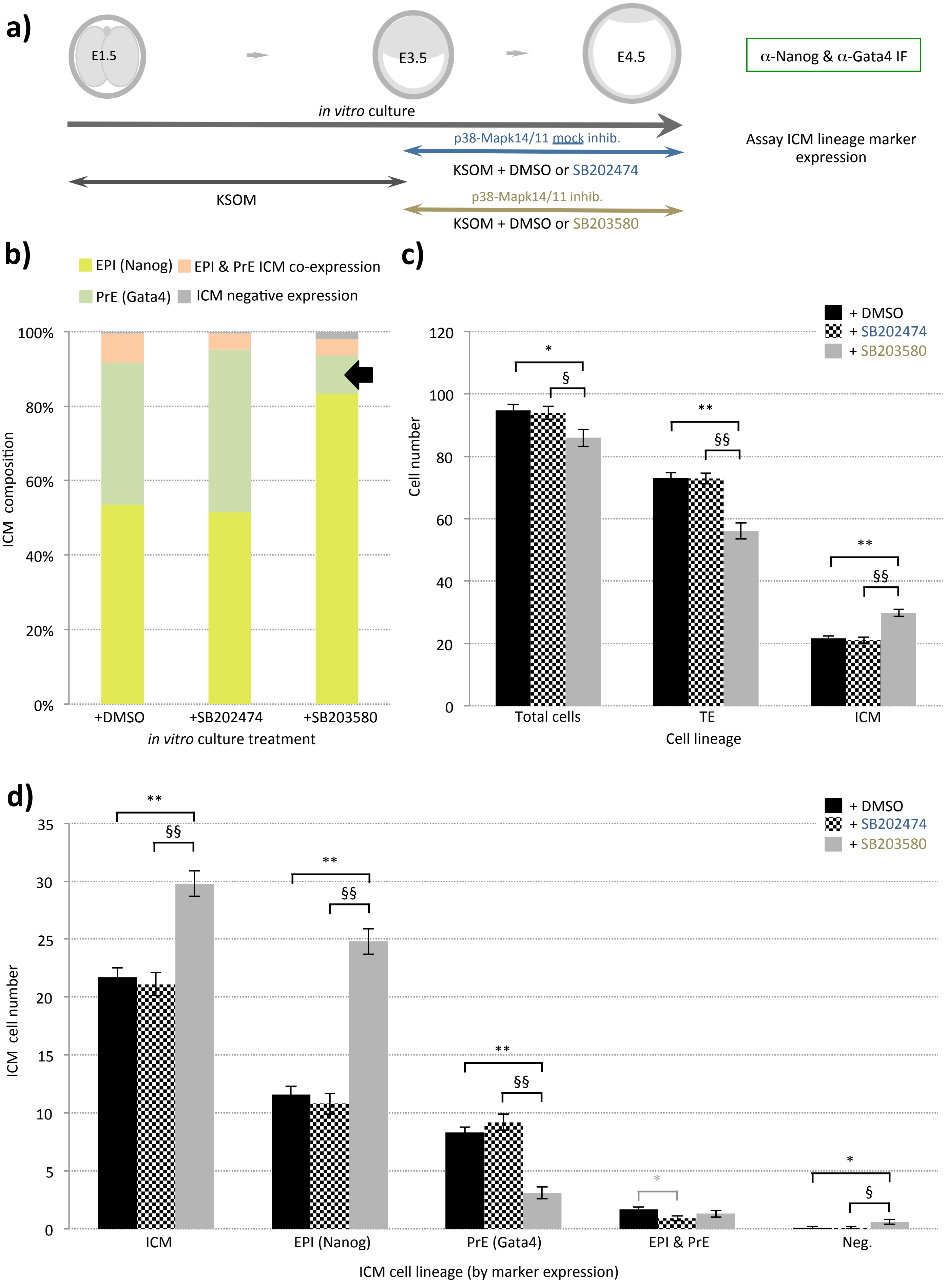

Supplementary figure S3.

Supplementary figure S3: The alternative p38-Mapk14/11 inhibitor SB203580 also impairs PrE formation in the ICM (when administered from E3.5 to E4.5) whereas its inactive analog SB202474 does not. a) Experimental schema detailing the regime of p38-Mapk14/11 inhibition (+SB20350) or mock inhibition with inactive drug analog (+SB202474), at 20μM concentrations, with attendant vehicle control (+DMSO) condition, from the early to late blastocyst (E3.5-E4.5) stages employed. Immuno-fluorescence (IF) antibody details used to analyse ICM cell lineage marker protein expression in late blastocysts (E4.5) are also given (*n.b.* Gata4 used as PrE marker). **b)** Averaged percentage makeup of the ICMs of each stated condition in relation to each specified ICM lineage; EPI or PrE (yellow and green, exclusively immuno-stained for either Nanog or Gata4, respectively), EPI & PrE co-expressing cells (orange, representing cells uncommitted to either lineage) and cells negative for either studied lineage marker (grey). The black arrow denotes the decreased percentage contribution of Gata4–alone positive PrE cells in the ICM of SB203580 treated embryos compared to +DMSO vehicle or inactive analog (+SB202474) controls. In the +DMSO treated group n = 32, in the +SB202474 mock group n = 19 and in the +SB203580 p38-Mapk14/11 inhibition group n = 18. **c)** Averaged total cell number and contribution to the TE and ICM blastocyst cell lineages, based on relative spatial location in each of the stated experimental conditions (outer; TE and inner; ICM). Errors are represented as s.e.m. and appropriate statistically significant differences, derived from 2-tailed students t-tests, highlighted by one or two significance markers (*/§ $p<0.05$ and **/§§ denoting $p<0.005$) are shown. **d)** Averaged contribution of cells to each ICM cell lineage, based on exclusive expression of either EPI (Nanog) or PrE (Gata4) lineage markers (or both or neither marker), in each of the stated experimental conditions. Errors and significance denoted as in **c).** All data used to prepare this figure are described in supplementary tables ST5.

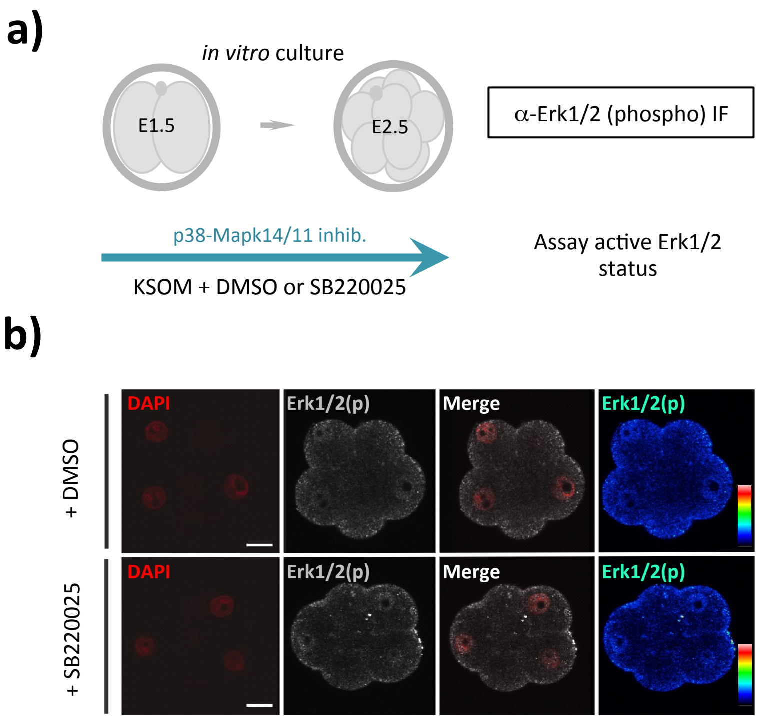

Supplementary figure S4.

Supplementary figure S4: Treatment of embryos with the p38-Mapk14/11 inhibitor SB220025 does not alter the levels of detectable activated phospho-Erk1/2 [Erk1/2(p)]. a) Experimental schema by which 2-cell (E1.5) stage embryos were *in vitro* cultured to the 8-cell (E2.5) stage in control (+DMSO) or p38-Mapk14/11 inhibitor (+SB220025) treated media, fixed and immuno-fluorescently (IF) stained for activated Erk1/2(p). **b)** Representative single central confocal z-plane sections of (IF) stained embryos from each group. Erk1/2(p) signal is shown in both grey and spectral pixel intensity palettes and red pseudo-coloured DAPI DNA counterstain is also provided (merged and unmerged images shown). Scale bar = 15μm.

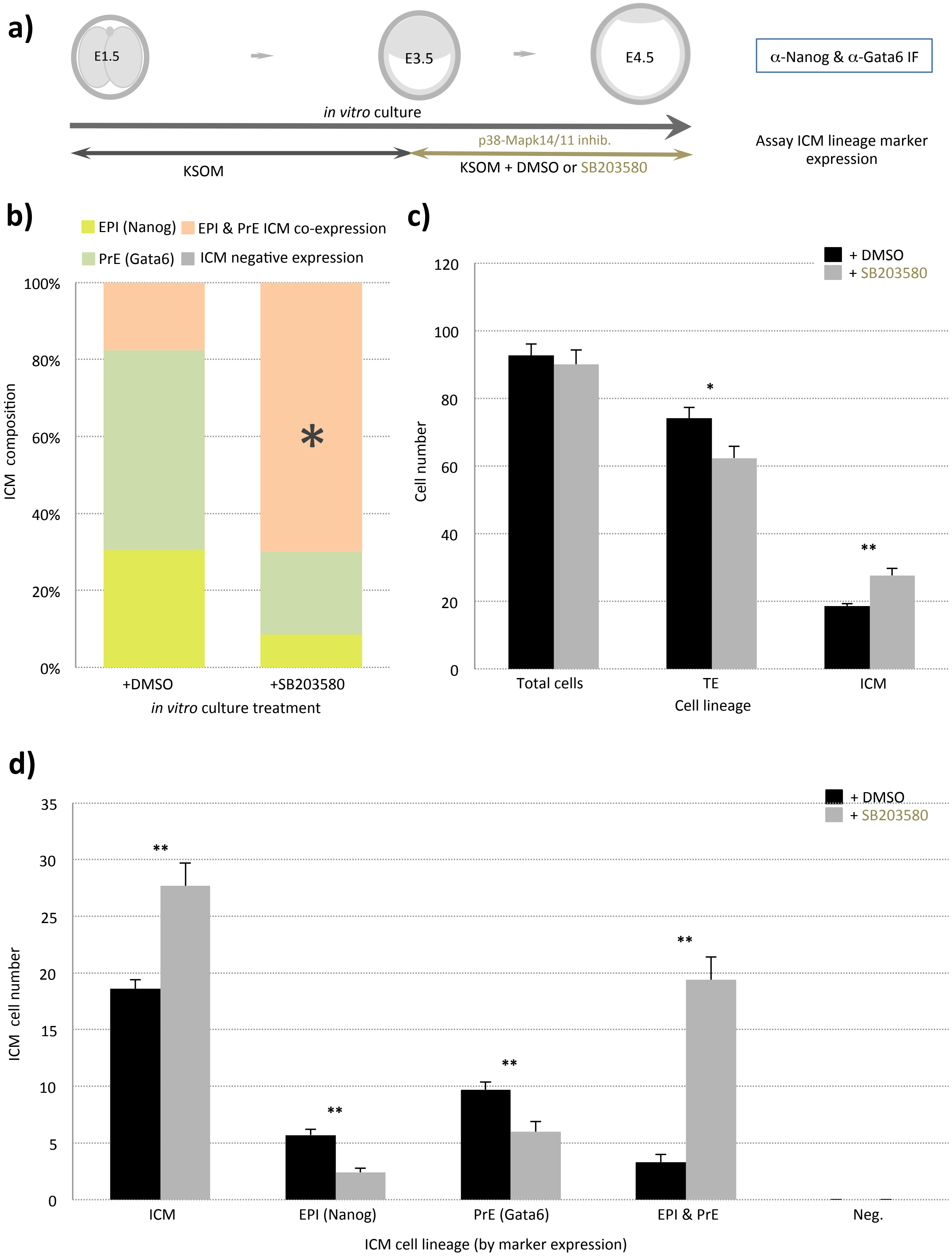

Supplementary figure S5.

Supplementary figure S5: The alternative p38-Mapk14/11 inhibitor SB203580 also causes increases in uncommitted ICM cells expressing both EPI (Nanog) and early PrE (Gata6) markers (when administered from E3.5 to E4.5) a) Experimental schema detailing the regime of p38-Mapk14/11 inhibition (+SB20350, 20μM), with attendant vehicle control (+DMSO) condition, from the early to late blastocyst (E3.5-E4.5) stages employed. Immuno-fluorescence (IF) antibody details used to analyse ICM cell lineage marker protein expression in late blastocysts (E4.5) are also given (*n.b.* Gata6 used as PrE marker). **b)** Averaged percentage makeup of the ICMs of each stated condition in relation to each specified ICM lineage; EPI or PrE (yellow and green, exclusively immuno-stained for either Nanog or Gata6, respectively), EPI & PrE co-expressing cells (orange, representing cells uncommitted to either lineage) and cells negative for either studied lineage marker (grey). Asterisk denotes the increased percentage contribution of uncommitted ICM cells (expressing both Nanog and Gata6) in +SB203580 treated embryos versus +DMSO vehicle control. In the +DMSO treated group n=12 and in the +SB203580 p38-Mapk14/11 inhibition group n=14. **c)** Averaged total cell number and contribution to the TE and ICM blastocyst cell lineages, based on relative spatial location in each of the stated experimental conditions (outer; TE and inner; ICM). Errors are represented as s.e.m. and appropriate statistically significant differences, derived from 2-tailed students t-tests, highlighted by one or two significance markers (* $p<0.05$ and ** denoting $p<0.005$). **d)** Averaged contribution of cells to each ICM cell lineage, based on exclusive expression of either EPI (Nanog) or PrE (Gata6) lineage markers (or both or neither marker), in each of the stated experimental conditions. Errors and significance denoted as in **c).** All data used to prepare this figure are described in supplementary tables ST6

a)

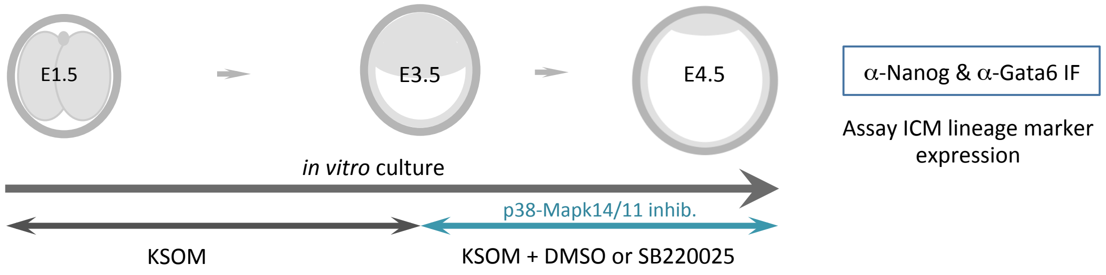

b)

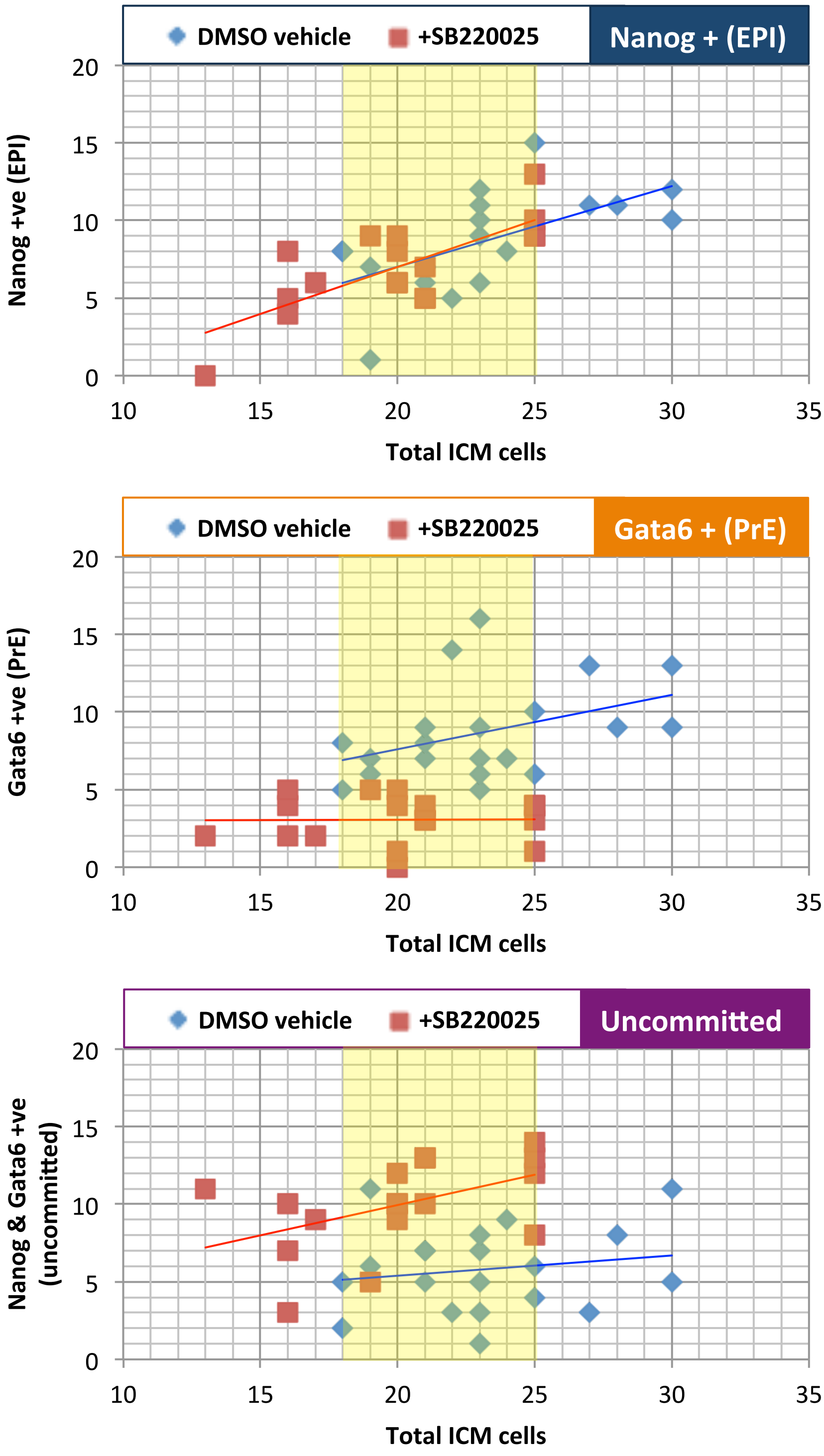

Supplementary figure S6.

Supplementary figure S6: The reduction in PrE cells induced by p38-Mapk14/11 inhibition (using SB220225, 20μM) is not due to a general and temporal delay in ICM cell-fate separation, despite smaller total ICM cell number. a) Experimental schema of the blastocyst maturation (E3.5–E4.5) p38-Mapk14/11 inhibition (+SB220025, n=15), plus vehicle control (+DMSO, n=14), strategy employed and the immuno-fluorescent (IF) staining regime used to assay resulting individual ICM cell-fates; cells exhibiting exclusive Nanog or Gata6 expression being designated EPI and PrE, respectively, and those expressing both markers denoted as uncommitted to either cell lineage. **b)** Three x versus y scatter plots, detailing the number of cells immuno-staining positive for either Nanog (EPI) or Gata6 (PrE) alone (upper-blue and central-orange plots, respectively) or both markers (representing uncommitted cells, lower-purple plot) per embryo, as a function of total ICM cell number in control (+DMSO vehicle; blue diamonds) and p38-Mapk14/11 inhibited (+SB220025; red squares). Trend lines (blue for control and red for p38-Mapk14/11 inhibited embryo groups), plus regions were the two datasets overlap in terms of total ICM cell number (yellow shaded highlights) are also shown to aid interpretation. Note, that despite p38-Mapk14/11 inhibited embryos on average presenting fewer ICM cells (see figure 1 and supplementary figure S2), they still constitute a number of EPI cells appropriate to this size, whereas they contain disproportionately fewer PrE cells and more uncommitted cells; hence the deficit in mature PrE cells observed after p38-Mapk14/11 inhibition (see figure. 1 and supplementary figure S2) cannot simply be a function of generally delayed development, as EPI cells have segregated, but is indicative of a block in the maturation of the PrE lineage.

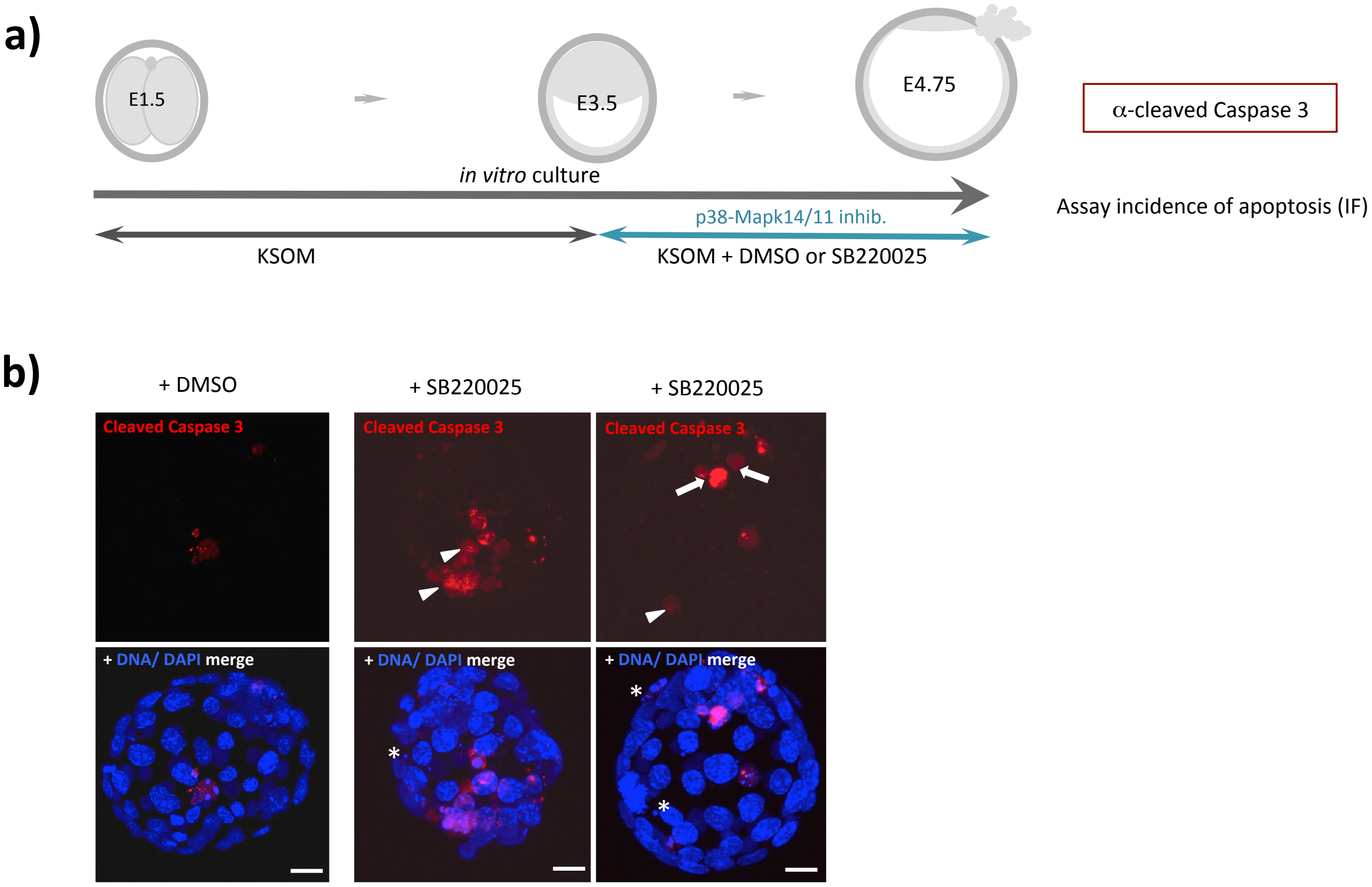

Supplementary figure S7.

Supplementary figure S7: p38-Mapk14/11 inhibition during blastocyst maturation is associated with increased incidence of apoptosis in both TE and ICM cells. a) Experimental schema by which recovered 2-cell (E1.5) stage embryos were *in vitro* cultured until the early blastocyst (E3.5) stage and transferred into media containing the p38-Mapk14/11 inhibitor SB220025 or DMSO vehicle control and further cultured to the late/ hatching blastocyst (E4.75) stage. Embryos where fixed and immuno-fluorescently stained with anti-cleaved caspase 3 antibody for confocal microscopy-based assay of the incidence of apoptosis. **b)** Exemplar confocal z-plane projections of control (+ DMSO) and p38-Mapk14/11 inhibitor treated (+ SB220025) blastocysts; cleaved caspase signal in red and DNA DAPI counterstain in blue. Arrows and arrow-heads highlight apoptotic cells, positive for cleaved caspase 3, in the outer TE and ICM cells, respectively, of Mapk14/11 inhibited embryos. Asterisks in + DNA/ DAPI merge micrographs highlight apoptotic bodies in Mapk14/11 inhibited embryos, that are not positive for cleaved caspase 3 immuno-staining. Scale bar = 20μm.

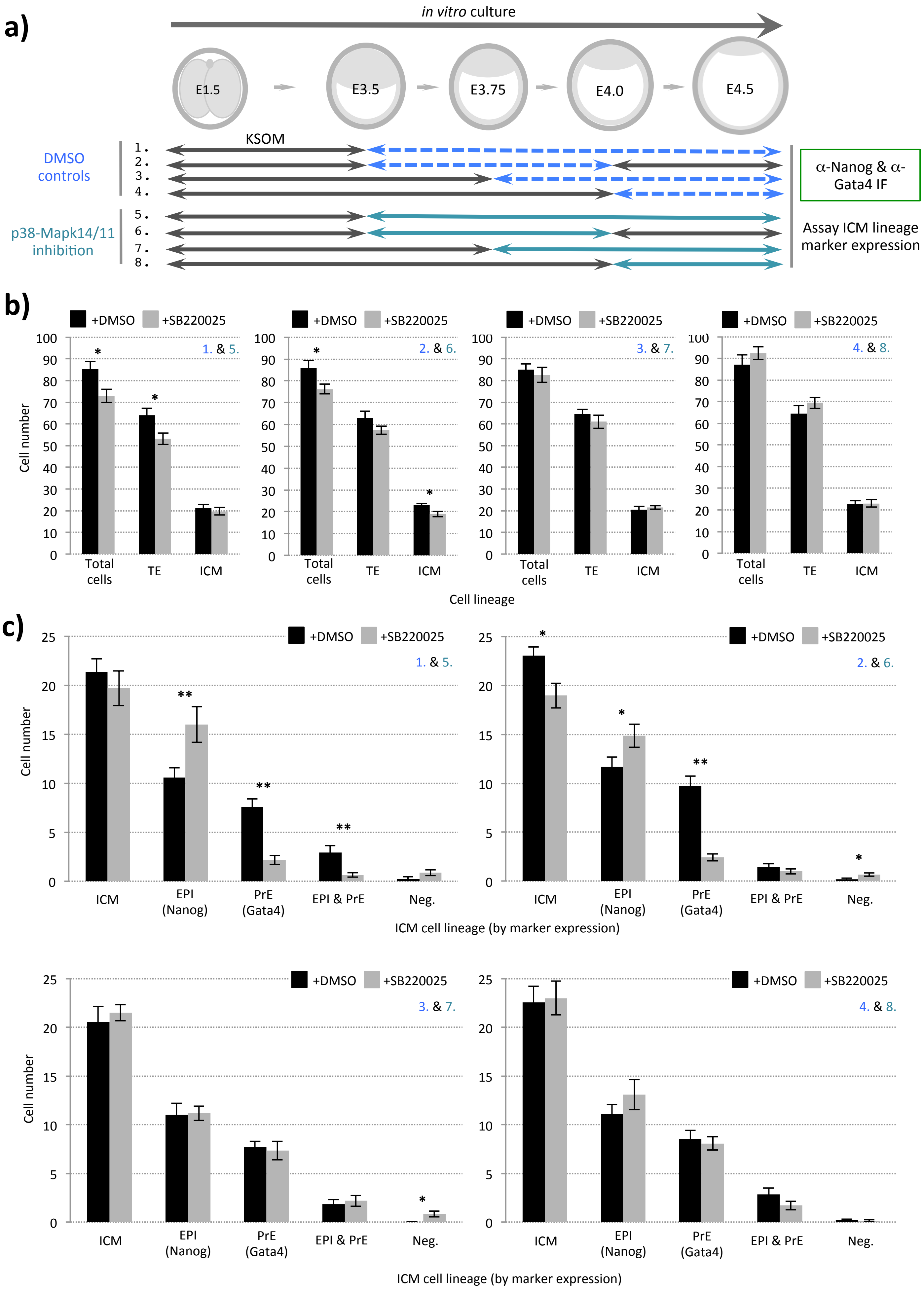

Supplementary figure S8.

Supplementary figure S8: Varied temporal inhibition of p38-Mapk14/11 activity during blastocyst maturation; averaged numbers of cells contributing to different blastocyst lineages (data supplementary to that presented in figure 2). a) Experimental schema employed to identify the developmental time-point at which p38-Mapk14/11 is required for PrE differentiation. 2-cell (E1.5) stage embryos were *in vitro* cultured to varied blastocyst stages (ranging from E3.5 to E4.0, as indicated) and transferred into media supplemented with either p38-Mapk14/11 inhibitor (SB220025) or DMSO vehicle controls (note the nomenclature of each condition, 1, 3 and 4 for DMSO controls and 5, 7 and 8 for p38-Mapk14/11). Embryos were then cultured to the late-blastocyst (E4.5) stage and fixed for immuno-fluorescent staining against the ICM cell lineage markers Nanog and Gata4. Note that a second embryo group transferred at the early-blastocyst (E3.5) stage was removed from vehicle control/ inhibitor treatment at the mid-blastocyst (E4.0) stage and returned to normal growth media before being similarly processed at the late-blastocyst (E4.5) stage (groups 2 and 6). **b)** Bar charts detailing average total cell number and number of cells allocated to each blastocyst lineage (*i.e.* outer TE or inner ICM) in each of the above described control and treatment conditions [see **a)**]. Error bars represent s.e.m. and * and ** denote statistical significant differences in cell number between the vehicle control (+DMSO, black bars) and p38-Mapk14/11 inhibited (+SB220025, grey bars) embryo groups, according to 2-tailed students t-test, with $p<0.05$ and $p<0.005$ confidence intervals, respectively. Note that the most suitable control and p38-Mapk14/11 inhibition data have been paired into each of the four subpanels [denoted by the same numbered and coloured nomenclature that is used in panel **a)**]. **c)** Similar bar charts to those in **b)**, but describing the average number of cells allocated to each blastocyst ICM lineage, as judged by detectable lineage marker gene expression [*i.e.* Total ICM cell number as in **a)**; EPI, Nanog alone; PrE, Gata4 alone; EPI & PrE, co-expressing Nanog and Gata4 and Neg., cells not expressing either marker]. All individual embryo data used in the preparation of this figure are contained within supplementary tables ST7.

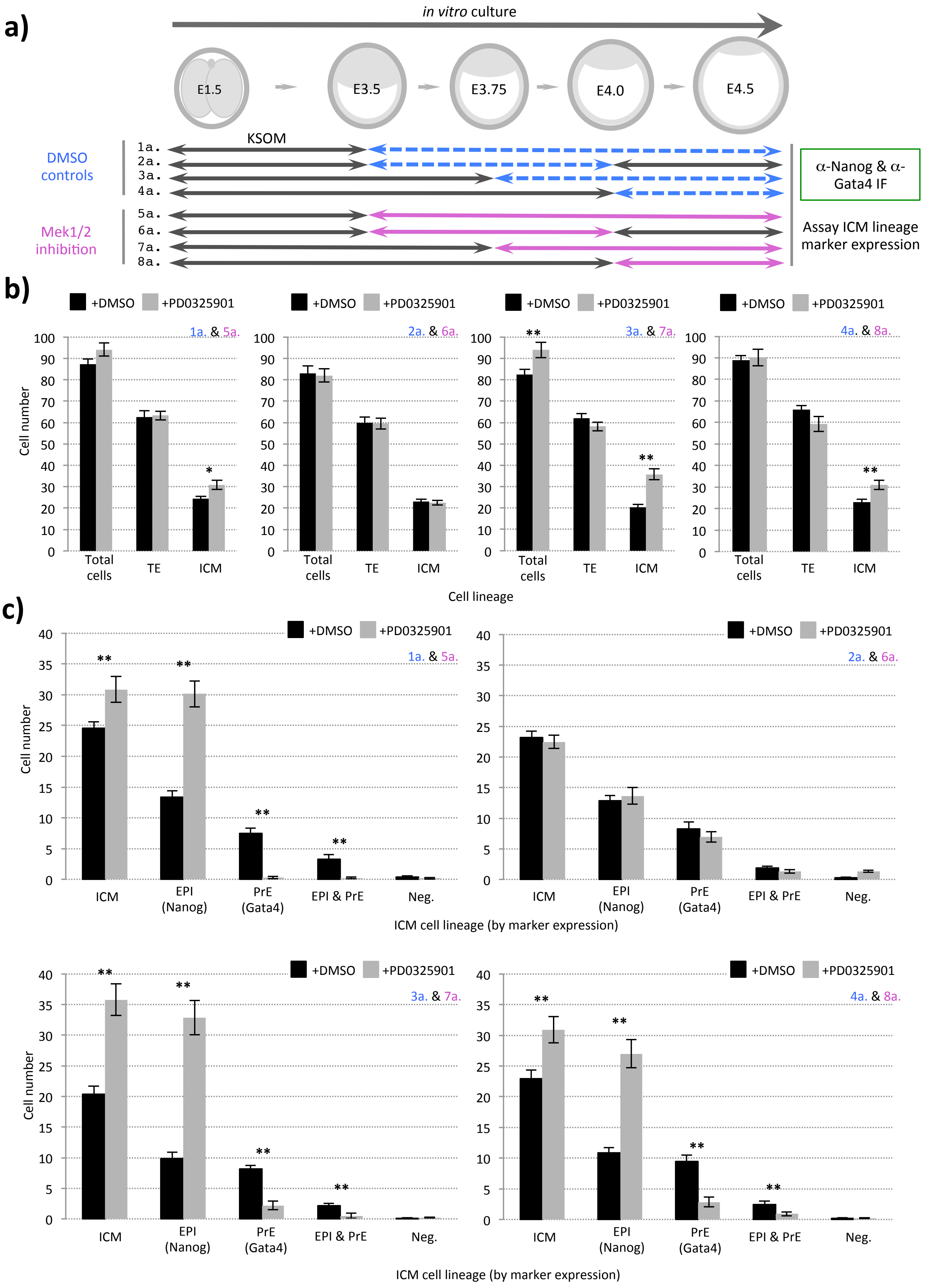

Supplementary figure S9.

Supplementary figure S9: Varied temporal inhibition of Mek1/2 activity during blastocyst maturation; averaged numbers of cells contributing to different blastocyst lineages (data supplementary to that presented in figure 2). a) Experimental schema employed to identify the developmental time-point at which Mek1/2 is required for PrE differentiation, to compare with that for p38-Mapk14/11. 2-cell (E1.5) stage embryos were *in vitro* cultured to varied blastocyst stages (ranging from E3.5 to E4.0, as indicated) and transferred into media supplemented with either p38-Mek1/2/11 inhibitor (PD0325901) or DMSO vehicle controls (note the nomenclature of each condition, 1a, 3a and 4a for DMSO controls and 5a, 7a and 8a for Mek1/2). Embryos were then cultured to the late-blastocyst (E4.5) stage and fixed for immuno-fluorescent staining against the ICM cell lineage markers Nanog and Gata4. Note that a second embryo group transferred at the early-blastocyst (E3.5) stage was removed from vehicle control/ inhibitor treatment at the mid-blastocyst (E4.0) stage and returned to normal growth media before being similarly processed at the late-blastocyst (E4.5) stage (groups 2a and 6a). **b)** Bar charts detailing average total cell number and number of cells allocated to each blastocyst lineage (*i.e.* outer TE or inner ICM) in each of the above described control and treatment conditions [see **a)**]. Error bars represent s.e.m. and * and ** denote statistical significant differences in cell number between the vehicle control (+DMSO, black bars) and Mek1/2 inhibited (+SB220025, grey bars) embryo groups, according to 2-tailed students t-test, with $p<0.05$ and $p<0.005$ confidence intervals, respectively. Note that the most suitable control and p38-Mek1/2 inhibition data have been paired into each of the four subpanels [denoted by the same numbered and coloured nomenclature that is used in panel **a)**]. **c)** Similar bar charts to those in **b)**, but describing the average number of cells allocated to each blastocyst ICM lineage, as judged by detectable lineage marker gene expression [*i.e.* Total ICM cell number as in **a)**; EPI, Nanog alone; PrE, Gata4 alone; EPI & PrE, co-expressing Nanog and Gata4 and Neg., cells not expressing either marker]. All individual embryo data used in the preparation of this figure are contained within supplementary tables ST8.

a)

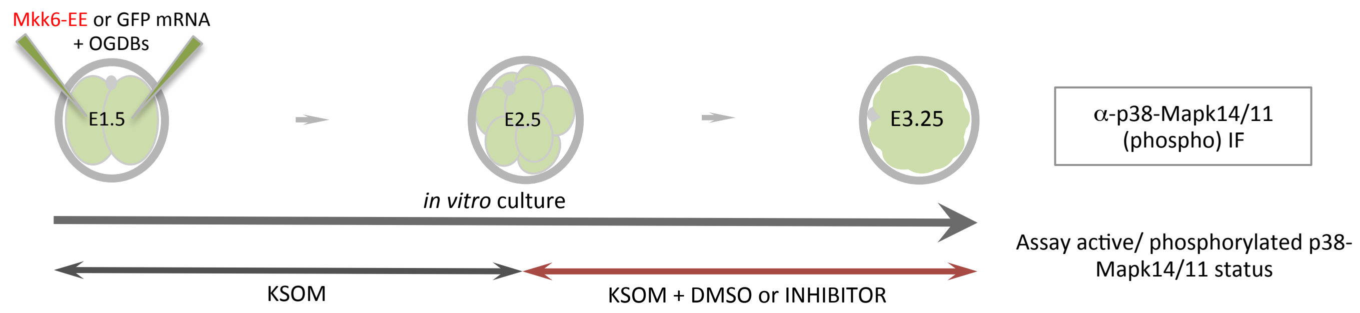

b)

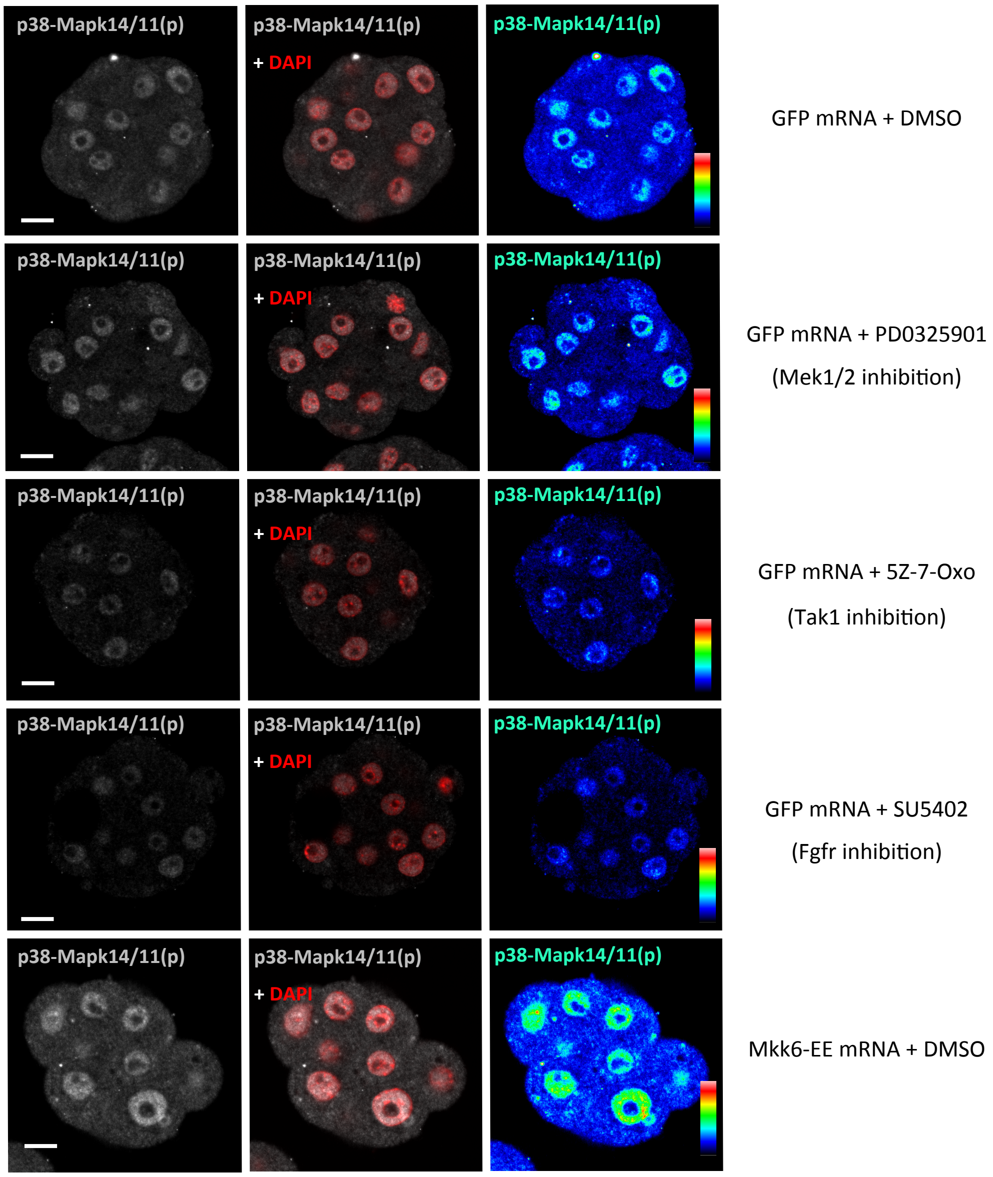

Supplementary figure S10.

Supplementary figure S10: Activated phospho-p38-Mapk14/11 [p38-Mapk14/11(p)] levels in late morula (E3.25) stage embryos, following over-expression of the constitutive active p38-Mapk14/11 targeting kinase, 'Mkk6-EE' mutant or incubation in various specific and relevant chemical inhibitors. **a)** Experimental scheme describing how 2-cell (E1.5) stage embryos were microinjected in both blastomeres with Oregon-green conjugated dextran beads (OGDBs, as an injection marker control) and either control GFP or constitutively active mutant Mkk6-EE mRNA, were *in vitro* cultured until the 8-cell (E2.5) stage. GFP microinjected embryos were then transferred into growth media supplemented with either Mek1/2 (+PD0325901), Tak1 (+5Z-7-Oxo) or Fgfr (+SU5402) specific inhibitors, or vehicle control (+DMSO), whereas Mkk6-EE microinjected embryos were only transferred into media supplemented with control DMSO. All embryo groups were then further cultured until the late morula (E3.25) stage, before being fixed and processed for immuno-fluorescent staining using an antibody specific to activated phospho-p38-Mapk14/11 [p38-Mapk14/11(p)]. Therefore the effect of the various chemical inhibitions and Mkk6-EE over-expression on activated p38-Mapk14/11(p) levels could be appropriately assayed against the same GFP microinjected and DMSO exposed control embryos. **b)** Representative single z-plane confocal section of immuno-fluorescently stained embryos [as described in **a)**]. Activated p38-Mapk14/11(p) staining shown in a spectral pixel intensity scale (on right), in grey-scale (on left) or merged with red pseudo-coloured DAPI derived DNA counterstain (central panels). Note all images of central z-plane sections were acquired using the same confocal microscopy settings. Scale bar = 15μm.

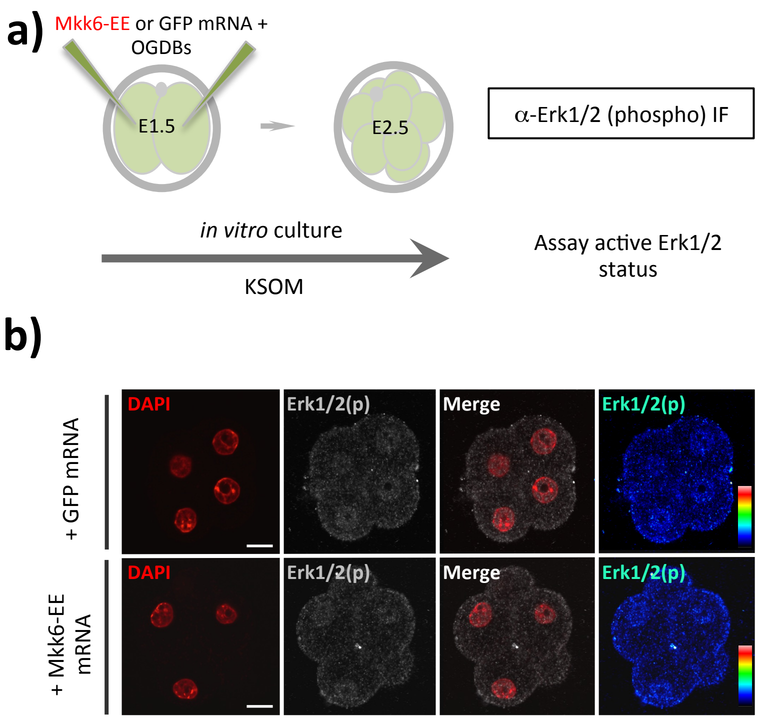

Supplementary figure S11.

Supplementary figure S11: Over-expression of the constitutively active p38-Mapk14/11 targeting kinase Mkk6-EE mutant in preimplantation stage mouse embryos does not alter activated phospho-Erk1/2 [Erk1/2(p)] levels. a) Experimental scheme describing how 2-cell (E1.5) stage embryos that were microinjected in both blastomeres with Oregon-green conjugated dextran beads (OGDBs, as an injection marker control) and either control GFP or constitutively active mutant Mkk6-EE mRNA, were *in vitro* cultured until the 8-cell (E2.5) stage, fixed and processed for immuno-fluorescent staining using an antibody specific to activated phospho-Erk1/2 [Erk1/2(p)]. Representative single z-plane confocal sections of immuno-fluorescently stained embryos [as described in **a)** and indicated]. Activated Erk1/2(p) staining shown in a spectral pixel intensity scale (far right panels), in grey-scale (panels second from left) or in grey-scale merged with red pseudo-coloured DAPI derived DNA counterstain (panels second from right). DAPI derived DNA staining alone is also shown (far left panels) Note all images of central z-plane sections were acquired using the same confocal microscopy settings. Scale bar = 15μm.

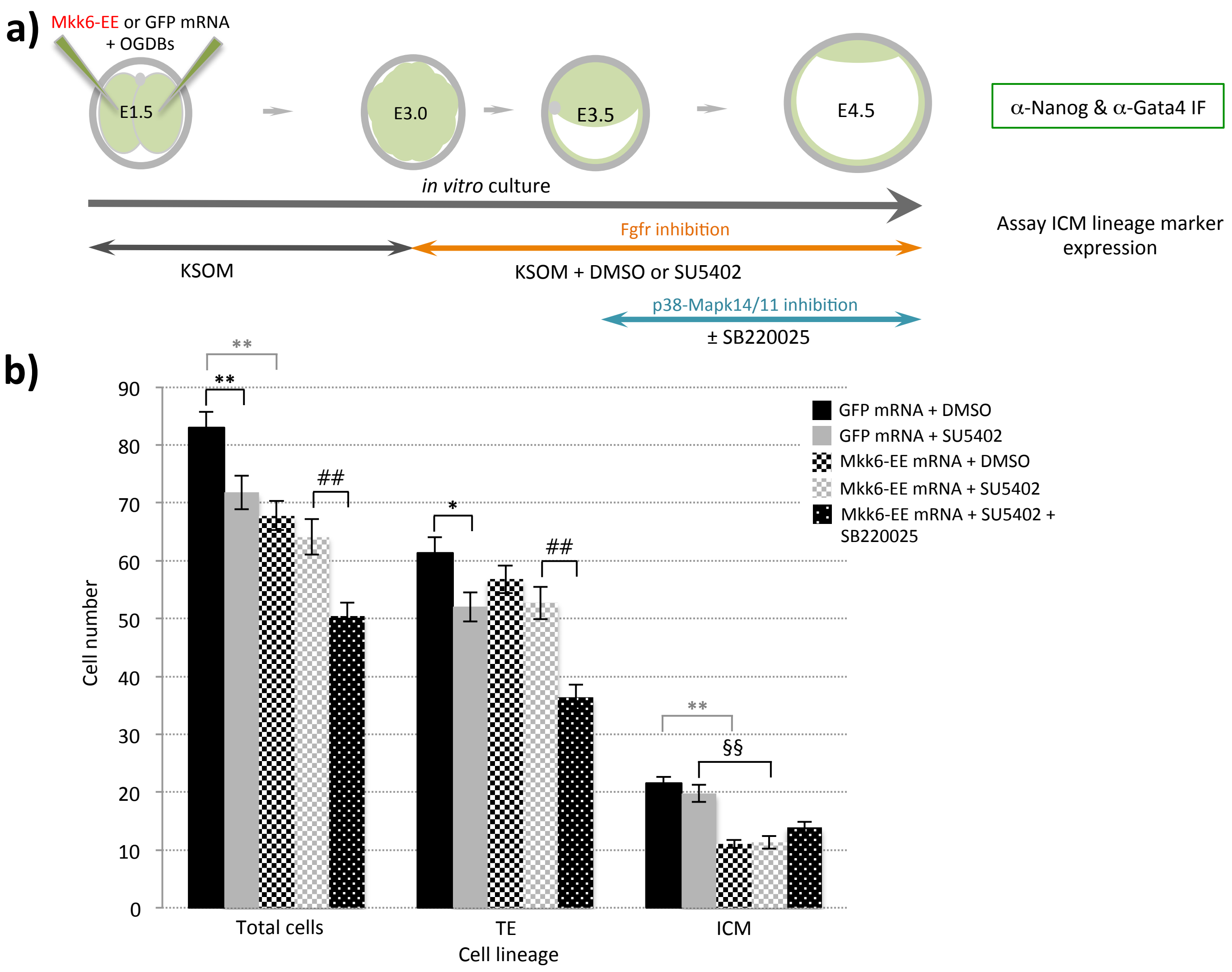

Supplementary figure S12.

Supplementary figure S12: Effect of the inhibition of Fgf-receptors (Fgfr) and the expression of Mkk6-EE rescue constructs (plus additional p38-Mapk14/11 inhibition) on total embryo, TE and ICM cell numbers (data supplementary to that presented in figure 4). a) Experimental schema detailing the regime of Fgf-receptor (Fgfr) inhibition (+SU5402), with attendant vehicle control (+DMSO) condition, from the 16-cell to late blastocyst (E3.0-E4.5) stages and optional p38-Mapk14/11 co-inhibition (±SB220025, from E3.5-E4.5), employed. Also highlighted are mRNAs microinjected (together with Oregon-green conjugated dextran beads/ OGDBs, to confirm successful mRNA delivery) into both blastomeres at the 2-cell (E1.5) stage; the constitutive active, p38-Mapk14/11 targeting kinase, 'Mkk6-EE' mutant or microinjection control 'GFP'. Immuno-fluorescence (IF) antibody details used to analyse ICM cell lineage marker protein expression in late blastocysts (E4.5) are also given. **b)** Averaged total cell number and contribution to the TE and ICM blastocyst cell lineages, based on relative spatial location and ICM marker expression status [either EPI (Nanog) or PrE (Gata4)], in each of the stated experimental conditions. Errors are represented as s.e.m. and appropriate statistically significant differences, derived from 2-tailed students t-tests, highlighted by one or two significance markers (one, representing $p<0.05$ and two denoting $p<0.005$) described thus; asterisks (*) showing differences between the 'GFP mRNA + DMSO' and 'GFP mRNA + SU5402' or 'Mkk6-EE mRNA + DMSO' (in grey) groups, the symbol § highlighting significant difference between the 'GFP mRNA + SU5402'and 'Mkk6-EE + SU5402' groups, crosses (†) between the 'Mkk6-EE + DMSO' and 'Mkk6-EE + SU5402' groups and hashtags (#) denoting divergence between the 'Mkk6-EE + SU5402' and 'Mkk6-EE + SU5402 + SB220025' groups. Data also presented in supplementary tables ST11.

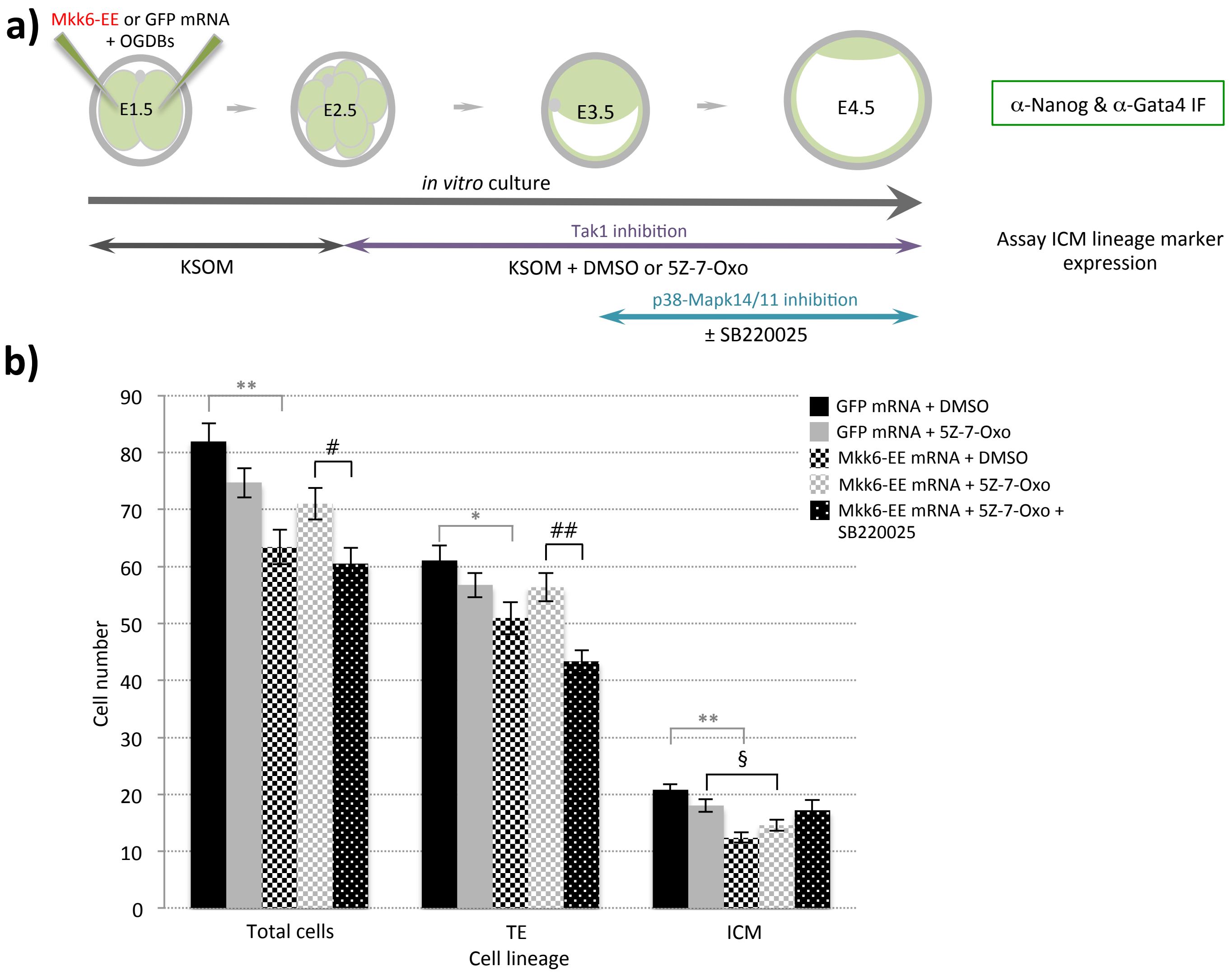

Supplementary figure S13.

Supplementary figure S13: Effect of the inhibition of Tak1 and the expression of Mkk6-EE rescue constructs (plus additional p38-Mapk14/11 inhibition) on total embryo, TE and ICM cell numbers (data supplementary to that presented in figure 5). a) Experimental schema detailing the regime of Tak1 inhibition (+5Z-7-Oxo), with attendant vehicle control (+DMSO) condition, from the 8-cell to late blastocyst (E2.5-E4.5) stages and optional p38-Mapk14/11 co-inhibition (±SB220025, from E3.5-E4.5), employed. Also highlighted are mRNAs microinjected (together with Oregon-green conjugated dextran beads/ OGDBs, to confirm successful mRNA delivery) into both blastomeres at the 2-cell (E1.5) stage; the constitutive active, p38-Mapk14/11 targeting kinase, 'Mkk6-EE' mutant or microinjection control 'GFP'. Immuno-fluorescence (IF) antibody details used to analyse ICM cell lineage marker protein expression in late blastocysts (E4.5) are also given. **b)** Averaged total cell number and contribution to the TE and ICM blastocyst cell lineages, based on relative spatial location and ICM marker expression status [either EPI (Nanog) or PrE (Gata4)], in each of the stated experimental conditions. Errors are represented as s.e.m. and appropriate statistically significant differences, derived from 2-tailed students t-tests, highlighted by one or two significance markers (one, representing $p<0.05$ and two denoting $p<0.005$) described thus; asterisks (*) showing differences between the 'GFP mRNA + DMSO' and 'GFP mRNA + 5Z-7-Oxo' or 'Mkk6-EE mRNA + DMSO' (in grey) groups, the symbol § highlighting significant difference between the 'GFP mRNA + 5Z-7-Oxo'and 'Mkk6-EE + 5Z-7-Oxo' groups, crosses (†) between the 'Mkk6-EE + DMSO' and 'Mkk6-EE + 5Z-7-Oxo' groups and hashtags (#) denoting divergence between the 'Mkk6-EE + 5Z-7-Oxo' and 'Mkk6-EE + 5Z-7-Oxo+ SB220025' groups. Data also presented in supplementary tables ST12.

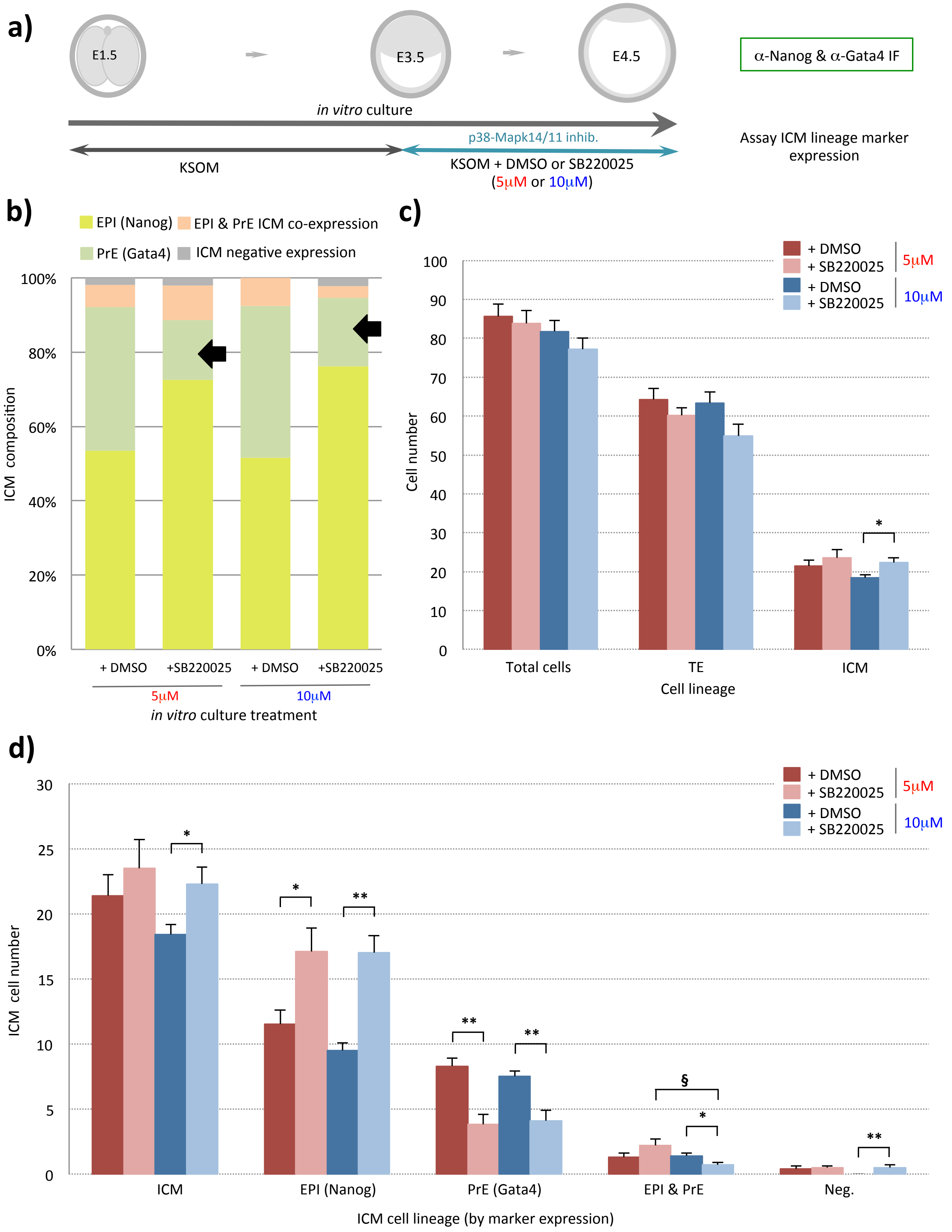

Supplementary figure S14.

Supplementary figure S14: p38-Mapk14/11 inhibition using lower concentrations of SB220025 (compared to higher/ 20μM dose used in the bulk of the study), from the early blastocyst (E3.5) to late blastocyst (E4.5) stages also impairs specified PrE cell formation in the ICM a) Experimental schema detailing the regime of p38-Mapk14/11 inhibition (+SB220025 at 5μM and 10μM concentrations), with attendant vehicle control (+DMSO) condition, from the early to late blastocyst (E3.5-E4.5) stages employed. Immuno-fluorescence (IF) antibody details used to analyse ICM cell lineage marker protein expression in late blastocysts (E4.5) are also given (*n.b.* Gata4 used as late PrE marker). **b)** Averaged percentage makeup of the ICMs of each stated condition (*i.e.* specific inhibitor concentration plus its appropriate DMSO vehicle control concentration) in relation to each specified ICM lineage; EPI or PrE (yellow and green, exclusively immuno-stained for either Nanog or Gata4, respectively), EPI & PrE co-expressing cells (orange, representing cells uncommitted to either lineage) and cells negative for either studied lineage marker (grey). The black arrows denote decreased percentage contribution of Gata4–alone positive PrE cells in the ICM of SB220025 treated embryos at both 5μM and 10μM concentrations, compared to the relevant +DMSO vehicle controls. In the +DMSO treated groups n=10 and 12 and in the +SB220025 groups n=13 and 13, for the 5μM and 10μM concentration conditions, respectively. **c)** Averaged total cell number and contribution to the TE and ICM blastocyst cell lineages, based on relative spatial location in each of the stated experimental conditions (outer; TE and inner; ICM). Errors are represented as s.e.m. and appropriate statistically significant differences, derived from 2-tailed students t-tests, highlighted by one or two significance markers (* $p<0.05$ and ** denoting $p<0.005$). **d)** Averaged contribution of cells to each ICM cell lineage, based on exclusive expression of either EPI (Nanog) or PrE (Gata4) lineage marker (or both or neither marker), in each of the stated experimental conditions. Errors and significance denoted as in **c)** – *n.b.* significant differences between the 5μM and 10μM +SB220025 conditions are denoted with the following significance marker; §. All data used to prepare this figure are described in supplementary tables ST13.

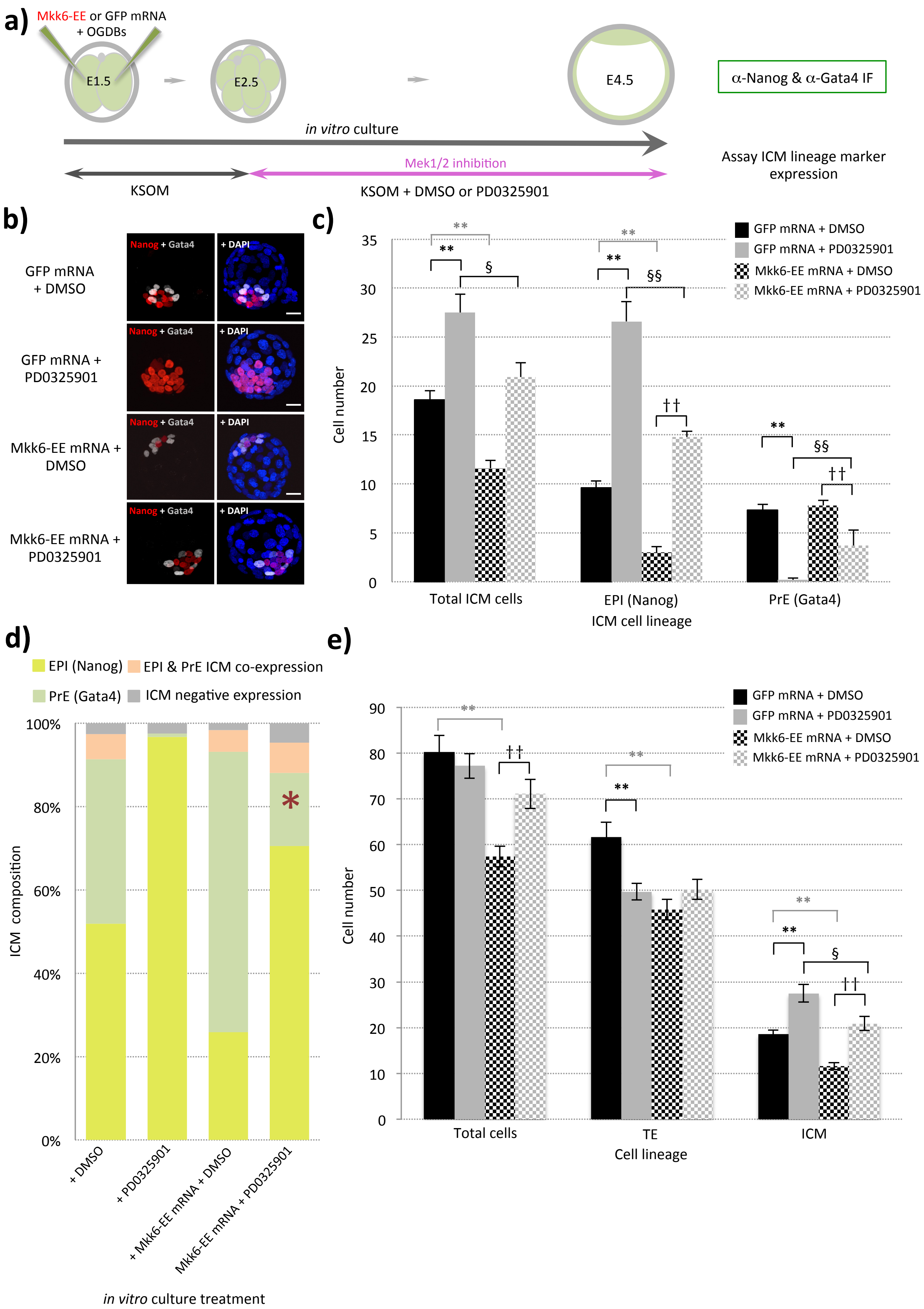

Supplementary figure S15.

Supplementary figure S15. Mek1/2 inhibition mediated block in PrE formation can be compensated by Mkk6-EE over-expression. a) Experimental schema detailing the regime of Mek1/2 inhibition (+PD0325901), with attendant vehicle control (+DMSO) condition, from the 8-cell to late blastocyst (E2.5-E4.5) stages employed. Also highlighted are mRNAs microinjected (together with Oregon-green conjugated dextran beads/ OGDBs, to confirm successful mRNA delivery) into both blastomeres at the 2-cell (E1.5) stage; the constitutive active, p38-Mapk14/11 targeting kinase, 'Mkk6-EE' mutant or microinjection control 'GFP'. Immuno-fluorescence (IF) antibody details used to analyse ICM cell lineage marker protein expression in late blastocysts (E4.5) are also given. **b)** Representative confocal z-plane projections of ICM lineage marker expression (Nanog, for EPI, in red and Gata4, for PrE, in grey-scale, plus DAPI DNA counter-stain in blue) in each of the studied conditions in late-blastocyst (E4.5) stage embryos; GFP microinjection control plus DMSO vehicle control (GFP mRNA + DMSO; n=21), GFP microinjection control plus Mek1/2 inhibition (GFP mRNA + PD0325901; n=23), Mkk6-EE microinjection plus DMSO vehicle control (Mkk6-EE mRNA +DMSO; n=20) and Mkk6-EE microinjection plus Mek1/2 inhibition (Mkk6-EE mRNA + PD0325901; n=25). Scale bar = 15μm. **c)** Averaged contribution of cells to each ICM cell lineage, based on exclusive expression of either EPI (Nanog) or PrE (Gata4) lineage marker, in each of the stated experimental conditions Errors are represented as s.e.m. and appropriate statistically significant differences, derived from 2-tailed students t-tests, highlighted by one or two significance markers (one, representing $p<0.05$ and two denoting $p<0.005$) described thus; asterisks (*) showing differences between the 'GFP mRNA + DMSO' and 'GFP mRNA + PD0325901'or 'Mkk6-EE mRNA + DMSO' (in grey) groups, the symbol § highlighting significant difference between the 'GFP mRNA + PD0325901' and 'Mkk6-EE + PD0325901'groups and crosses (†) between the 'Mkk6-EE + DMSO' and 'Mkk6-EE + PD0325901' groups. **d)** Averaged percentage makeup of the ICMs of each stated condition in relation to each specified ICM lineage; EPI or PrE (yellow and green, exclusively immuno-stained for either Nanog or Gata4, respectively), EPI & PrE co-expressing cells (orange, representing cells uncommitted to either lineage) and cells negative for either studied lineage marker (grey). Maroon asterisk denotes the rescue of the PrE component of ICM cells in Mek1/2 inhibited cells expressing the p38-Mapk14/11 activating kinase mutant, Mkk6-EE ('Mkk6-EE mRNA + PD0325901' group) compared to the appropriate Mek1/2 inhibited condition (the 'GFP mRNA + PD0325901' group). **e)** Averaged total cell number and contribution to the TE and ICM blastocyst cell lineages, based on relative spatial location (outer; TE and inner; ICM) and ICM marker expression status [either EPI (Nanog) or PrE (Gata4)], in each of the stated experimental conditions. The errors and statistically significance markers are as described for **c)**. All data used to prepare this figure are described in supplementary tables ST14.

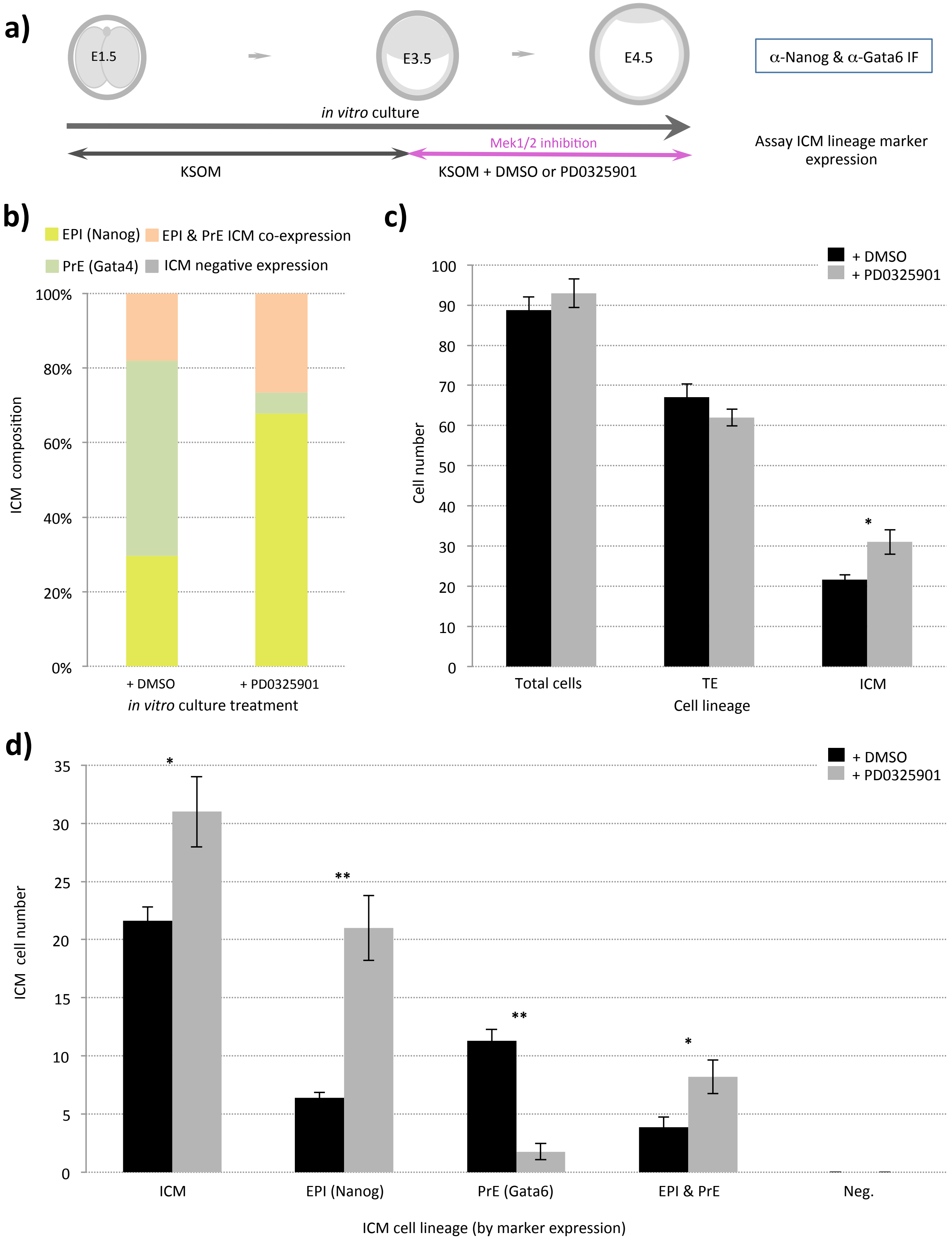

Supplementary figure S16.

Supplementary figure S16. Mek1/2 inhibition from the early blastocyst (E3.5) to late blastocyst (E4.5) stages causes respective reductions and increases in PrE and EPI specified cells but also increases the number of uncommitted ICM cells. a) Experimental schema detailing the regime of Mek1/2 inhibition (+PD0325901), with attendant vehicle control (+DMSO) condition, from the early to late blastocyst (E3.5-E4.5) stages employed. Immuno-fluorescence (IF) antibody details used to analyse ICM cell lineage marker protein expression in late blastocysts (E4.5) are also given (*n.b.* Gata6 used as PrE marker). **b)** Averaged percentage makeup of the ICMs of each stated condition in relation to each specified ICM lineage; EPI or PrE (yellow and green, exclusively immuno-stained for either Nanog or Gata6, respectively), EPI & PrE co-expressing cells (orange, representing cells uncommitted to either lineage) and cells negative for either studied lineage marker (grey). In the +DMSO treated group n=10 and in the +PD0325901 group n=9. **c)** Averaged total cell number and contribution to the TE and ICM blastocyst cell lineages, based on relative spatial location (outer; TE and inner; ICM) in each of the stated experimental conditions. Errors are represented as s.e.m. and appropriate statistically significant differences, derived from 2-tailed students t-tests, highlighted by one or two significance markers (* $p<0.05$ and ** denoting $p<0.005$). **d)** Averaged contribution of cells to each ICM cell lineage, based on exclusive expression of either EPI (Nanog) or PrE (Gata6) lineage markers (or both or neither marker), in each of the stated experimental conditions. Errors and significance denoted as in **c)**. All data used to prepare this figure are described in supplementary tables ST15.

Supplementary tables ST1 (a&b)

+DMSO			
#	TOTAL NUMBER OF CELLS		
	EMBRYO	TE	ICM TOTAL
1	89	66	23
2	59	41	18
3	89	68	21
4	67	47	20
5	90	66	24
6	64	49	15
7	70	50	20
8	104	88	16
9	80	59	21
10	75	51	24
11	79	62	17
12	79	49	30
13	73	54	19
14	87	61	26
15	94	78	16
16	93	75	18
17	42	33	9
18	101	83	18
19	82	66	16
20	110	87	23
21	73	52	21
22	82	70	12
23	101	78	23
24	100	85	15
25	99	80	19
26	80	65	15
27	79	65	14
28	97	70	27
29	103	83	20
30	75	62	13
31	81	62	19
32	103	78	25
33	90	67	23
34	101	85	16
35	99	75	24
36	103	76	27
37	93	77	16
38	85	63	22
39	76	52	24
40	99	79	20
41	92	68	24
42			
43			
44			
45			
TOTAL	3538	2725	813
**AVERAGE**	**86.3**	**66.5**	**19.8**
SEM	2.2	2.1	0.7

Supplementary table ST1a

+p38-Mapk14/11inhibitor (SB220025)			
#	TOTAL NUMBER OF CELLS		
	EMBRYO	TE	ICM TOTAL
1	20	14	6
2	35	25	10
3	22	12	10
4	31	17	14
5	26	14	12
6	43	25	18
7	19	13	6
8	23	12	11
9	15	11	4
10	27	19	8
11	32	22	10
12	23	17	6
13	27	20	7
14	33	23	14
15	13	9	4
16	40	22	20
17	30	20	10
18	42	25	17
19	18	13	5
20	26	17	10
21	31	22	9
22	27	21	7
23	33	24	9
24	50	30	20
25	19	13	6
26	21	13	8
27	53	43	10
28	26	15	11
29	53	41	12
30	16	13	3
31	15	10	5
32	33	21	12
33	43	31	12
34	26	18	8
35	36	25	11
36	26	17	9
37	40	27	13
38	54	42	12
39	31	18	13
40	44	27	17
41	19	11	8
42	56	42	14
43	28	14	14
44	21	15	6
45	25	18	7
TOTAL	1371	921	458
**AVERAGE**	**30.5**	**20.5**	**10.2**
**SEM**	1.7	1.3	0.6
Stat. sig. (exp. vs. con embryo; ST1a) *p<0.05, **p<0.005	**	**	**
p-value (2-tailed students t-test)	7.82E-34	4.08E-32	1.70E-16

Supplementary table ST1b

Supplementary tables ST1 (a & b): Individual embryo data used to generate averaged data presented in supplementary figure S1 b; *the average number of outer and inner cells in embryos in vitro cultured in the presence of vehicle control +(DMSO) or p38-Mapk14/11 inhibitor (+SB220025) from the 8-cell (E2.5) to late blastocyst (E4.5) stages (averaged from three-data sets of embryos immuno-fluorescently stained for Nanog in combination with either Cdx2, Sox17 or Gata6).*

Supplementary tables ST2 (a-f)

+DMSO (IF: Nanog/ Cdx2)							
	TOTAL NUMBER OF CELLS						
					ICM		
#	EMBRYO	TE (Cdx2 +ve)	Cdx2 +ve Inner cells	Nanog +ve outer cells	EPI (Nanog +ve)	PrE (Nanog -ve)	TOTAL
1	97	70	0	11	15	12	27
2	103	83	0	30	13	7	20
3	75	62	0	20	6	7	13
4	81	62	0	3	10	9	19
5	103	78	0	10	17	8	25
6	90	67	0	8	15	8	23
7	101	85	0	12	9	7	16
8	99	75	0	25	12	12	24
9	103	76	0	7	14	13	27
10	93	77	0	18	8	8	16
11	85	63	0	12	12	10	22
12	76	52	0	9	16	8	24
13	99	79	0	9	7	13	20
14	92	68	0	2	11	13	24
15							
16							
17							
18							
**TOTAL**	1297	997	0	176	165	135	300
**AVERAGE**	**92.6**	**71.2**	**0.0**	**12.6**	**11.8**	**9.6**	**21.4**
**SEM**	2.7	2.5	0.0	2.1	0.9	0.7	1.1

Supplementary table ST2a

+p38-Mapk14/11inhibitor (SB220025, IF: Nanog/ Cdx2)							
	TOTAL NUMBER OF CELLS						
					ICM		
#	EMBRYO	TE (Cdx2 +ve)	Cdx2 +ve Inner cells	Nanog +ve outer cells	EPI (Nanog +ve)	PrE (Nanog -ve)	TOTAL
1	26	15	8	15	11	0	11
2	53	41	3	41	12	0	12
3	16	13	0	13	3	0	3
4	15	10	2	10	5	0	5
5	33	21	0	21	12	0	12
6	43	31	1	31	12	0	12
7	26	18	6	18	8	0	8
8	36	25	0	25	11	0	11
9	26	17	2	17	9	0	9
10	40	27	0	25	12	0	13
11	54	42	4	16	12	0	12
12	31	18	5	17	13	0	13
13	44	27	3	16	17	0	17
14	19	11	4	11	8	0	8
15	56	42	1	13	14	0	14
16	28	14	0	7	14	0	14
17	21	15	3	15	6	0	6
18	25	18	1	14	7	0	7
**TOTAL**	592	405	43	325	186	0	187
**AVERAGE**	**32.9**	**22.5**	**2.4**	**18.1**	**10.4**	**0.0**	**10.4**
**SEM**	3.0	2.5	0.5	1.9	0.8	0.0	0.9
Stat. sig. (exp. vs. con embryo; ST2a) *p<0.05, **p<0.005	**	**	**			**	**
p-value (2-tailed students t-test)	6.16E-15	2.20E-14	6.21E-04	6.70E-02	2.56E-01	7.46E-17	7.83E-09

Supplementary table ST2b

+DMSO (IF: Nanog/ Sox17)								
	TOTAL NUMBER OF CELLS							
				ICM				
#	EMBRYO	TE	Nanog +ve TE cells	Nanog	Sox17	Nanog/Sox17 (co-expressed)	Nanog/Sox17 -ve	TOTAL
1	87	61	17	18	5	3	0	26
2	94	78	32	10	4	2	0	16
3	93	75	10	7	10	1	0	18
4	42	33	17	6	2	1	0	9
5	101	83	20	6	9	3	0	18
6	82	66	14	8	3	4	1	16
7	110	87	10	13	9	1	0	23
8	73	52	11	13	4	3	1	21
9	82	70	16	8	4	0	0	12
10	101	78	7	11	8	2	2	23
11	100	85	14	8	7	0	0	15
12	99	80	5	10	7	2	0	19
13	80	65	12	5	7	3	0	15
14	79	65	28	11	2	1	0	14
15								
TOTAL	1223	978	213	134	81	26	4	245
**AVERAGE**	**87.4**	**69.9**	**15.2**	**9.6**	**5.8**	**1.9**	**0.3**	**17.5**
SEM	4.5	3.9	2.0	0.9	0.7	0.3	0.2	1.2

Supplementary table ST2c

+p38-Mapk14/11 inhibitor (SB220025, IF: Nanog/ Sox17)								
	TOTAL NUMBER OF CELLS							
#	EMBRYO	TE	Nanog +ve TE cells	ICM				
				Nanog	Sox17	Nanog/Sox17 (co expressed)	Nanog/Sox17 -ve	TOTAL
1	27	20	16	7	0	0	0	7
2	33	23	12	13	0	0	1	14
3	13	9	4	4	0	0	0	4
4	40	22	22	18	0	0	2	20
5	30	20	19	10	0	0	0	10
6	42	25	18	17	0	0	0	17
7	18	13	13	5	0	0	0	5
8	26	17	13	9	0	0	1	10
9	31	22	22	9	0	0	0	9
10	27	21	13	6	0	0	1	7
11	33	24	12	9	0	0	0	9
12	50	30	17	20	0	0	0	20
13	19	13	13	6	0	0	0	6
14	21	13	10	8	0	0	0	8
15	53	43	35	10	0	0	0	10
TOTAL	463	315	239	151	0	0	5	156
**AVERAGE**	**30.9**	**21.0**	**15.9**	**10.1**	**0.0**	**0.0**	**0.3**	**10.4**
SEM	3.0	2.1	1.8	1.3	0.0	0.0	0.2	1.3
Stat. sig. (exp. vs. con embryos; ST2c) *p<0.05, **p<0.005	**	**			**	**		**
p-value (2-tailed students t-test)	3.99E-11	1.20E-11	7.92E-01	7.57E-01	6.15E-09	3.16E-06	8.36E-01	5.54E-04

Supplementary table ST2d

+DMSO (IF: Nanog/ Gata6)								
#	TOTAL NUMBER OF CELLS							
	EMBRYO	TE	Nanog +ve TE cells	ICM				
				Nanog	Gata6	Nanog/Gata6 (co-expressed)	Nanog/Gata6 -ve	TOTAL
1	89	66	0	9	13	1	0	23
2	59	41	6	9	5	4	0	18
3	89	68	0	6	7	8	0	21
4	67	47	1	6	6	8	0	20
5	90	66	0	10	5	9	0	24
6	64	49	0	11	1	3	0	15
7	70	50	0	8	8	4	0	20
8	104	88	1	6	8	2	0	16
9	80	59	0	8	11	2	0	21
10	75	51	0	8	6	10	0	24
11	79	62	0	5	9	3	0	17
12	79	49	0	11	17	2	0	30
13	73	54	0	5	3	11	0	19
**TOTAL**	1018	750	8	102	99	67	0	268
**AVERAGE**	**78.3**	**57.7**	**0.6**	**7.8**	**7.6**	**5.2**	**0.0**	**20.6**
**SEM**	3.4	3.4	0.5	0.6	1.2	1.0	0.0	1.1

Supplementary table ST2e

p38-Mapk14/11 inhibitor (SB220025, IF: Nanog/ Gata6)								
	TOTAL NUMBER OF CELLS							
				ICM				
#	EMBRYO	TE	Nanog +ve TE cells	Nanog	Gata6	Nanog/Gata6 (co-expressed)	Nanog/Gata6 -ve	TOTAL
1	20	14	4	3	0	3	0	6
2	35	25	3	7	0	3	0	10
3	22	12	0	1	0	9	0	10
4	31	17	0	0	1	13	0	14
5	26	14	0	3	0	9	0	12
6	43	25	0	1	2	15	0	18
7	19	13	2	1	1	4	0	6
8	23	12	0	0	0	11	0	11
9	15	11	0	0	0	4	0	4
10	27	19	2	1	0	0	7	8
11	32	22	0	0	0	10	0	10
12	23	17	0	0	0	6	0	6
13								
**TOTAL**	316	201	11	17	4	87	7	115
**AVERAGE**	**26.3**	**16.8**	**0.9**	**1.4**	**0.3**	**7.3**	**0.6**	**9.6**
**SEM**	2.3	1.4	0.4	0.6	0.2	1.3	0.6	1.1
Stat. sig. (exp. vs. con embryo; ST2e) *p<0.05, **p<0.005	**	**		**	**			**
p-value (2-tailed students t-test)	1.11E-11	2.30E-10	6.34E-01	8.59E-08	5.35E-06	2.10E-01	3.08E-01	4.62E-07

Supplementary table ST2f

Supplementary tables ST2 (a - f): Individual embryo data used to generate averaged data presented in supplementary figure S1 d, d', e & e'; *the average number of cells contributing to ICM cell lineages in embryos in vitro cultured in the presence of vehicle control (DMSO) or p38-Mapk14/11 inhibitor (SB220025) from the 8-cell (E2.5) to late blastocyst (E4.5) stages in embryos immuno-fluorescently stained for Nanog in combination with either Cdx2 (a & b), Sox17 (c & d) or Gata6 (e & f).*

Supplementary tables ST3 (a-f)

+DMSO (IF: Nanog/ Gata4)								
	TOTAL NUMBER OF CELLS							
				ICM				
#	EMBRYO	TE	Nanog +ve TE cells	Nanog	Gata4	Nanog/Gata4 (co-expressed)	Nanog/Gata4 -ve	TOTAL
1	83	61	6	10	12	0	0	22
2	101	67	0	17	7	10	0	34
3	93	70	12	8	10	5	0	23
4	75	50	11	10	8	6	1	25
5	78	55	5	10	13	0	0	23
6	82	58	12	9	12	3	0	24
7	87	65	13	7	14	0	1	22
8	105	85	0	7	10	2	1	20
9	99	79	0	8	11	0	1	20
10	91	72	0	7	7	5	0	19
11	85	69	0	10	6	0	0	16
12	78	54	2	18	6	0	0	24
13	112	95	8	8	6	3	0	17
14	113	92	10	5	14	1	1	21
15	74	53	0	14	4	2	1	21
16	92	74	17	9	6	3	0	18
17	83	60	0	9	12	1	1	23
18	82	56	0	13	8	3	2	26
19	106	80	2	12	11	3	0	26
20	56	37	6	6	9	2	2	19
21	84	58	7	15	8	3	0	26
22	99	76	20	10	7	3	3	23
23	83	57	3	12	12	2	0	26
24	90	64	7	17	8	0	1	26
25	109	82	0	15	11	0	1	27
26	103	83	0	12	6	2	0	20
27	70	54	19	9	6	1	0	16
28								
29								
30								
31								
32								
33								
TOTAL	2413	1806	160	287	244	60	16	607
AVERAGE	**89.4**	**66.9**	**5.9**	**10.6**	**9.0**	**2.2**	**0.6**	**22.5**
SEM	2.7	2.7	1.2	0.7	0.5	0.4	0.2	0.8

Supplementary table ST3a

+p38-Mapk14/11 inhibitor (SB220025, IF: Nanog/ Gata4)								
	TOTAL NUMBER OF CELLS							
				ICM				
#	EMBRYO	TE	Nanog +ve TE cells	Nanog	Gata4	Nanog/Gata4 (co-expressed)	Nanog/Gata4 -ve	TOTAL
1	66	55	19	6	2	1	2	11
2	75	52	11	17	4	2	0	23
3	65	49	25	15	0	0	1	16
4	73	50	30	17	4	2	0	23
5	105	82	20	16	6	0	1	23
6	59	43	19	15	0	0	1	16
7	51	38	10	8	4	1	0	13
8	69	56	26	13	0	0	0	13
9	41	31	14	9	0	0	1	10
10	84	62	24	15	2	5	0	22
11	91	73	15	8	6	2	2	18
12	64	51	26	11	0	2	0	13
13	71	54	12	8	7	1	1	17
14	117	98	8	13	5	0	1	19
15	67	55	8	6	0	6	0	12
16	107	82	14	16	7	2	0	25
17	80	60	20	13	3	3	1	20
18	117	97	0	8	10	2	0	20
19	73	55	10	7	4	5	2	18
20	78	49	19	25	2	0	2	29
21	87	63	48	24	0	0	0	24
22	95	71	5	14	7	2	1	24
23	52	42	11	10	0	0	0	10
24	92	82	35	9	0	0	1	10
25	72	50	31	15	3	3	1	22
26	85	68	35	14	2	0	1	17
27	64	47	19	15	0	2	0	17
28	82	65	25	12	4	1	0	17
29	83	62	26	21	0	0	0	21
30	79	59	42	20	0	0	0	20
31	72	46	23	22	2	2	0	26
32	91	72	28	13	3	1	2	19
33	56	38	28	16	0	0	2	18
**TOTAL**	2563	1957	686	451	87	45	23	606
**AVERAGE**	**77.7**	**59.3**	**20.8**	**13.7**	**2.6**	**1.4**	**0.7**	**18.4**
**SEM**	3.1	2.8	1.9	0.9	0.5	0.3	0.1	0.9
Stat. sig. (exp. vs. con. embryos; ST3a) *p<0.05, **p<0.005	*		**	*	**			**
p-value (2-tailed students t-test)	7.43E-03	5.88E-02	3.65E-08	1.02E-02	2.54E-12	9.64E-02	6.09E-01	9.52E-04

Supplementary table ST3b

+DMSO (IF: Nanog/ Sox17)								
	TOTAL NUMBER OF CELLS							
				ICM				
#	EMBRYO	TE	Nanog +ve TE cells	Nanog	Sox17	Nanog/Sox17 (co-expressed)	Nanog/Sox17 -ve	TOTAL
1	100	78	22	12	6	3	1	22
2	85	63	12	8	6	8	0	22
3	88	64	4	15	5	4	0	24
4	74	56	6	8	3	7	0	18
5	67	46	4	13	2	6	0	21
6	87	66	22	9	8	3	1	21
7	88	67	7	11	7	2	1	21
8	82	62	13	10	5	4	1	20
9	80	53	15	14	6	6	1	27
10	75	48	9	14	8	5	0	27
11	89	61	7	10	10	8	0	28
12	92	70	16	11	8	3	0	22
13	112	90	8	9	12	1	0	22
14	85	68	14	6	11	0	0	17
15	96	77	6	8	11	0	0	19
16	92	73	13	7	10	1	1	19
17	76	56	6	11	5	2	2	20
18	100	83	12	6	11	0	0	17
19								
20								
TOTAL	1568	1181	196	182	134	63	8	387
**AVERAGE**	**87.1**	**65.6**	**10.9**	**10.1**	**7.4**	**3.5**	**0.4**	**21.5**
SEM	2.6	2.8	1.3	0.6	0.7	0.6	0.1	0.8

Supplementary table ST3c

+p38-Mapk14/11 inhibitor (SB220025, IF: Nanog/ Sox17)								
	TOTAL NUMBER OF CELLS							
				ICM				
#	EMBRYO	TE	Nanog +ve TE cells	Nanog	Sox17	Nanog/Sox17 (co-expressed)	Nanog/Sox17 -ve	TOTAL
1	71	42	19	25	0	2	2	29
2	76	52	21	17	0	5	2	24
3	77	55	29	17	0	3	2	22
4	75	54	26	21	0	0	0	21
5	77	55	48	22	0	0	0	22
6	92	70	35	14	2	6	0	22
7	73	56	33	16	0	1	0	17
8	78	62	19	8	3	5	0	16
9	84	58	25	16	3	7	0	26
10	81	63	20	9	8	0	1	18
11	72	55	27	16	1	0	0	17
12	92	68	30	15	9	0	0	24
13	76	60	23	11	1	1	3	16
14	73	57	12	9	2	5	0	16
15	91	66	26	16	4	5	0	25
16	78	63	35	12	0	3	0	15
17	78	69	22	5	0	4	0	9
18	85	64	17	12	2	5	2	21
19	77	65	24	8	4	0	0	12
20	72	57	32	7	3	3	2	15
TOTAL	1578	1191	523	276	42	55	14	387
**AVERAGE**	**78.9**	**59.6**	**26.2**	**13.8**	**2.1**	**2.8**	**0.7**	**19.4**
SEM	1.5	1.5	1.8	1.2	0.6	0.5	0.2	1.1
Stat. sig. (exp. vs. con. embryos; ST3c) *p<0.05, **p<0.005	*		**	*	**			
p-value (2-tailed students t-test)	7.33E-03	5.63E-02	6.96E-08	1.21E-02	8.40E-07	3.67E-01	3.67E-01	1.32E-01

Supplementary table ST3d

+DMSO (IF: Nanog/ Gata6)								
#	TOTAL NUMBER OF CELLS							
				ICM				
	EMBRYO	TE	Nanog +ve TE cells	Nanog	Gata6	Nanog/Gata6 co-expressed)	Nanog/Gata6 -ve	TOTAL
1	107	78	17	14	7	8	0	29
2	103	73	6	10	9	11	0	30
3	92	73	14	1	7	11	0	19
4	80	56	24	8	7	9	0	24
5	90	67	14	9	7	7	0	23
6	75	46	4	9	10	10	0	29
7	94	75	5	7	6	6	0	19
8	92	69	7	10	5	8	0	23
9	86	63	1	11	9	3	0	23
10	77	52	4	6	9	10	0	25
11	86	63	13	12	6	5	0	23
12	103	81	2	5	14	3	0	22
13	75	56	4	10	5	4	0	19
14	86	68	10	8	8	2	0	18
15	97	76	23	7	7	7	0	21
16	81	63	25	8	5	5	0	18
17	87	66	15	6	8	7	0	21
18	91	68	19	6	16	1	0	23
19	93	63	3	12	13	5	0	30
20	99	71	22	11	9	8	0	28
21	94	69	5	9	10	6	0	25
22	95	68	3	11	13	3	0	27
23	87	66	20	7	9	5	0	21
24	88	63	10	15	6	4	0	25
25								
26								
27								
TOTAL	2158	1593	270	85	76	83	0	244
**AVERAGE**	**89.9**	**66.4**	**11.3**	**8.8**	**8.5**	**6.2**	**0.0**	**23.5**
SEM	1.8	1.7	1.6	0.6	0.6	0.6	0.0	1.2

Supplementary table ST3e

+p38-Mapk14/11 inhibitor (SB220025, IF: Nanog/ Gata6)								
	TOTAL NUMBER OF CELLS							
				ICM				
#	EMBRYO	TE	Nanog +ve TE cells	Nanog	Gata6	Nanog/Gata6 co-expressed)	Nanog/Gata6 -ve	TOTAL
1	91	66	47	9	4	12	0	25
2	65	50	26	4	3	8	0	15
3	90	65	52	9	3	13	0	25
4	79	59	42	6	1	13	0	20
5	88	67	35	5	3	13	0	21
6	91	75	18	5	4	7	0	16
7	87	67	45	6	5	9	0	20
8	80	67	43	0	2	11	0	13
9	65	42	36	0	0	23	0	23
10	81	56	34	10	1	14	0	25
11	80	59	29	7	4	10	0	21
12	65	55	19	3	3	4	0	10
13	73	60	35	8	1	4	0	13
14	48	38	21	4	1	5	0	10
15	61	47	24	5	2	7	0	14
16	88	63	25	13	4	8	0	25
17	79	60	12	8	1	10	0	19
18	75	62	36	6	3	4	0	13
19	84	63	39	5	3	13	0	21
20	73	55	23	6	2	10	0	18
21	100	80	44	8	0	12	0	20
22	88	71	40	6	2	9	0	17
23	82	62	29	6	4	10	0	20
24	83	67	26	8	5	3	0	16
25	91	75	68	4	2	10	0	16
26	87	68	28	9	5	5	0	19
27	82	62	19	9	1	10	0	20
**TOTAL**	2156	1661	895	169	69	257	0	495
**AVERAGE**	**79.9**	**61.5**	**33.1**	**6.3**	**2.6**	**9.5**	**0.0**	**18.3**
**SEM**	2.2	1.9	2.4	0.6	0.3	0.8	0.0	0.9
Stat. sig. (exp. vs. con. embryos; ST3e) *p<0.05, **p<0.005	**		**	**	**	**		**
p-value (2-tailed students t-test)	9.27E-04	5.95E-02	1.28E-09	3.18E-03	2.21E-12	1.83E-03	n/a	4.27E-05

Supplementary table ST3f

Supplementary tables ST3 (a - f): Individual embryo data used to generate averaged data presented in figure 1 c & d; *the average number of cells contributing to all blastocyst cell lineages in embryos in vitro cultured in the presence of vehicle control (DMSO) or p38-Mapk14/11 inhibitor (SB220025) from the early (E3.5) to late blastocyst (E4.5) stages in embryos immuno-fluorescently stained for Nanog in combination with either Gata4 (a & b), Sox17 (c & d) or Gata6 (e & f).*

Supplementary tables ST4 (a&b)

+DMSO (IF: Nanog/ Cdx2)								
	TOTAL NUMBER OF CELLS							
				ICM				
#	EMBRYO	TE (Cdx2 +ve)	Nanog +ve TE cells	Nanog	Nanog/ Cdx2 -ve (PrE)	Nanog/Cdx2 (co-expressed)	Cdx2 +ve	TOTAL
1	79	63	35	11	5	0	0	16
2	109	75	15	20	13	0	1	34
3	92	69	12	15	7	0	1	23
4	56	42	6	10	3	0	1	14
5	84	62	9	15	7	0	0	22
6	94	70	5	13	11	0	0	24
7	76	58	10	12	6	0	0	18
8	61	40	7	12	9	0	0	21
9	80	70	8	6	4	0	0	10
10	92	73	30	10	9	0	0	19
11	90	66	15	16	8	0	0	24
12	100	77	21	13	10	0	0	23
13	90	63	5	13	14	0	0	27
14	54	48	8	4	2	0	0	6
15	60	45	1	11	3	0	1	15
16	90	70	10	6	14	0	0	20
17	83	61	11	14	8	0	0	22
18								
19								
20								
21								
22								
23								
24								
25								
26								
TOTAL	1390	1052	208	201	133	0		338
AVERAGE	**81.8**	**61.9**	**12.2**	**11.8**	**7.8**	**0.0**		**19.9**
SEM	3.8	2.8	2.2	1.0	0.9	0.0		1.6

Supplementary table ST4a

+p38-Mapk14/11 inhibitor (SB220025, IF: Nanog/ Cdx2)								
	TOTAL NUMBER OF CELLS							
				ICM				
#	EMBRYO	TE (Cdx2 +ve)	Nanog +ve TE cells	Nanog	Nanog/ Cdx2 -ve (PrE)	Nanog/Cdx2 (co-expressed)	Cdx2 +ve	TOTAL
1	80	67	28	11	2	0	0	13
2	89	67	50	18	0	3	1	22
3	71	57	25	11	0	2	1	14
4	73	60	38	11	0	0	2	13
5	64	50	34	10	1	3	0	14
6	91	66	26	22	1	1	1	25
7	71	48	23	18	3	0	2	23
8	71	52	25	15	2	1	1	19
9	74	46	25	20	2	2	4	28
10	75	58	30	14	2	1	0	17
11	77	64	8	11	2	0	0	13
12	66	58	26	6	2	0	0	8
13	72	58	21	9	5	0	0	14
14	83	64	41	14	1	2	2	19
15	97	84	15	7	6	0	0	13
16	74	61	25	9	2	1	1	13
17	67	52	36	14	0	1	0	15
18	97	69	30	21	5	2	0	28
19	91	65	27	17	3	2	4	26
20	81	71	42	9	0	1	0	10
21	65	43	23	11	5	5	1	22
22	83	58	28	20	0	3	2	25
23	56	47	18	6	3	0	0	9
24	90	79	37	7	0	0	4	11
25	67	59	36	6	0	0	2	8
26	76	50	22	21	3	1	1	26
**TOTAL**	2001	1553	739	338	50	31	29	448
**AVERAGE**	**77.0**	**59.7**	**28.4**	**13.0**	**1.9**	**1.2**	**1.1**	**17.2**
**SEM**	2.1	2.0	1.8	1.0	0.4	0.3	0.3	1.3
Stat. sig. (exp. vs. con. embryos; ST4a) *p<0.05, **p<0.005			**		**	**	*	
p-value (2-tailed students t-test)	2.41E-01	5.20E-01	9.46E-07	4.32E-01	2.46E-08	5.08E-04	1.08E-02	1.98E-01

Supplementary table ST4b

Supplementary tables ST4 (a & b): Individual embryo data used to generate averaged data presented in supplementary figure S2; *the average number of cells contributing to all blastocyst cell lineages in embryos in vitro cultured in the presence of vehicle control (DMSO) or p38-Mapk14/11 inhibitor (SB220025) from the early (E3.5) to late blastocyst (E4.5) stages in embryos immuno-fluorescently stained for Nanog and Cdx2.*

Supplementary tables ST5 (a-c)

DMSO (IF: Nanog/ Gata4)									
#	TOTAL NUMBER OF CELLS								
	EMBRYO	TE	Nanog -ve TE cells	Nanog +ve TE cells	ICM				
					Nanog	Gata4	Nanog/Gata4 (co-expressed)	Nanog/Gata 4 -ve	TOTAL
1	97	72	59	13	16	7	2	0	25
2	99	81	61	20	12	6	0	0	18
3	92	67	54	13	18	7	0	0	25
4	106	84	71	13	9	10	3	0	22
5	104	81	72	9	13	10	0	0	23
6	103	87	82	5	8	6	2	0	16
7	110	85	77	8	14	8	3	0	25
8	95	73	65	8	13	7	2	0	22
9	83	64	55	9	9	9	1	0	19
10	75	58	50	8	11	2	2	2	17
11	108	78	77	1	18	10	2	0	30
12	87	67	62	5	12	8	0	0	20
13	115	90	87	3	15	9	1	0	25
14	85	63	59	4	11	9	2	0	22
15	101	82	66	16	9	7	3	0	19
16	89	66	56	10	12	6	5	0	23
17	93	69	57	12	12	10	2	0	24
18	76	60	55	5	5	9	2	0	16
19	84	65	47	18	8	8	2	1	19
20	102	88	73	15	6	8	0	0	14
21	98	79	78	1	8	9	2	0	19
22	107	76	72	4	15	14	2	0	31
23	76	59	57	2	9	5	3	0	17
24	99	76	71	5	13	9	1	0	23
25	83	65	58	7	11	5	2	0	18
26	105	76	61	15	17	12	0	0	29
27	92	75	48	27	8	8	0	1	17
28	95	70	66	4	15	8	2	0	25
29	85	64	54	10	12	4	5	0	21
30	98	77	69	8	8	10	3	0	21
31	99	80	66	14	5	14	0	0	19
32	91	62	58	4	18	10	1	0	29
TOTAL	3032	2339	2043	296	370	264	55	4	693
**AVERAGE**	**94.8**	**73.1**	**63.8**	**9.3**	**11.6**	**8.3**	**1.7**	**0.1**	**21.7**
SEM	1.8	1.6	1.8	1.1	0.7	0.5	0.2	0.1	0.8

Supplementary table ST5a

p38-Mapk α/β inhibitor (SB203580) inactive analogue (SB202474, IF: Nanog/ Gata4)

#	TOTAL NUMBER OF CELLS								
	EMBRYO	TE	Nanog -ve TE cells	Nanog +ve TE cells	ICM				
					Nanog	Gata4	Nanog/Gata4 (co-expressed)	Nanog/Gata 4 -ve	TOTAL
1	80	69	56	13	5	4	2	0	11
2	94	73	69	4	7	13	1	0	21
3	85	71	51	20	5	8	1	0	14
4	102	80	70	10	12	7	2	1	22
5	83	66	63	3	7	9	1	0	17
6	96	77	73	4	12	7	0	0	19
7	101	76	54	22	12	12	1	0	25
8	85	65	61	4	8	10	2	0	20
9	94	72	66	6	12	10	0	0	22
10	86	67	58	9	8	11	0	0	19
11	110	89	80	9	6	14	1	0	21
12	106	75	66	9	17	12	2	0	31
13	106	82	72	10	10	13	0	1	24
14	92	71	64	7	14	6	1	0	21
15	104	79	76	3	17	8	0	0	25
16	82	58	54	4	16	7	1	0	24
17	89	67	62	5	11	11	0	0	22
18	90	69	68	1	16	4	1	0	21
19	100	79	65	14	11	9	1	0	21
20									
21									
22									
23									
24									
25									
26									
27									
28									
29									
30									
31									
32									
TOTAL	1785	1385	1228	157	206	175	17	2	400
**AVERAGE**	**93.9**	**72.9**	**64.6**	**8.3**	**10.8**	**9.2**	**0.9**	**0.1**	**21.1**
SEM	2.1	1.7	1.8	1.3	0.9	0.7	0.2	0.1	1.0
Stat. sig. (con. vs. con. embryos; ST5a) *p<0.05, **p<0.005							*		
p-value (2-tailed students t-test)	7.82E-01	9.36E-01	7.71E-01	5.66E-01	5.17E-01	2.26E-01	1.81E-02	8.60E-01	6.31E-01

Supplementary table ST5b

p38-Mapk α/β inhibitor (SB203580, IF: Nanog/ Gata4)

#	TOTAL NUMBER OF CELLS								
	EMBRYO	TE	Nanog -ve TE cells	Nanog +ve TE cells	ICM				
					Nanog	Gata4	Nanog/Gata4 (co-expressed)	Nanog/Gata 4 -ve	TOTAL
1	95	61	60	1	31	2	1	0	34
2	83	47	39	8	30	6	0	0	36
3	66	42	39	3	19	3	1	1	24
4	97	67	65	2	24	5	0	1	30
5	92	59	57	2	27	4	0	2	33
6	72	53	48	5	17	2	0	0	19
7	94	63	62	1	26	1	1	3	31
8	86	62	58	4	16	8	0	0	24
9	85	55	53	2	23	3	3	1	30
10	87	53	51	2	30	2	1	1	34
11	87	60	47	13	24	0	3	0	27
12	66	38	35	3	22	6	0	0	28
13	88	50	47	3	32	1	4	1	38
14	87	57	56	1	28	0	2	0	30
15	66	37	32	5	26	1	2	0	29
16	108	83	81	2	21	2	2	0	25
17	88	58	57	1	24	5	1	0	30
18	99	65	60	5	27	4	2	1	34
19									
20									
21									
22									
23									
24									
25									
26									
27									
28									
29									
30									
31									
32									
**TOTAL**	1546	1010	947	63	447	55	23	11	536
**AVERAGE**	**85.9**	**56.1**	**52.6**	**3.5**	**24.8**	**3.1**	**1.3**	**0.6**	**29.8**
**SEM**	2.8	2.6	2.8	0.7	1.1	0.5	0.3	0.2	1.1
Stat. sig. (exp. vs. con. embryos; ST5a) *p<0.05, **p<0.005	*	**	**	**	**	**		*	**
p-value (2-tailed students t-test)	8.15E-03	4.40E-07	9.02E-04	4.12E-04	8.50E-15	3.82E-09	2.58E-01	9.28E-03	1.65E-07
Stat. sig. (exp. vs. con. embryos; ST5b) §p<0.05, §§p<0.005	§	§§	§§	§§	§§	§§		§	§§
p-value (2-tailed students t-test)	2.63E-02	3.65E-06	8.80E-04	3.38E-03	1.17E-11	2.96E-08	2.55E-01	2.06E-02	1.16E-06

Supplementary table ST5c

Supplementary tables ST5 (a – c): Individual embryo data used to generate averaged data presented in supplementary figure S3; *the average number of cells contributing to all blastocyst cell lineages in embryos in vitro cultured in the presence of vehicle control (DMSO), the inactive analog of the p38-Mapk14/11 inhibiting drug SB203580 (SB202474) or the p38-Mapk14/11 inhibiting drug itself (S203580), from the early (E3.5) to late blastocyst (E4.5) stages in embryos immuno-fluorescently stained for Nanog and Gata4.*

Supplementary tables ST6 (a&b)

DMSO (IF: Nanog/ Gata6)									
#	TOTAL NUMBER OF CELLS								
	EMBRYO	TE	Nanog -ve TE cells	Nanog +ve TE cells	ICM				
					Nanog	Gata6	Nanog/Gata6 (co-expressed)	Nanog/Gata 6 -ve	TOTAL
1	104	89	65	24	5	9	1	0	15
2	114	92	71	21	7	13	2	0	22
3	80	64	50	14	9	7	0	0	16
4	103	86	81	5	4	8	5	0	17
5	94	72	59	13	6	12	4	0	22
6	96	72	63	9	6	14	4	0	24
7	86	66	53	13	4	10	6	0	20
8	94	76	60	16	4	10	4	0	18
9	89	72	62	10	7	10	0	0	17
10	73	55	46	9	4	7	7	0	18
11	80	64	42	22	5	9	2	0	16
12	99	81	59	22	7	7	4	0	18
13									
14									
**TOTAL**	1112	889	711	178	68	116	39	0	223
**AVERAGE**	**92.7**	**74.1**	**59.3**	**14.8**	**5.7**	**9.7**	**3.3**	**0.0**	**18.6**
**SEM**	3.4	3.2	3.1	1.8	0.5	0.7	0.7	0.0	0.8

Supplementary table ST6a

p38-Mapk α/β inhibitor (SB203580, IF: Nanog/ Gata6)									
	TOTAL NUMBER OF CELLS								
#	EMBRYO	TE	Nanog -ve TE cells	Nanog +ve TE cells	ICM				
					Nanog	Gata6	Nanog/Gata6 (co-expressed)	Nanog/Gata 6 -ve	TOTAL
1	95	62	41	21	3	7	23	0	33
2	63	42	40	2	0	7	14	0	21
3	95	65	50	15	0	2	28	0	30
4	99	70	57	13	3	8	18	0	29
5	89	65	51	14	4	4	16	0	24
6	47	30	22	8	1	4	12	0	17
7	99	75	66	9	1	5	18	0	24
8	96	61	60	1	5	15	15	0	35
9	91	71	64	7	1	1	18	0	20
10	94	51	43	8	3	5	35	0	43
11	105	67	65	2	2	4	32	0	38
12	90	65	64	1	4	8	13	0	25
13	101	74	72	2	2	7	18	0	27
14	97	75	62	13	4	7	11	0	22
**TOTAL**	1261	873	757	116	33	84	271	0	388
**AVERAGE**	**90.1**	**62.4**	**54.1**	**8.3**	**2.4**	**6.0**	**19.4**	**0.0**	**27.7**
**SEM**	4.2	3.5	3.7	1.7	0.4	0.9	2.0	0.0	2.0
Stat. sig. (exp. vs. con. embryos; ST6a) *p<0.05, **p<0.005		*		*	**	**	**		**
p-value (2-tailed students t-test)	6.43E-01	2.29E-02	2.99E-01	1.34E-02	2.27E-05	4.22E-03	2.00E-07	-	5.06E-04

Supplementary table ST6b

Supplementary tables ST6 (a & b): Individual embryo data used to generate averaged data presented in supplementary figure S5; *the average number of cells contributing to all blastocyst cell lineages in embryos in vitro cultured in the presence of vehicle control (DMSO) or the p38-Mapk14/11 inhibiting drug S203580, from the early (E3.5) to late blastocyst (E4.5) stages in embryos immuno-fluorescently stained for Nanog and Gata6.*

Supplementary tables ST7 (a-h)

+DMSO E3.5 - E4.5, IF: (Nanog/ Gata4)									
	TOTAL NUMBER OF CELLS								
#	EMBRYO	TE	Nanog -ve TE cells	Nanog +ve TE cells	ICM				
					Nanog	Gata4	Nanog/Gata4 (co-expressed)	Nanog/Gata4 -ve	TOTAL
1	69	44	31	13	15	3	6	1	25
2	96	69	59	10	14	8	5	0	27
3	86	64	57	7	14	8	0	0	22
4	90	66	48	18	11	6	7	0	24
5	78	56	39	17	10	12	0	0	22
6	81	62	37	25	13	5	1	0	19
7	83	57	54	3	11	8	5	2	26
8	104	85	66	19	8	10	1	0	19
9	64	52	50	2	5	4	3	0	12
10	87	67	55	12	11	7	2	0	20
11	85	71	69	2	4	9	1	0	14
12	101	75	69	6	11	11	4	0	26
13									
14									
15									
16									
17									
18									
19									
20									
21									
22									
23									
24									
25									
TOTAL	1024	768	634	134	127	91	35	3	256
**AVERAGE**	**85.3**	**64.0**	**52.8**	**11.2**	**10.6**	**7.6**	**2.9**	**0.3**	**21.3**
SEM	3.4	3.2	3.6	2.2	1.0	0.8	0.7	0.2	1.4

Supplementary table ST7a

+DMSO E3.5 - E4.0 & KSOM alone E4.0 - E4.5, (IF: Nanog/ Gata4)									
	TOTAL NUMBER OF CELLS								
					ICM				
#	EMBRYO	TE	Nanog -ve TE cells	Nanog +ve TE cells	Nanog	Gata4	Nanog/Gata4 (co-expressed)	Nanog/Gata4 -ve	TOTAL
1	62	43	29	14	15	4	0	0	19
2	119	87	85	2	6	26	0	0	32
3	99	78	63	15	8	10	3	0	21
4	63	34	27	7	22	5	2	0	29
5	87	66	45	21	13	6	2	0	21
6	100	80	73	7	7	12	1	0	20
7	90	60	49	11	17	9	4	0	30
8	99	75	47	28	13	11	0	0	24
9	96	70	50	20	12	7	7	0	26
10	94	67	44	23	19	6	0	2	27
11	86	68	61	7	6	11	1	0	18
12	72	48	35	13	13	7	4	0	24
13	86	61	40	21	18	6	0	1	25
14	58	34	29	5	18	4	0	2	24
15	92	70	56	14	10	11	1	0	22
16	87	63	58	5	9	15	0	0	24
17	102	79	71	8	10	13	0	0	23
18	86	66	55	11	5	14	1	0	20
19	49	31	30	1	8	9	1	0	18
20	91	69	55	14	14	6	2	0	22
21	102	74	51	23	11	15	2	0	28
22	76	60	50	10	7	7	2	0	16
23	81	64	63	1	7	10	0	0	17
24									
25									
TOTAL	1977	1447	1166	281	268	224	33	5	530
**AVERAGE**	**86.0**	**62.9**	**50.7**	**12.2**	**11.7**	**9.7**	**1.4**	**0.2**	**23.0**
SEM	3.4	3.2	3.1	1.6	1.0	1.0	0.4	0.1	0.9
Stat. sig. (con vs. con embryos; ST7a) *p<0.05, **p<0.005									
p-value (2-tailed students t-test)	9.08E-01	9.08E-01	9.08E-01	9.08E-01	9.08E-01	9.08E-01	9.08E-01	9.08E-01	9.08E-01

Supplementary table ST7b

+DMSO E3.75 - E4.5, (IF: Nanog/ Gata4)									
	TOTAL NUMBER OF CELLS								
					ICM				
#	EMBRYO	TE	Nanog -ve TE cells	Nanog +ve TE cells	Nanog	Gata4	Nanog/Gata4 (co- expressed)	Nanog/Gata4 -ve	TOTAL
1	83	71	45	26	7	5	0	0	12
2	70	53	41	12	8	8	1	0	17
3	92	70	55	15	11	9	2	0	22
4	87	65	54	11	7	10	5	0	22
5	88	64	64	0	15	6	3	0	24
6	88	70	55	15	11	7	0	0	18
7	87	59	55	4	18	9	1	0	28
8	87	68	58	10	9	6	4	0	19
9	69	56	55	1	6	7	0	0	13
10	87	60	45	15	14	11	2	0	27
11	98	74	54	20	15	7	2	0	24
12									
13									
14									
15									
16									
17									
18									
19									
20									
21									
22									
23									
24									
25									
**TOTAL**	936	710	581	129	121	85	20	0	226
**AVERAGE**	**85.1**	**64.5**	**52.8**	**11.7**	**11.0**	**7.7**	**1.8**	**0.0**	**20.5**
**SEM**	2.6	2.0	2.0	2.4	1.2	0.6	0.5	0.0	1.6
Stat. sig. (con vs. con embryos; ST7a) *p<0.05, **p<0.005									
p-value (2-tailed students t-test)	9.56E-01	8.88E-01	9.97E-01	8.63E-01	7.91E-01	8.85E-01	2.24E-01	1.97E-01	7.10E-01
Stat. sig. (con vs. con embryos; ST7b) §p<0.05, §§p<0.005									
p-value (2-tailed students t-test)	8.71E-01	7.39E-01	6.60E-01	8.63E-01	6.99E-01	1.99E-01	5.49E-01	2.42E-01	1.49E-01

Supplementary table ST7c

+DMSO E4.0 - E4.5, (IF: Nanog/ Gata4)

#	TOTAL NUMBER OF CELLS								
					ICM				
	EMBRYO	TE	Nanog -ve TE cells	Nanog +ve TE cells	Nanog	Gata4	Nanog/Gata4 (co-expressed)	Nanog/Gata4 -ve	TOTAL
1	106	81	73	8	9	13	3	0	25
2	79	63	57	6	11	5	0	0	16
3	82	59	46	13	10	9	4	0	23
4	99	69	63	6	15	11	4	0	30
5	48	38	31	7	8	2	0	0	10
6	105	83	77	6	10	10	2	0	22
7	96	70	54	16	16	9	1	0	26
8	85	58	51	7	17	5	4	1	27
9	94	74	71	3	6	11	3	0	20
10	88	68	57	11	7	11	1	1	20
11	75	53	47	6	11	7	4	0	22
12	88	58	53	5	13	9	8	0	30
13									
14									
15									
16									
17									
18									
19									
20									
21									
22									
23									
24									
25									
**TOTAL**	1045	774	680	94	133	102	34	2	271
**AVERAGE**	**87.1**	**64.5**	**56.7**	**7.8**	**11.1**	**8.5**	**2.8**	**0.2**	**22.6**
**SEM**	4.5	3.6	3.7	1.1	1.0	0.9	0.6	0.1	1.7
Stat. sig. (con vs. con embryos; ST7a) *p<0.05, **p<0.005									
p-value (2-tailed students t-test)	7.61E-01	7.61E-01	7.61E-01	7.61E-01	7.61E-01	7.61E-01	7.61E-01	7.61E-01	7.61E-01
Stat. sig. (con vs. con embryos; ST7b) §p<0.05, §§p<0.005									
p-value (2-tailed students t-test)	8.46E-01	8.46E-01	8.46E-01	8.46E-01	8.46E-01	8.46E-01	8.46E-01	8.46E-01	8.46E-01
Stat. sig. (con vs. con embryos; ST7c) p<0.05, ††p<0.005									
p-value (2-tailed students t-test)	7.14E-01	9.92E-01	3.86E-01	1.38E-01	9.58E-01	4.89E-01	2.36E-01	1.71E-01	3.86E-01

Supplementary table ST7d

+p38 Mapk14/11 inhibitor (SB220025) E3.5 - E4.5, (IF: Nanog/ Gata4)									
	TOTAL NUMBER OF CELLS								
					ICM				
#	EMBRYO	TE	Nanog -ve TE cells	Nanog +ve TE cells	Nanog	Gata4	Nanog/Gata4 (co-expressed)	Nanog/Gata4 -ve	TOTAL
1	33	30	24	6	3	0	0	0	3
2	70	45	18	27	22	1	2	0	25
3	58	35	16	19	23	0	0	0	23
4	81	51	35	16	26	3	0	1	30
5	87	56	36	20	28	1	2	0	31
6	78	64	19	45	10	2	0	2	14
7	89	77	61	16	6	6	0	0	12
8	68	47	26	21	13	5	2	1	21
9	85	60	32	28	24	0	0	1	25
10	90	62	40	22	27	0	0	1	28
11	64	45	28	17	12	1	0	6	19
12	71	61	43	18	10	0	0	0	10
13	61	45	30	15	13	1	2	0	16
14	66	52	35	17	11	0	1	2	14
15	78	59	49	10	14	4	0	1	19
16	96	82	57	25	10	4	0	0	14
17	78	46	29	17	30	0	0	2	32
18	68	51	47	4	7	7	3	0	17
19	67	51	50	1	10	5	0	1	16
20	54	42	41	1	10	2	0	0	12
21	80	64	56	8	12	4	0	0	16
22	81	45	41	4	31	2	2	1	36
23									
24									
25									
TOTAL	1603	1170	813	357	352	48	14	19	433
**AVERAGE**	**72.9**	**53.2**	**37.0**	**16.2**	**16.0**	**2.2**	**0.6**	**0.9**	**19.7**
SEM	3.0	2.6	2.7	2.2	1.8	0.5	0.2	0.3	1.8
Stat. sig. (exp. vs. con. embryos; ST7a) *p<0.05, **p<0.005	*	*	**		**	**	**		
p-value (2-tailed students t-test)	1.43E-02	1.64E-02	1.48E-03	1.44E-01	4.50E-02	5.43E-07	4.96E-04	1.49E-01	5.30E-01

Supplementary table ST7e

+Mapk14/11 (SB220025) E3.5 - E4.0 & KSOM alone E4.0 to E4.5, (IF: Nanog/ Gata4)

#	TOTAL NUMBER OF CELLS								
	EMBRYO	TE	Nanog -ve TE cells	Nanog +ve TE cells	ICM				TOTAL
					Nanog	Gata4	Nanog/Gata4 (co-expressed)	Nanog/Gata4 ve	
1	78	63	50	13	13	0	0	2	15
2	79	64	55	9	9	4	2	0	15
3	85	68	34	34	13	2	0	2	17
4	84	62	29	33	16	2	2	2	22
5	69	57	28	29	11	0	0	1	12
6	71	60	37	23	8	2	1	0	11
7	70	53	30	23	9	3	5	0	17
8	51	31	17	14	18	1	1	0	20
9	80	60	49	11	12	6	0	2	20
10	71	55	52	3	12	4	0	0	16
11	75	46	36	10	25	2	0	2	29
12	70	54	34	20	16	0	0	0	16
13	85	69	53	16	12	3	0	1	16
14	74	55	10	45	16	2	1	0	19
15	51	41	18	23	10	0	0	0	10
16	86	54	32	22	27	2	3	0	32
17	83	52	28	24	26	2	2	1	31
18	92	73	38	35	11	6	1	1	19
19	82	57	30	27	21	3	0	1	25
20	91	62	28	34	26	1	2	0	29
21	89	64	36	28	17	6	2	0	25
22	63	49	22	27	9	4	0	1	14
23	83	65	33	32	13	3	2	0	18
24	79	67	27	40	8	3	1	0	12
25	65	51	21	30	13	0	0	1	14
**TOTAL**	1906	1432	827	605	371	61	25	17	474
**AVERAGE**	**76.2**	**57.3**	**33.1**	**24.2**	**14.8**	**2.4**	**1.0**	**0.7**	**19.0**
**SEM**	2.2	1.9	2.3	2.1	1.2	0.4	0.3	0.2	1.3
Stat. sig. (exp. vs. con. embryos; ST7b) *p<0.05, **p<0.005	*		**	**	*	**		*	*
p-value (2-tailed students t-test)	1.91E-02	1.28E-01	3.96E-05	4.36E-05	4.74E-02	1.10E-08	3.26E-01	2.95E-02	1.25E-02
Stat. sig. (exp. vs. exp. embryos; ST7e) §p<0.05, §§p<0.005									
p-value (2-tailed students t-test)	3.62E-01	3.62E-01	3.62E-01	3.62E-01	3.62E-01	3.62E-01	3.62E-01	3.62E-01	3.62E-01

Supplementary table ST7f

+p38-Mapk14/11 inhibitor (SB220025) E3.75 - E4.5, (IF: Nanog/ Gata4)

#	TOTAL NUMBER OF CELLS				ICM				
	EMBRYO	TE	Nanog -ve TE cells	Nanog +ve TE cells	Nanog	Gata4	Nanog/Gata4 (co-expressed)	Nanog/Gata4 -ve	TOTAL
1	97	76	71	5	14	7	0	0	21
2	71	53	51	2	9	7	0	2	18
3	101	73	65	8	13	12	3	0	28
4	64	42	36	6	13	2	5	2	22
5	76	54	39	15	12	5	5	0	22
6	78	58	48	10	10	4	3	3	20
7	73	54	53	1	10	7	0	2	19
8	77	60	49	11	6	10	1	0	17
9	83	62	42	20	11	6	4	0	21
10	80	58	53	5	10	9	2	1	22
11	85	61	41	20	16	5	3	0	24
12	106	82	80	2	10	14	0	0	24
13									
14									
15									
16									
17									
18									
19									
20									
21									
22									
23									
24									
25									
**TOTAL**	991	733	628	105	134	88	26	10	258
**AVERAGE**	**82.6**	**61.1**	**52.3**	**8.8**	**11.2**	**7.3**	**2.2**	**0.8**	**21.5**
**SEM**	3.5	3.1	3.7	1.8	0.7	0.9	0.5	0.3	0.8
Stat. sig. (exp. vs.con. embryos; ST7c) *p<0.05, **p<0.005								*	
p-value (2-tailed students t-test)	5.89E-01	3.82E-01	9.15E-01	3.37E-01	9.06E-01	7.38E-01	6.51E-01	2.19E-02	5.93E-01
Stat. sig. (exp. vs. exp. embryos; ST7e) §p<0.05, §§p<0.005			§§	§		§§	§§		
p-value (2-tailed students t-test)	5.66E-02	7.49E-02	2.50E-03	3.03E-02	6.74E-02	7.56E-06	4.63E-03	9.48E-01	4.68E-01
Stat. sig. (exp. vs. exp. embryos; ST7f) †p<0.05, ††p<0.005			††	††	†	††	†		
p-value (2-tailed students t-test)	1.26E-01	2.83E-01	7.80E-05	4.10E-05	4.88E-02	2.06E-06	3.44E-02	6.35E-01	1.96E-01

Supplementary table ST7g

+p38-Mapk14/11 inhibitor (SB220025) E4.0 - E4.5, (IF: Nanog/ Gata4)									
#	TOTAL NUMBER OF CELLS								
					ICM				
	EMBRYO	TE	Nanog -ve TE cells	Nanog +ve TE cells	Nanog	Gata4	Nanog/Gata4 (co-expressed)	Nanog/Gata4 -ve	TOTAL
1	111	84	71	13	12	14	0	1	27
2	71	56	43	13	10	5	0	0	15
3	82	50	42	8	26	5	1	0	32
4	109	80	76	4	17	9	2	1	29
5	82	60	49	11	9	8	5	0	22
6	97	72	60	12	14	9	2	0	25
7	94	67	47	20	21	6	0	0	27
8	86	76	74	2	4	5	1	0	10
9	98	67	60	7	18	11	2	0	31
10	104	81	72	9	13	9	1	0	23
11	89	65	55	10	12	9	3	0	24
12	91	67	55	12	11	8	5	0	24
13	88	71	65	6	9	8	0	0	17
14	92	76	65	11	7	7	2	0	16
15									
16									
17									
18									
19									
20									
21									
22									
23									
24									
25									
TOTAL	1294	972	834	138	183	113	24	2	322
**AVERAGE**	**92.4**	**69.4**	**59.6**	**9.9**	**13.1**	**8.1**	**1.7**	**0.1**	**23.0**
SEM	2.9	2.6	3.1	1.2	1.6	0.7	0.5	0.1	1.7
Stat. sig. (exp. vs. con embryos; ST7d) *p<0.05, **p<0.005									
p-value (2-tailed students t-test)	3.19E-01	2.70E-01	5.50E-01	2.24E-01	3.12E-01	7.03E-01	1.60E-01	8.73E-01	8.64E-01
Stat. sig. (exp. vs. exp. embryos; ST7e) §p<0.05, §§p<0.005	§§	§§	§§	§		§§	§		
p-value (2-tailed students t-test)	1.05E-04	2.11E-04	5.94E-06	3.60E-02	2.70E-01	1.40E-08	2.13E-02	6.12E-02	2.10E-01
Stat. sig. (exp. vs. exp. embryos; ST7f) †p<0.05, ††p<0.005	††	††	††	††		††		†	
p-value (2-tailed students t-test)	7.93E-05	4.81E-04	4.72E-08	1.97E-05	3.74E-01	1.24E-09	1.41E-01	2.35E-02	6.47E-02
Stat. sig. (exp. vs. exp. Embryos; ST7g) #p<0.05, ##p<0.005	#							#	
p-value (2-tailed students t-test)	4.42E-02	5.23E-02	1.52E-01	6.18E-01	3.06E-01	5.32E-01	5.31E-01	3.83E-02	4.66E-01

Supplementary table ST7h

Supplementary tables ST7 (a - h): Individual embryo data used to generate averaged data presented in figure 2 and supplementary figure S8 (p38-Mapk14/11 inhibition); *the average number of cells contributing to all blastocyst cell lineages in embryos in vitro cultured to the late blastocyst (E4.5) stage in the presence of vehicle control (DMSO) or p38-Mapk14/11 inhibitor (SB220025) from the following blastocyst stage; E3.5, E3.75 and E4.0. Note an additional condition, in which embryos were cultured in control or inhibitor from the early blastocyst (E3.5) stage until the mid blastocyst (E4.0) stage and then transferred back to conventional growth media before continued culture to the late blastocyst (E4.5) stage, was also included. Embryos were immuno-fluorescently stained for Nanog and Gata4.*

Supplementary tables ST8 (a-h)

+DMSO E3.5 - E4.5, IF: (Nanog/ Gata4)									
	TOTAL NUMBER OF CELLS								
#	EMBRYO	TE	Nanog -ve TE cells	Nanog +ve TE cells	ICM				
					Nanog	Gata4	Nanog/Gata4 co-expressed)	Nanog/Gata4 -ve	TOTAL
1	89	58	47	11	19	12	0	0	31
2	78	53	39	14	13	8	4	0	25
3	92	71	52	19	11	8	2	0	21
4	85	64	47	17	5	13	2	1	21
5	79	52	47	5	12	10	4	1	27
6	91	65	50	10	17	6	3	0	26
7	92	61	60	1	17	5	8	1	31
8	70	44	43	1	16	8	2	0	26
9	86	61	51	10	12	4	9	0	25
10	82	58	41	17	14	7	3	0	24
11	107	83	72	11	12	10	2	0	24
12	93	75	59	16	12	2	2	2	18
13	90	70	46	24	14	4	2	0	20
14									
15									
16									
17									
18									
19									
20									
21									
22									
23									
24									
25									
TOTAL	1134	815	654	156	174	97	43	5	319
**AVERAGE**	**87.2**	**62.7**	**50.3**	**12.0**	**13.4**	**7.5**	**3.3**	**0.4**	**24.5**
SEM	2.5	2.9	2.5	1.9	1.0	0.9	0.7	0.2	1.1

Supplementary table ST8a

+DMSO E3.5 - E4.0 & KSOM alone E4.0 - E4.5, (IF: Nanog/ Gata4)									
#	TOTAL NUMBER OF CELLS								
	EMBRYO	TE	Nanog -ve TE cells	Nanog +ve TE cells	ICM				
					Nanog	Gata4	Nanog/Gata4 co-expressed)	Nanog/Gata4 -ve	TOTAL
1	93	67	62	5	15	7	4	0	26
2	119	88	75	13	11	19	1	0	31
3	73	56	40	16	13	2	1	1	17
4	83	58	43	15	18	2	5	0	25
5	81	60	46	14	8	11	1	1	21
6	68	46	44	2	9	12	1	0	22
7	98	65	65	0	11	22	0	0	33
8	108	79	70	9	15	12	2	0	29
9	98	79	60	19	7	9	3	0	19
10	58	39	34	5	12	6	1	0	19
11	69	45	25	20	21	1	2	0	24
12	75	53	28	25	15	4	0	3	22
13	82	64	59	5	6	10	2	0	18
14	78	54	41	13	14	6	4	0	24
15	87	62	49	13	19	6	0	0	25
16	78	54	45	9	14	5	5	0	24
17	89	69	65	4	10	8	2	0	20
18	67	50	42	8	10	6	1	0	17
19	70	50	44	6	12	8	0	0	20
20	87	59	44	15	17	9	2	0	28
21									
22									
23									
24									
25									
**TOTAL**	1661	1197	981	216	257	165	37	5	464
**AVERAGE**	**83.1**	**59.9**	**49.1**	**10.8**	**12.9**	**8.3**	**1.9**	**0.3**	**23.2**
**SEM**	3.3	2.8	3.1	1.5	0.9	1.2	0.4	0.2	1.0
Stat. sig. (con vs. con embryos; ST8a) *p<0.05, **p<0.005							*		
p-value (2-tailed students t-test)	3.24E-01	4.52E-01	6.59E-01	7.13E-01	6.47E-01	6.14E-01	3.74E-02	6.34E-01	3.52E-01

Supplementary table ST8b

+DMSO E3.75 - E4.5, (IF: Nanog/ Gata4)									
	TOTAL NUMBER OF CELLS								
					ICM				
#	EMBRYO	TE	Nanog -ve TE cells	Nanog +ve TE cells	Nanog	Gata4	Nanog/Gata4 co-expressed)	Nanog/Gata4 -ve	TOTAL
1	64	50	47	3	6	7	1	0	14
2	89	68	59	9	12	7	2	0	21
3	81	63	59	4	6	10	2	0	18
4	80	60	46	14	8	9	3	0	20
5	77	54	47	7	11	6	5	1	23
6	95	69	54	15	15	9	2	0	26
7	78	58	48	10	9	9	2	0	20
8	78	51	39	12	14	11	2	0	27
9	80	68	61	7	7	5	0	0	12
10	77	60	41	19	7	8	2	0	17
11	94	76	68	8	5	13	0	0	18
12	78	59	39	20	10	6	3	0	19
13	93	62	56	6	19	8	4	0	31
14	91	72	35	37	9	6	3	1	19
15									
16									
17									
18									
19									
20									
21									
22									
23									
24									
25									
**TOTAL**	1155	870	699	171	138	114	31	2	285
**AVERAGE**	**82.5**	**62.1**	**49.9**	**12.2**	**9.9**	**8.1**	**2.2**	**0.1**	**20.4**
**SEM**	2.3	2.1	2.6	2.4	1.1	0.6	0.4	0.1	1.4
Stat. sig. (con vs. con embryos; ST8a) *p<0.05, **p<0.005					*				
p-value (2-tailed students t-test)	1.79E-01	8.77E-01	9.17E-01	9.45E-01	2.29E-02	5.28E-01	1.70E-01	2.40E-01	2.48E-02
Stat. sig. (con vs. con embryos; ST8b) §p<0.05, §§p<0.005					*				
p-value (2-tailed students t-test)	9.02E-01	5.43E-01	8.39E-01	5.95E-01	4.02E-02	9.43E-01	4.94E-01	6.11E-01	9.74E-02

Supplementary table ST8c

+DMSO E4.0 - E4.5, (IF: Nanog/ Gata4)

#	TOTAL NUMBER OF CELLS								
					ICM				
	EMBRYO	TE	Nanog -ve TE cells	Nanog +ve TE cells	Nanog	Gata4	Nanog/Gata4 co-expressed)	Nanog/Gata4 -ve	TOTAL
1	73	51	39	12	13	7	2	0	22
2	102	73	56	17	13	11	5	0	29
3	88	74	52	22	9	5	0	0	14
4	84	59	55	4	10	13	2	0	25
5	87	70	52	18	6	11	0	0	17
6	90	65	53	12	8	13	4	0	25
7	92	70	63	7	12	5	5	0	22
8	95	75	48	27	8	7	4	1	20
9	83	61	60	1	9	11	2	0	22
10	85	61	57	4	10	13	0	1	24
11	83	65	43	22	12	4	2	0	18
12	102	71	67	4	15	15	1	0	31
13	92	63	46	17	16	8	5	0	29
14									
15									
16									
17									
18									
19									
20									
21									
22									
23									
24									
25									
**TOTAL**	1156	858	691	167	141	123	32	2	298
**AVERAGE**	**88.9**	**66.0**	**53.2**	**12.8**	**10.8**	**9.5**	**2.5**	**0.2**	**22.9**
**SEM**	2.2	1.9	2.2	2.3	0.8	1.0	0.5	0.1	1.4
Stat. sig. (con vs. con embryos; ST8a) *p<0.05, **p<0.005									
p-value (2-tailed students t-test)	6.18E-01	3.51E-01	3.98E-01	7.80E-01	5.76E-02	1.53E-01	3.48E-01	2.79E-01	3.67E-01
Stat. sig. (con vs. con embryos; ST8b) §p<0.05, §§p<0.005									
p-value (2-tailed students t-test)	2.04E-01	1.13E-01	3.38E-01	4.38E-01	1.33E-01	4.75E-01	3.31E-01	6.60E-01	8.71E-01
Stat. sig. (con vs. con embryos; ST8c) †p<0.05, ††p<0.005									
p-value (2-tailed students t-test)	5.78E-02	1.88E-01	3.57E-01	8.50E-01	4.74E-01	2.62E-01	7.04E-01	9.39E-01	1.97E-01

Supplementary table ST8d

+Mek1/2 inhibitor (SB220025) E3.5 - E4.5, (IF: Nanog/ Gata4)									
	TOTAL NUMBER OF CELLS								
					ICM				
#	EMBRYO	TE	Nanog -ve TE cells	Nanog +ve TE cells	Nanog	Gata4	Nanog/Gata4 co-expressed)	Nanog/Gata4 -ve	TOTAL
1	90	55	51	4	35	0	0	0	35
2	97	70	63	7	27	0	0	0	27
3	88	54	49	5	32	0	1	1	34
4	105	73	67	6	32	0	0	0	32
5	71	65	51	14	6	0	0	0	6
6	103	66	51	15	35	0	2	0	37
7	75	52	44	8	23	0	0	0	23
8	93	67	67	0	25	0	0	1	26
9	82	59	59	0	23	0	0	0	23
10	102	59	54	5	43	0	0	0	43
11	95	57	55	2	38	0	0	0	38
12	78	48	43	5	24	4	2	0	30
13	105	72	69	3	33	0	0	0	33
14	134	79	73	6	55	0	0	0	55
15	92	56	53	3	36	0	0	0	36
16	98	80	65	15	18	0	0	0	18
17	104	60	51	9	42	0	0	2	44
18	107	83	64	19	24	0	0	0	24
19	110	69	62	7	41	0	0	0	41
20	99	71	60	11	27	0	1	0	28
21	82	54	44	10	25	3	0	0	28
22	82	53	40	13	29	0	0	0	29
23	73	53	44	9	20	0	0	0	20
24									
25									
**TOTAL**	2165	1455	1279	176	693	7	6	4	710
**AVERAGE**	**94.1**	**63.3**	**55.6**	**7.7**	**30.1**	**0.3**	**0.3**	**0.2**	**30.9**
**SEM**	3.0	2.1	2.0	1.1	2.1	0.2	0.1	0.1	2.1
Stat. sig. (exp vs. con embryos; ST8a) *p<0.05, **p<0.005				*	**	**	**		*
p-value (2-tailed students t-test)	1.31E-01	8.73E-01	1.09E-01	3.63E-02	2.09E-06	1.87E-11	3.34E-06	2.80E-01	3.95E-02

Supplementary table ST8e

+Mek1/2 (SB220025) E3.5 - E4.0 & KSOM alone E4.0 to E4.5, (IF: Nanog/ Gata4)									
	TOTAL NUMBER OF CELLS								
					ICM				
#	EMBRYO	TE	Nanog -ve TE cells	Nanog +ve TE cells	Nanog	Gata4	Nanog/Gata4 co-expressed)	Nanog/Gata4 ve	TOTAL
1	78	64	53	11	5	5	4	0	14
2	70	45	31	14	17	5	2	1	25
3	72	55	53	2	7	9	1	0	17
4	91	59	57	2	31	0	0	1	32
5	86	60	54	6	12	14	0	0	26
6	75	56	39	17	10	5	2	2	19
7	91	58	58	0	20	11	0	2	33
8	73	52	51	1	13	7	0	1	21
9	83	58	55	3	10	12	3	0	25
10	97	70	70	0	25	0	0	2	27
11	82	64	54	10	6	12	0	0	18
12	95	66	60	6	11	14	4	0	29
13	82	64	47	17	9	5	2	2	18
14	61	44	36	8	11	4	2	0	17
15	98	70	60	10	20	8	0	0	28
16	68	50	45	5	12	5	0	1	18
17	92	69	66	3	20	1	2	0	23
18	121	93	91	2	21	7	0	0	28
19	87	63	59	4	13	8	3	0	24
20	76	60	52	8	7	9	0	0	16
21	93	74	52	22	8	10	1	0	19
22	56	34	31	3	12	6	4	0	22
23	60	42	25	17	14	3	1	0	18
24									
25									
**TOTAL**	1887	1370	1199	171	314	160	31	12	517
**AVERAGE**	**82.0**	**59.6**	**52.1**	**7.4**	**13.7**	**7.0**	**1.3**	**0.5**	**22.5**
**SEM**	3.1	2.6	3.0	1.3	1.4	0.8	0.3	0.2	1.1
Stat. sig. (exp vs. con embryos; ST8b) *p<0.05, **p<0.005									
p-value (2-tailed students t-test)	8.26E-01	9.40E-01	4.75E-01	9.47E-02	6.37E-01	3.69E-01	2.89E-01	2.47E-01	6.40E-01
Stat. sig. (exp. vs. exp embryos; ST8e) §p<0.05, §§p<0.005	**§§**				**§§**	**§§**	**§§**		**§§**
p-value (2-tailed students t-test)	7.65E-03	2.71E-01	3.33E-01	8.98E-01	5.67E-08	1.44E-09	2.05E-03	7.99E-02	1.10E-03

Supplementary table ST8f

+Mek1/2 (SB220025) E3.75 - E4.5, (IF: Nanog/ Gata4)									
	TOTAL NUMBER OF CELLS								
					ICM				
#	EMBRYO	TE	Nanog -ve TE cells	Nanog +ve TE cells	Nanog	Gata4	Nanog/Gata4 co-expressed)	Nanog/Gata4 ve	TOTAL
1	99	70	59	11	27	2	0	0	29
2	81	55	35	20	18	2	6	0	26
3	108	64	57	7	42	2	0	0	44
4	109	58	58	0	51	0	0	0	51
5	106	61	60	1	38	7	0	0	45
6	75	48	42	6	25	2	0	0	27
7	102	71	68	3	21	9	0	1	31
8	87	55	52	3	30	2	0	0	32
9	82	47	47	0	34	0	0	1	35
10	103	61	58	3	42	0	0	0	42
11	90	63	55	8	25	2	0	0	27
12	108	57	55	2	46	3	1	1	51
13	97	57	57	0	40	0	0	0	40
14	68	47	38	9	21	0	0	0	21
15									
16									
17									
18									
19									
20									
21									
22									
23									
24									
25									
**TOTAL**	1315	814	741	73	460	31	7	3	501
**AVERAGE**	**93.9**	**58.1**	**52.9**	**5.2**	**32.9**	**2.2**	**0.5**	**0.2**	**35.8**
**SEM**	3.6	2.0	2.5	1.5	2.8	0.7	0.4	0.1	2.6
Stat. sig. (exp. vs. con embryos; ST8c) *p<0.05, **p<0.005	**			*	**	**	**		**
p-value (2-tailed students t-test)	2.25E-03	2.86E-01	2.52E-01	1.80E-02	1.91E-08	2.56E-06	8.30E-03	5.74E-01	3.58E-06
Stat. sig. (exp. vs. exp embryos; ST8e) §p<0.05, §§p<0.005						§§			
p-value (2-tailed students t-test)	9.67E-01	1.09E-01	4.05E-01	1.79E-01	4.39E-01	4.03E-03	5.23E-01	8.01E-01	1.56E-01
Stat. sig. (exp. vs. exp embryos; ST8f) †p<0.05, ††p<0.005	†				††	††			††
p-value (2-tailed students t-test)	1.95E-02	7.01E-01	8.52E-01	2.86E-01	5.08E-08	4.54E-04	1.09E-01	1.90E-01	4.95E-06

Supplementary table ST8g

+Mek1/2 (SB220025) E4.0 - E4.5, (IF: Nanog/ Gata4)									
	TOTAL NUMBER OF CELLS								
					ICM				
#	EMBRYO	TE	Nanog -ve TE cells	Nanog +ve TE cells	Nanog	Gata4	Nanog/Gata4 co-expressed)	Nanog/Gata4 -ve	TOTAL
1	87	59	48	11	22	3	3	0	28
2	101	59	51	8	38	4	0	0	42
3	89	54	51	3	33	0	2	0	35
4	84	53	47	6	26	2	3	0	31
5	83	45	31	14	38	0	0	0	38
6	94	70	62	8	17	5	2	0	24
7	111	74	69	5	28	8	0	1	37
8	88	53	51	2	32	2	1	0	35
9	101	81	72	9	18	1	1	0	20
10	84	55	50	5	21	8	0	0	29
11	54	37	33	4	17	0	0	0	17
12	95	55	44	11	39	0	0	1	40
13	101	75	70	5	22	4	0	0	26
14									
15									
16									
17									
18									
19									
20									
21									
22									
23									
24									
25									
TOTAL	1172	770	679	91	351	37	12	2	402
**AVERAGE**	**90.2**	**59.2**	**52.2**	**7.0**	**27.0**	**2.8**	**0.9**	**0.2**	**30.9**
SEM	3.8	3.5	3.6	1.0	2.3	0.8	0.3	0.1	2.2
Stat. sig. (exp. vs. con embryos; ST8d) *p<0.05, **p<0.005				*	**	**	*		**
p-value (2-tailed students t-test)	7.83E-01	1.03E-01	8.29E-01	2.91E-02	7.00E-07	2.81E-05	2.26E-02	1.00E+00	4.59E-03
Stat. sig. (exp. vs. exp embryos; ST8e) §p<0.05, §§p<0.005						§§	§		
p-value (2-tailed students t-test)	4.27E-01	2.97E-01	3.76E-01	6.83E-01	3.51E-01	4.43E-04	3.40E-02	8.99E-01	9.87E-01
Stat. sig. (exp. vs. exp embryos; ST8f) †p<0.05, ††p<0.005					††	††			††
p-value (2-tailed students t-test)	1.15E-01	9.39E-01	9.83E-01	8.21E-01	6.02E-06	2.83E-03	3.79E-01	1.25E-01	4.93E-04
Stat. sig. (exp. vs. exp embryo;s ST8g) #p<0.05, ##p<0.005									
p-value (2-tailed students t-test)	4.78E-01	7.86E-01	8.73E-01	3.33E-01	1.20E-01	5.59E-01	4.47E-01	7.00E-01	1.65E-01

Supplementary table ST8h

Supplementary tables ST8 (a - h): Individual embryo data used to generate averaged data presented in figure 2 and supplementary figure S9 (Mek1/2 inhibition); *the average number of cells contributing to all blastocyst cell lineages in embryos in vitro cultured to the late blastocyst (E4.5) stage in the presence of vehicle control (DMSO) orMek1/2 inhibitor (PD0325901) from the following blastocyst stage; E3.5, E3.75 and E4.0. Note an additional condition, in which embryos were cultured in control or inhibitor from the early blastocyst (E3.5) stage until the mid blastocyst (E4.0) stage and then transferred back to conventional growth media before continued culture to the late blastocyst (E4.5) stage, was also included. Embryos were immuno-fluorescently stained for Nanog and Gata4.*

Supplementary tables ST9 (a&b)

+DMSO E3.5 - E4.0, IF: (Nanog/ Sox17)									
	TOTAL NUMBER OF CELLS								
					ICM				
#	EMBRYO	TE	Nanog -ve TE cells	Nanog +ve TE cells	Nanog	Sox17	Nanog/Sox17 (co-expressed)	Nanog/Sox17 ve	TOTAL
1	60	36	18	18	11	3	10	0	24
2	59	32	29	3	14	4	8	1	27
3	62	37	30	7	6	7	12	0	25
4	45	34	24	10	2	3	6	0	11
5	53	30	14	16	7	2	14	0	23
6	64	42	30	12	3	3	16	0	22
7	56	36	22	14	5	4	11	0	20
8	66	43	36	7	4	3	16	0	23
9	65	34	18	16	11	5	15	0	31
10	58	40	30	10	5	3	10	0	18
11	66	49	43	6	10	5	2	0	17
12	57	36	30	6	7	5	8	1	21
13	53	31	25	6	5	10	7	0	22
TOTAL	764	480	349	131	90	57	135	2	284
AVERAGE	**58.8**	**36.9**	**26.8**	**10.1**	**6.9**	**4.4**	**10.4**	**0.2**	**21.8**
SEM	1.7	1.5	2.2	1.3	1.0	0.6	1.2	0.1	1.4

Supplementary table ST9a

+p38 Mapk14/11 inhibitor (SB220025) E3.5 - E4.0, (IF: Nanog/ Sox17)									
	TOTAL NUMBER OF CELLS								
#	EMBRYO	TE	Nanog -ve TE cells	Nanog +ve TE cells	ICM				
					Nanog	Sox17	Nanog/Sox17 (co-expressed)	Nanog/Sox17 -ve	TOTAL
1	61	38	20	18	4	3	16	0	23
2	54	31	12	19	0	0	21	2	23
3	51	33	19	14	5	4	8	1	18
4	48	24	16	8	0	4	20	0	24
5	57	31	27	4	7	2	17	0	26
6	62	43	33	10	3	4	10	2	19
7	65	39	29	10	2	3	21	0	26
8	53	30	16	14	0	1	22	0	23
9	51	27	18	9	0	5	19	0	24
10	44	30	27	3	0	2	12	0	14
11	55	35	29	6	0	5	15	0	20
12	52	32	19	13	0	3	17	0	20
13	45	26	18	8	0	2	17	0	19
TOTAL	698	419	283	136	21	38	215	5	279
**AVERAGE**	**53.7**	**32.2**	**21.8**	**10.5**	**1.6**	**2.9**	**16.5**	**0.4**	**21.5**
SEM	1.8	1.5	1.8	1.4	0.7	0.4	1.2	0.2	1.0
Stat. sig. (exp vs. con embryos; ST9a) *p<0.05, **p<0.005	*	*			**	*	**		
p-value (2-tailed students t-test)	4.88E-02	3.59E-02	8.43E-02	8.41E-01	1.69E-04	5.51E-02	1.23E-03	3.40E-01	8.19E-01

Supplementary table ST9b

Supplementary tables ST9 (a & b): Individual embryo data used to generate averaged data presented in figure 3 (p38-Mapk14/11 inhibition); *the average number of cells contributing to all blastocyst cell lineages in embryos in vitro cultured from the early blastocyst (E3.5) stage until the mid-blastocyst (E4.0) stage in the presence of vehicle control (DMSO) or p38-Mapk14/11 inhibitor (SB220025) and immuno-fluorescently stained for Nanog and Sox17.*

Supplementary tables ST10 (a&b)

+DMSO E3.5 - E4.0, IF: (Nanog/ Sox17)									
	TOTAL NUMBER OF CELLS								
					ICM				
#	EMBRYO	TE	Nanog -ve TE cells	Nanog +ve TE cells	Nanog	Sox17	Nanog/Sox17 (co-expressed)	Nanog/Sox17 ve	TOTAL
1	60	37	21	16	10	5	8	0	23
2	61	37	23	14	11	3	10	0	24
3	54	34	19	15	11	1	8	0	20
4	60	35	12	23	7	7	11	0	25
5	62	36	20	16	17	3	6	0	26
6	62	40	24	16	14	4	4	0	22
7	57	33	16	17	9	7	8	0	24
8	47	26	10	16	12	2	7	0	21
9	53	34	18	16	11	3	5	0	19
10	55	31	26	5	15	2	7	0	24
11	69	47	34	13	9	7	6	0	22
12	58	32	15	17	7	12	7	0	26
13									
TOTAL	698	422	238	184	133	56	87	0	276
**AVERAGE**	**58.2**	**35.2**	**19.8**	**15.3**	**11.1**	**4.7**	**7.3**	**0.0**	**23.0**
SEM	1.6	1.5	1.9	1.2	0.9	0.9	0.6	0.0	0.7

Supplementary table ST10a

+Mek1/2 inhibitor (SB220025) E3.5 - E4.0, (IF: Nanog/ Sox17)									
	TOTAL NUMBER OF CELLS								
					ICM				
#	EMBRYO	TE	Nanog -ve TE cells	Nanog +ve TE cells	Nanog	Sox17	Nanog/Sox17 (co-expressed)	Nanog/Sox17 -ve	TOTAL
1	56	33	21	12	11	4	8	0	23
2	54	28	18	10	23	0	3	0	26
3	62	35	31	4	8	3	16	0	27
4	67	32	20	12	20	3	12	0	35
5	57	37	28	9	10	0	10	0	20
6	60	37	33	4	12	3	8	0	23
7	51	28	18	10	19	0	4	0	23
8	64	39	35	4	12	5	6	2	25
9	48	32	25	7	13	0	3	0	16
10	55	26	13	13	13	1	14	1	29
11	59	35	21	14	20	0	3	1	24
12	60	33	20	13	16	4	6	1	27
13	60	34	18	16	18	2	5	1	26
TOTAL	753	429	301	128	195	25	98	6	324
**AVERAGE**	**57.9**	**33.0**	**23.2**	**9.8**	**15.0**	**1.9**	**7.5**	**0.5**	**24.9**
SEM	1.4	1.1	1.9	1.1	1.3	0.5	1.2	0.2	1.3
Stat. sig. (exp vs. con embryo;s ST10a) *p<0.05, **p<0.005				**	*	*		*	
p-value (2-tailed students t-test)	8.54E-01	2.44E-01	1.82E-01	1.07E-03	3.45E-02	1.63E-02	7.25E-01	4.20E-02	2.37E-01

Supplementary table ST10b

Supplementary tables ST10 (a & b): Individual embryo data used to generate averaged data presented in figure 3 (Mek1/2 inhibition); *the average number of cells contributing to all blastocyst cell lineages in embryos in vitro cultured from the early blastocyst (E3.5) stage until the mid-blastocyst (E4.0) stage in the presence of vehicle control (DMSO) orMek1/2 inhibitor (PD0325901) and immuno-fluorescently stained for Nanog and Sox17.*

Supplementary tables ST11 (a-e)

+DMSO, GFP mRNA (+OGDB) microinjected									
	TOTAL NUMBER OF CELLS								
					ICM				
#	EMBRYO	TE	Nanog -ve TE cells	Nanog +ve TE cells	Nanog	Gata4	Nanog/Gata4 (co-expressed)	Nanog/Gata4 -ve	TOTAL
1	69	56	46	10	8	3	2	0	13
2	75	58	47	11	9	8	0	0	17
3	79	58	51	7	11	8	2	0	21
4	91	67	64	3	16	7	1	0	24
5	96	75	55	20	15	4	1	1	21
6	91	66	60	6	11	8	6	0	25
7	78	58	52	6	7	11	1	1	20
8	76	54	46	8	12	8	2	0	22
9	119	103	89	14	4	11	1	0	16
10	74	47	41	6	16	8	2	1	27
11	57	39	29	10	13	3	2	0	18
12	79	62	45	17	9	6	2	0	17
13	88	69	64	5	9	8	1	1	19
14	85	53	44	9	24	7	1	0	32
15	91	70	58	12	11	10	0	0	21
16	67	46	38	8	15	5	0	1	21
17	81	58	52	6	13	7	3	0	23
18	93	73	59	14	12	7	0	1	20
19	85	56	50	6	16	10	1	2	29
20	76	58	48	10	11	7	0	0	18
21	70	49	40	9	15	5	0	1	21
22	97	67	64	3	16	13	0	1	30
23	91	70	64	6	12	9	0	0	21
24									
TOTAL	1908	1412	1206	206	285	173	28	10	496
**AVERAGE**	**83.0**	**61.4**	**52.4**	**9.0**	**12.4**	**7.5**	**1.2**	**0.4**	**21.6**
SEM	2.7	2.7	2.6	0.9	0.9	0.5	0.3	0.1	1.0

Supplementary table ST11a

+Fgf-receptor inhibitor (SU5402), GFP mRNA (+OGDB) microinjected									
	TOTAL NUMBER OF CELLS								
#					ICM				
	EMBRYO	TE	Nanog -ve TE cells	Nanog +ve TE cells	Nanog	Gata4	Nanog/Gata4 (co-expressed)	Nanog/Gata4 -ve	TOTAL
1	74	68	53	15	5	0	0	1	6
2	81	49	33	16	25	7	0	0	32
3	75	57	52	5	15	0	1	2	18
4	74	55	45	10	19	0	0	0	19
5	49	41	28	13	5	0	2	1	8
6	55	34	25	9	15	4	2	0	21
7	72	54	42	12	17	0	0	1	18
8	73	51	47	4	16	5	1	0	22
9	85	56	46	10	20	5	3	1	29
10	94	61	55	6	25	6	2	0	33
11	44	26	17	9	16	0	0	2	18
12	68	51	37	14	17	0	0	0	17
13	92	71	61	10	15	4	1	1	21
14	65	47	37	10	18	0	0	0	18
15	66	48	35	13	16	1	1	0	18
16	67	41	18	23	25	0	0	1	26
17	91	70	61	9	13	5	1	2	21
18	96	67	63	4	25	1	1	2	29
19	57	36	22	14	20	0	0	1	21
20	62	41	26	15	18	0	2	1	21
21	64	57	41	16	7	0	0	0	7
22	66	53	36	17	10	2	0	1	13
23	82	63	41	22	13	3	2	1	19
24									
**TOTAL**	1652	1197	921	276	375	43	19	18	455
**AVERAGE**	**71.8**	**52.0**	**40.0**	**12.0**	**16.3**	**1.9**	**0.8**	**0.8**	**19.8**
**SEM**	2.9	2.5	2.8	1.1	1.2	0.5	0.2	0.2	1.5
Stat. sig. (exp. vs. con. embryos; ST11a) *p<0.05, **p<0.005	**	*	**	*	*	**			
p-value (2-tailed students t-test)	7.55E-03	1.43E-02	2.27E-03	3.25E-02	1.18E-02	8.08E-10	2.67E-01	8.38E-02	3.18E-01

Supplementary table ST11b

+DMSO, Mkk6-EE mRNA (+OGDBs) microinjected									
#	TOTAL NUMBER OF CELLS								
					ICM				
	EMBRYO	TE	Nanog -ve TE cells	Nanog +ve TE cells	Nanog	Gata4	Nanog/Gata4 (co-expressed)	Nanog/Gata4 -ve	TOTAL
1	70	60	58	2	4	6	0	0	10
2	67	58	58	0	2	7	0	0	9
3	89	74	74	0	5	7	2	1	15
4	66	49	41	8	8	8	1	0	17
5	57	49	48	1	4	3	1	0	8
6	45	34	30	4	1	9	0	1	11
7	73	63	54	9	1	8	1	0	10
8	64	52	50	2	0	12	0	0	12
9	73	57	52	5	4	12	0	0	16
10	88	74	74	0	0	13	0	1	14
11	72	67	65	2	0	5	0	0	5
12	84	74	72	2	1	9	0	0	10
13	45	38	38	0	2	5	0	0	7
14	73	59	58	1	3	11	0	0	14
15	62	55	53	2	1	6	0	0	7
16	85	75	72	3	0	10	0	0	10
17	78	65	64	1	2	10	0	1	13
18	59	47	47	0	2	10	0	0	12
19	55	48	43	5	0	7	0	0	7
20	66	51	41	10	9	6	0	0	15
21	66	57	56	1	0	9	0	0	9
22	70	60	60	0	0	10	0	0	10
23	50	36	28	8	9	4	0	1	14
24	70	61	57	4	0	9	0	0	9
**TOTAL**	1627	1363	1293	70	58	196	5	5	264
**AVERAGE**	**67.8**	**56.8**	**53.9**	**2.9**	**2.4**	**8.2**	**0.2**	**0.2**	**11.0**
**SEM**	2.5	2.4	2.6	0.6	0.6	0.5	0.1	0.1	0.7
Stat. sig. (exp. vs. con. embryos; ST11a) *p<0.05, **p<0.005	**			**	**		**		**
p-value (2-tailed students t-test)	1.46E-04	2.07E-01	6.97E-01	1.30E-06	1.28E-12	3.96E-01	1.63E-03	1.34E-01	7.96E-12

Supplementary table ST11c

+Fgf-receptor inhibitor (SU5402), Mkk6-EE mRNA (+OGDBs) microinjected									
	TOTAL NUMBER OF CELLS								
#	EMBRYO	TE	Nanog -ve TE cells	Nanog +ve TE cells	ICM				
					Nanog	Gata4	Nanog/Gata4 (co-expressed)	Nanog/Gata4 -ve	TOTAL
1	70	60	60	0	0	10	0	0	10
2	69	58	56	2	3	8	0	0	11
3	61	52	52	0	0	9	0	0	9
4	79	74	74	0	1	4	0	0	5
5	42	22	14	8	16	2	1	1	20
6	75	65	65	0	0	10	0	0	10
7	42	33	29	4	4	2	3	0	9
8	66	56	51	5	4	4	0	2	10
9	81	57	56	1	11	11	1	1	24
10	61	52	50	2	4	4	1	0	9
11	74	60	60	0	7	7	0	0	14
12	72	62	62	0	4	4	1	1	10
13	60	50	46	4	6	4	0	0	10
14	71	62	59	3	0	9	0	0	9
15	79	65	65	0	8	5	0	1	14
16	53	46	43	3	2	5	0	0	7
17	41	35	35	0	3	3	0	0	6
18	91	74	74	0	10	6	1	0	17
19	57	50	46	4	4	3	0	0	7
20	77	54	52	2	6	8	1	8	23
21	65	51	51	0	7	7	0	0	14
22	33	27	24	3	3	0	2	1	6
23	55	48	33	15	5	2	0	0	7
24									
**TOTAL**	1474	1213	1157	56	108	127	11	15	261
**AVERAGE**	**64.1**	**52.7**	**50.3**	**2.4**	**4.7**	**5.5**	**0.5**	**0.7**	**11.3**
**SEM**	3.1	2.8	3.2	0.7	0.8	0.6	0.2	0.4	1.1
Stat. sig. (exp. vs. con. embryos; ST11a) *p<0.05, **p<0.005	**	*		**	**	*	*		**
p-value (2-tailed students t-test)	3.31E-05	3.09E-02	6.04E-01	9.60E-07	6.31E-08	1.94E-02	3.10E-02	5.64E-01	1.15E-08
Stat. sig. (exp. vs. exp embryos; ST11b) §p<0.05, §§p<0.005			§	§§	§§	§§			§§
p-value (2-tailed students t-test)	9.92E-02	7.54E-01	2.27E-02	2.89E-09	7.50E-10	5.28E-05	2.56E-01	7.61E-01	5.18E-05
Stat. sig. (exp. vs. exp embryos; ST11c) †p<0.05, ††p<0.005					†	††			
p-value (2-tailed students t-test)	3.52E-01	2.74E-01	3.90E-01	6.18E-01	2.75E-02	2.58E-03	1.69E-01	2.20E-01	7.85E-01

Supplementary table ST11d

+Fgf-receptor inhibitor (SU5402) + p38-Mapk14/11 (SB22025) inhibitors, Mkk6-EE mRNA (+OGDB) microinjected									
	TOTAL NUMBER OF CELLS								
					ICM				
#	EMBRYO	TE	Nanog -ve TE cells	Nanog +ve TE cells	Nanog	Gata4	Nanog/Gata4 (co-expressed)	Nanog/Gata4 -ve	TOTAL
1	52	40	27	13	9	2	0	1	12
2	42	32	9	23	10	0	0	0	10
3	57	40	30	10	15	2	0	0	17
4	72	59	22	37	11	0	0	2	13
5	59	50	25	25	6	3	0	0	9
6	52	40	31	9	12	0	0	0	12
7	39	30	12	18	8	1	0	0	9
8	59	43	15	28	12	2	0	2	16
9	38	20	8	12	15	2	0	1	18
10	38	27	20	7	11	0	0	0	11
11	48	36	28	8	2	8	0	2	12
12	68	46	28	18	19	0	0	3	22
13	44	30	15	15	11	0	0	3	14
14	61	48	24	24	12	0	0	1	13
15	58	47	31	16	11	0	0	0	11
16	47	30	12	18	15	0	0	2	17
17	43	27	18	9	15	1	0	0	16
18	28	21	12	9	6	0	0	1	7
19	54	36	26	10	11	5	0	2	18
20	42	26	9	17	15	0	0	1	16
21	55	29	11	18	23	0	0	3	26
22	50	43	38	5	6	1	0	0	7
23									
24									
**TOTAL**	1106	800	451	349	255	27	0	24	306
**AVERAGE**	**50.3**	**36.4**	**20.5**	**15.9**	**11.6**	**1.2**	**0.0**	**1.1**	**13.9**
**SEM**	2.3	2.2	1.9	1.7	1.0	0.4	0.0	0.2	1.0
Stat. sig. (exp. vs. con. embryos; ST11a) *p<0.05, **p<0.005	**	**	**	**		**	**	*	**
p-value (2-tailed students t-test)	8.40E-12	6.46E-09	9.18E-13	6.50E-04	5.43E-01	8.55E-12	1.62E-04	1.66E-02	1.91E-06
Stat. sig. (exp. vs. exp embryos; ST11b) §p<0.05, §§p<0.005	§§	§§	§§	§	§§		§§		§§
p-value (2-tailed students t-test)	7.51E-07	2.28E-05	1.03E-06	5.59E-02	4.67E-03	3.36E-01	1.62E-04	2.76E-01	2.21E-03
Stat. sig. (exp. vs. exp embryos; ST11c) †p<0.05, ††p<0.005	††	††	††	††	††	††		††	†
p-value (2-tailed students t-test)	5.31E-06	1.16E-07	4.33E-13	2.54E-09	2.52E-10	6.68E-13	6.16E-02	7.24E-04	1.76E-02
Stat. sig. (exp. vs. exp embryos; ST11d) #p<0.05, ##p<0.005	##	##	##	##	##	##	#		
p-value (2-tailed students t-test)	8.37E-04	3.53E-05	5.08E-10	2.92E-09	2.89E-06	1.55E-06	6.92E-03	3.12E-01	9.30E-02

Supplementary table ST11e

Supplementary tables ST11 (a - e): Individual embryo data used to generate averaged data presented in figure 4 (Fgfr inhibition and Mkk6-EE rescue/ p38-Mapk14/11 inhibition ablation); *the average number of cells contributing to all blastocyst cell lineages in embryos microinjected in both blastomeres at the 2-cell (E1.5) stage with either control GFP or constitutively active Mkk6-EE mutant mRNA (plus Oregon-green conjugated dextran beads/ OGDBs; injection marker), in vitro cultured to the 16-cell (E3.0) stage and transferred into growth media supplemented with either Fgfr inhibitor (+SU5402) or vehicle control (+DMSO) before being further cultured until the late-blastocyst (E4.5) stage and immuno-fluorescently stained for Nanog and Gata4. Note an additional group of Mkk6-EE microinjected embryos that had been transferred to Fgfr specific inhibitor, were further transferred into media supplemented with both Fgfr and p38-Mapk14/11 inhibitor (+SU5402 + SB220025) at the early-blastocyst (E3.5) stage before continued culture to the late-blastocyst (E4.5) cell stage and identical immuno-fluorescent staining as the other described groups.*

Supplementary tables ST12 (a-e)

+DMSO, GFP mRNA (+OGDB) microinjected									
	TOTAL NUMBER OF CELLS								
					ICM				
#	EMBRYO	TE	Nanog -ve TE cells	Nanog +ve TE cells	Nanog	Gata4	Nanog/Gata4 (co-expressed)	Nanog/Gata4 -ve	TOTAL
1	85	64	46	18	11	8	2	0	21
2	49	34	29	5	8	5	1	1	15
3	90	70	57	13	9	11	0	0	20
4	78	56	53	3	15	6	0	1	22
5	79	57	34	23	12	6	3	1	22
6	72	56	45	11	6	10	0	0	16
7	69	49	39	10	11	5	3	1	20
8	64	51	45	6	5	7	1	0	13
9	74	60	55	5	4	9	1	0	14
10	66	52	42	10	8	3	2	1	14
11	79	61	57	4	8	9	1	0	18
12	87	61	55	6	15	7	4	0	26
13	102	79	77	2	12	11	0	0	23
14	100	74	71	3	11	13	1	1	26
15	94	73	70	3	13	7	0	1	21
16	100	78	77	1	8	14	0	0	22
17	79	50	45	5	17	8	3	1	29
18	95	75	73	2	7	9	4	0	20
19	83	58	56	2	18	6	1	0	25
20	63	46	40	6	8	8	1	0	17
21	90	60	53	7	16	10	3	1	30
22	105	81	78	3	10	14	0	0	24
23									
24									
25									
**TOTAL**	1803	1345	1197	148	232	186	31	9	458
**AVERAGE**	**82.0**	**61.1**	**54.4**	**6.7**	**10.5**	**8.5**	**1.4**	**0.4**	**20.8**
**SEM**	3.1	2.6	3.1	1.2	0.8	0.6	0.3	0.1	1.0

Supplementary table ST12a

+Tak1 inhibitor (5Z-7-Oxo), GFP mRNA (+OGDB) microinjected									
#	TOTAL NUMBER OF CELLS								
					ICM				
	EMBRYO	TE	Nanog -ve TE cells	Nanog +ve TE cells	Nanog	Gata4	Nanog/Gata4 (co-expressed)	Nanog/Gata4 -ve	TOTAL
1	70	54	44	10	10	3	3	0	16
2	54	36	35	1	13	1	4	0	18
3	70	50	40	10	14	3	2	1	20
4	62	45	28	17	14	0	1	2	17
5	87	71	57	14	9	4	3	0	16
6	67	51	47	4	12	1	1	2	16
7	64	51	39	12	8	4	0	1	13
8	73	59	57	2	6	6	2	0	14
9	85	56	49	7	21	5	3	0	29
10	63	47	43	4	11	1	3	1	16
11	86	64	60	4	13	8	1	0	22
12	87	58	52	6	18	7	3	1	29
13	84	64	58	6	14	6	0	0	20
14	84	69	64	5	12	2	0	1	15
15	76	68	50	18	8	0	0	0	8
16	81	61	53	8	18	2	0	0	20
17	76	53	46	7	18	3	2	0	23
18	92	67	63	4	17	3	4	1	25
19	79	63	55	8	13	2	0	1	16
20	90	70	67	3	12	7	1	0	20
21	70	54	50	4	9	4	3	0	16
22	42	34	26	8	5	3	0	0	8
23	76	60	53	7	13	2	1	0	16
24									
25									
**TOTAL**	1718	1305	1136	169	288	77	37	11	413
**AVERAGE**	**74.7**	**56.7**	**49.4**	**7.3**	**12.5**	**3.3**	**1.6**	**0.5**	**18.0**
**SEM**	2.6	2.1	2.3	0.9	0.8	0.5	0.3	0.1	1.1
Stat. sig. (exp. vs. con. embryos; ST12a) *p<0.05, **p<0.005						**			
p-value (2-tailed students t-test)	7.78E-02	1.93E-01	1.95E-01	6.81E-01	1.04E-01	5.53E-08	6.32E-01	6.97E-01	6.50E-02

Supplementary table ST12b

+DMSO, Mkk6-EE mRNA (+OGDB) microinjected									
	TOTAL NUMBER OF CELLS								
					ICM				
#	EMBRYO	TE	Nanog -ve TE cells	Nanog +ve TE cells	Nanog	Gata4	Nanog/Gata4 (co-expressed)	Nanog/Gata4 -ve	TOTAL
1	72	54	54	0	9	8	1	0	18
2	47	36	29	7	3	6	2	0	11
3	64	51	48	3	3	8	0	2	13
4	67	50	50	0	9	8	0	0	17
5	51	42	36	6	3	5	0	1	9
6	54	43	41	2	6	4	1	0	11
7	48	45	44	1	1	2	0	0	3
8	59	53	50	3	1	5	0	0	6
9	77	57	55	2	4	16	0	0	20
10	39	32	32	0	0	7	0	0	7
11	56	45	32	13	5	3	1	2	11
12	41	31	16	15	0	6	4	0	10
13	97	84	84	0	3	10	0	0	13
14	62	49	47	2	4	9	0	0	13
15	57	43	30	13	4	7	3	0	14
16	81	64	64	0	7	10	0	0	17
17	81	70	70	0	0	11	0	0	11
18	68	51	45	6	12	4	0	1	17
19	74	66	62	4	2	6	0	0	8
20	78	66	66	0	1	11	0	0	12
21	51	40	38	2	0	9	2	0	11
22	76	67	67	0	3	6	0	0	9
23	55	44	31	13	4	7	0	0	11
24	46	24	9	15	6	13	2	1	22
25	83	66	66	0	2	15	0	0	17
TOTAL	1584	1273	1166	107	92	196	16	7	311
AVERAGE	63.4	50.9	46.6	4.3	3.7	7.8	0.6	0.3	12.4
SEM	3.0	2.8	3.6	1.1	0.6	0.7	0.2	0.1	0.9
Stat. sig. (exp. vs. con. embryos; ST12a) *p<0.05, **p<0.005	**	*			**		*		**
p-value (2-tailed students t-test)	9.13E-05	1.12E-02	1.12E-01	1.29E-01	2.84E-08	5.21E-01	3.92E-02	4.38E-01	1.64E-07

Supplementary table ST12c

+Tak1 inhibitor (5Z-7-Oxo), Mkk6-EE mRNA (+OGDBs) microinjected									
	TOTAL NUMBER OF CELLS								
					ICM				
#	EMBRYO	TE	Nanog -ve TE cells	Nanog +ve TE cells	Nanog	Gata4	Nanog/Gata4 (co-expressed)	Nanog/Gata4 -ve	TOTAL
1	56	42	41	1	4	9	1	0	14
2	56	45	37	8	4	5	2	0	11
3	74	56	53	3	7	7	3	1	18
4	72	63	63	0	2	7	0	0	9
5	50	38	31	7	9	2	0	1	12
6	64	51	45	6	10	1	1	1	13
7	63	54	53	1	3	6	0	0	9
8	59	45	40	5	5	3	4	2	14
9	61	51	49	2	6	4	0	0	10
10	54	50	49	1	1	3	0	0	4
11	54	40	38	2	9	4	1	0	14
12	79	65	64	1	7	7	0	0	14
13	75	49	49	0	14	9	2	1	26
14	87	75	71	4	4	8	0	0	12
15	66	47	36	11	9	2	5	3	19
16	79	72	72	0	0	7	0	0	7
17	87	70	69	1	7	10	0	0	17
18	61	47	43	4	6	6	0	2	14
19	107	86	79	7	6	15	0	0	21
20	87	66	63	3	13	7	1	0	21
21	76	60	49	11	11	3	1	1	16
22	64	45	37	8	16	3	0	0	19
23	71	53	52	1	8	7	3	0	18
24	90	73	69	4	6	9	2	0	17
25	83	66	64	2	4	13	0	0	17
**TOTAL**	1775	1409	1316	93	171	157	26	12	366
**AVERAGE**	**71.0**	**56.4**	**52.6**	**3.7**	**6.8**	**6.3**	**1.0**	**0.5**	**14.6**
**SEM**	2.8	2.5	2.7	0.7	0.8	0.7	0.3	0.2	1.0
Stat. sig. (exp. vs. con. embryos; ST12a) *p<0.05, **p<0.005	*			*	**	*			**
p-value (2-tailed students t-test)	1.20E-02	1.93E-01	6.68E-01	2.70E-02	2.31E-03	2.49E-02	3.72E-01	7.28E-01	7.36E-05
Stat. sig. (exp. vs. exp embryos; ST12b) *p<0.05, **p<0.005				§§	§§	§§			§
p-value (2-tailed students t-test)	3.40E-01	9.09E-01	3.65E-01	2.49E-03	1.18E-05	1.18E-03	1.72E-01	9.94E-01	2.98E-02
Stat. sig. (exp. vs. exp embryos; ST12c) †p<0.05, ††p<0.005					††				
p-value (2-tailed students t-test)	6.93E-02	1.56E-01	1.86E-01	6.56E-01	2.87E-03	1.19E-01	2.75E-01	3.35E-01	1.05E-01

Supplementary table ST12d

+Tak1 (5Z-7-Oxo) + p38-Mapk14/11 inhibitors, Mkk6-EE mRNA (+OGDBs) microinjected									
	TOTAL NUMBER OF CELLS								
					ICM				
#	EMBRYO	TE	Nanog -ve TE cells	Nanog +ve TE cells	Nanog	Gata4	Nanog/Gata4 (co-expressed)	Nanog/Gata4 -ve	TOTAL
1	84	58	21	37	21	2	1	2	26
2	29	25	16	9	2	2	0	0	4
3	72	52	27	25	18	0	0	2	20
4	51	46	32	14	5	0	0	0	5
5	73	58	24	34	15	0	0	0	15
6	68	48	24	24	17	0	2	1	20
7	61	45	13	32	12	2	2	0	16
8	65	43	40	3	18	4	0	0	22
9	56	41	27	14	14	0	0	1	15
10	63	49	39	10	12	0	0	2	14
11	64	46	25	21	15	1	0	2	18
12	73	41	24	17	32	0	0	0	32
13	56	48	36	12	8	0	0	0	8
14	70	37	22	15	29	0	0	4	33
15	47	38	27	11	9	0	0	0	9
16	42	30	26	4	10	0	0	2	12
17	62	32	15	17	29	0	0	1	30
18	52	36	23	13	11	3	1	1	16
19	68	58	38	20	7	2	0	1	10
20	60	41	20	21	16	0	2	1	19
21	55	37	13	24	18	0	0	0	18
22									
23									
24									
25									
**TOTAL**	1271	909	532	377	318	16	8	20	362
**AVERAGE**	**60.5**	**43.3**	**25.3**	**18.0**	**15.1**	**0.8**	**0.4**	**1.0**	**17.2**
**SEM**	2.7	2.0	1.8	2.0	1.7	0.3	0.2	0.2	1.8
Stat. sig. (exp. vs. con. embryos; ST12a) *p<0.05, **p<0.005	**	**	**	**	*	**	**	*	
p-value (2-tailed students t-test)	5.67E-06	2.60E-06	5.78E-10	1.59E-05	1.85E-02	5.30E-14	4.07E-03	3.80E-02	8.46E-02
Stat. sig. (exp. vs. exp embryos; ST12b) §p<0.05, §§p<0.005	§§	§§	§§	§§		§§	§§		
p-value (2-tailed students t-test)	4.30E-04	3.53E-05	2.10E-10	1.25E-05	1.66E-01	3.37E-05	9.02E-04	8.22E-02	7.29E-01
Stat. sig. (exp. vs. exp embryos; ST12c) †p<0.05, ††p<0.005		†	††	††		††		†	†
p-value (2-tailed students t-test)	4.91E-01	3.81E-02	8.14E-06	1.12E-07	2.92E-08	2.99E-11	3.68E-01	1.08E-02	1.54E-02
Stat. sig. (exp. vs. exp embryos; ST12d) #p<0.05, ##p<0.005	#	##	##	##	##	##			
p-value (2-tailed students t-test)	1.06E-02	2.57E-04	2.60E-10	5.14E-09	3.02E-05	1.18E-08	6.29E-02	9.79E-02	1.89E-01

Supplementary table ST12e

Supplementary tables ST12 (a - e): Individual embryo data used to generate averaged data presented in figure 5 (Tak1 inhibition and Mkk6-EE rescue/ p38-Mapk14/11 inhibition ablation); *the average number of cells contributing to all blastocyst cell lineages in embryos microinjected in both blastomeres at the 2-cell (E1.5) stage with either control GFP or constitutively active Mkk6-EE mutant mRNA (plus Oregon-green conjugated dextran beads/ OGDBs; injection marker), in vitro cultured to the 8-cell (E2.5) stage and transferred into growth media supplemented with either Tak1 inhibitor (+5Z-7-Oxo) or vehicle control (+DMSO) before being further cultured until the late-blastocyst (E4.5) stage and immuno-fluorescently stained for Nanog and Gata4. Note an additional group of Mkk6-EE microinjected embryos that had been transferred to Tak1 specific inhibitor, were further transferred into media supplemented with both Tak1 and p38-Mapk14/11 inhibitor (+5Z-7-Oxo + SB220025) at the early-blastocyst (E3.5) stage before continued culture to the late-blastocyst (E4.5) cell stage and identical immuno-fluorescent staining as the other described groups.*

Supplementary tables ST13 (a-d)

DMSO (5µM group, IF: Nanog/ Gata4)									
	TOTAL NUMBER OF CELLS								
					ICM				
#	EMBRYO	TE	Nanog -ve TE cells	Nanog +ve TE cells	Nanog	Gata4	Nanog/Gata4 (co-expressed)	Nanog/Gata4 -ve	TOTAL
1	90	65	54	11	14	6	4	1	25
2	88	72	45	27	7	9	0	0	16
3	83	68	56	12	6	7	1	1	15
4	72	52	46	6	11	8	1	0	20
5	84	58	48	10	15	9	0	2	26
6	85	63	53	10	10	11	1	0	22
7	102	78	66	12	13	10	1	0	24
8	87	66	34	32	11	8	2	0	21
9	99	77	58	19	12	8	2	0	22
10	64	51	49	2	8	4	1	0	13
11	88	57	49	8	19	11	1	0	31
12									
13									
TOTAL	942	707	558	149	126	91	14	4	235
**AVERAGE**	**85.6**	**64.3**	**50.7**	**13.5**	**11.5**	**8.3**	**1.3**	**0.4**	**21.4**
SEM	3.2	2.8	2.5	2.7	1.1	0.6	0.3	0.2	1.6

Supplementary table ST13a

p38-Mapk α/β inhibitor (5μM SB220025, IF: Nanog/ Gata4)									
	TOTAL NUMBER OF CELLS								
#					ICM				
	EMBRYO	TE	Nanog -ve TE cells	Nanog +ve TE cells	Nanog	Gata4	Nanog/Gata4 (co-expressed)	Nanog/Gata4 -ve	TOTAL
1	68	53	29	24	14	0	0	1	15
2	81	61	49	12	9	4	6	1	20
3	74	57	38	19	14	1	2	0	17
4	96	72	46	26	12	9	2	1	24
5	87	62	42	20	15	7	2	1	25
6	89	70	51	19	14	3	1	1	19
7	90	59	46	13	26	3	1	1	31
8	92	57	49	8	20	9	6	0	35
9	72	58	48	10	11	2	1	0	14
10	87	59	31	28	24	2	2	0	28
11	61	47	42	5	13	1	0	0	14
12	107	69	40	29	31	4	3	0	38
13	85	59	48	11	19	4	3	0	26
**TOTAL**	1089	783	559	224	222	49	29	6	306
**AVERAGE**	**83.8**	**60.2**	**43.0**	**17.2**	**17.1**	**3.8**	**2.2**	**0.5**	**23.5**
**SEM**	3.4	1.9	1.9	2.2	1.8	0.8	0.5	0.1	2.2
Stat. sig. (exp. vs. con. embryos; ST13a) *p<0.05, **p<0.005			*		*	**			
p-value (2-tailed students t-test)	7.00E-01	2.32E-01	2.06E-02	2.99E-01	1.96E-02	3.20E-04	1.59E-01	6.92E-01	4.46E-01

Supplementary table ST13b

DMSO (10μM group, IF: Nanog/ Gata4)

#	TOTAL NUMBER OF CELLS								
	EMBRYO	TE	Nanog -ve TE cells	Nanog +ve TE cells	ICM				
					Nanog	Gata4	Nanog/Gata4 (co-expressed)	Nanog/Gata4 -ve	TOTAL
1	75	60	53	7	10	5	0	0	15
2	65	48	31	17	10	6	1	0	17
3	96	76	63	13	9	8	3	0	20
4	73	54	49	5	11	7	1	0	19
5	82	62	58	4	10	9	1	0	20
6	92	76	67	9	6	8	2	0	16
7	91	71	66	5	10	9	1	0	20
8	84	71	67	4	6	6	1	0	13
9	72	51	47	4	12	7	2	0	21
10	90	68	68	0	12	8	2	0	22
11	76	57	51	6	9	9	1	0	19
12	86	67	52	15	9	8	2	0	19
13									
TOTAL	982	761	672	89	114	90	17	0	221
**AVERAGE**	**81.8**	**63.4**	**56.0**	**7.4**	**9.5**	**7.5**	**1.4**	**0.0**	**18.4**
SEM	2.8	2.8	3.2	1.5	0.6	0.4	0.2	0.0	0.8
Stat. sig. (exp. vs. con. embryos; ST13a) *p<0.05, **p<0.005									
p-value (2-tailed students t-test)	3.79E-01	8.29E-01	2.12E-01	5.49E-02	1.28E-01	2.98E-01	7.21E-01	7.51E-02	1.00E-01

Supplementary table ST13c

p38-Mapk α/β inhibitor (10μM SB220025, IF: Nanog/ Gata4)									
	TOTAL NUMBER OF CELLS								
#	EMBRYO	TE	Nanog -ve TE cells	Nanog +ve TE cells	ICM				
					Nanog	Gata4	Nanog/Gata4 (co-expressed)	Nanog/Gata4 -ve	TOTAL
1	73	53	19	34	16	4	0	0	20
2	84	60	44	16	18	5	1	0	24
3	85	60	34	26	19	4	1	1	25
4	67	52	28	24	14	0	0	1	15
5	70	49	34	15	12	7	0	2	21
6	84	57	45	12	20	5	1	1	27
7	89	74	41	33	8	6	1	0	15
8	57	31	24	7	26	0	0	0	26
9	83	59	47	12	14	8	2	0	24
10	66	43	29	14	18	4	1	0	23
11	75	48	19	29	22	3	2	0	27
12	81	65	40	25	15	0	0	1	16
13	90	63	42	21	19	7	0	1	27
**TOTAL**	1004	714	446	268	221	53	9	7	290
**AVERAGE**	**77.2**	**54.9**	**34.3**	**20.6**	**17.0**	**4.1**	**0.7**	**0.5**	**22.3**
**SEM**	2.8	3.0	2.7	2.4	1.3	0.8	0.2	0.2	1.3
Stat. sig. (exp. vs. con. embryos; ST13c) *p<0.05, **p<0.005		*	**	**	**	**	*	**	*
p-value (2-tailed students t-test)	2.55E-01	4.98E-02	2.81E-05	1.25E-04	2.63E-05	6.41E-04	2.80E-02	9.70E-03	1.63E-02
Stat. sig. (exp. vs. exp. embryos; ST13b) §p<0.05, §§p<0.005			§				§		
p-value (2-tailed students t-test)	1.54E-01	1.50E-01	1.53E-02	3.09E-01	9.73E-01	7.83E-01	1.28E-02	7.44E-01	6.32E-01

Supplementary table ST13d

Supplementary tables ST13 (a – d): Individual embryo data used to generate averaged data presented in supplementary figure S14; *the average number of cells contributing to all blastocyst cell lineages in embryos in vitro cultured in the presence of 5μm or 10μm of p38-Mapk14/11 inhibiting drug SB220025 or vehicle control DMSO (at a concentration appropriate/ relevant to the two SB220025 concentrations employed), from the early (E3.5) to late blastocyst (E4.5) stages in embryos immuno-fluorescently stained for Nanog and Gata4.*

Supplementary tables ST14 (a-d)

+DMSO, GFP mRNA (+OGDB) microinjected									
	TOTAL NUMBER OF CELLS								
					ICM				
#	EMBRYO	TE	Nanog -ve TE cells	Nanog +ve TE cells	Nanog	Gata4	Nanog/Gata4 (co-expressed)	Nanog/Gata4 -ve	TOTAL
1	107	84	66	18	13	7	3	0	23
2	82	56	54	2	17	6	1	2	26
3	45	25	25	0	7	7	3	3	20
4	73	58	58	0	7	7	0	1	15
5	57	48	47	1	5	4	0	0	9
6	82	64	57	7	12	4	2	0	18
7	94	77	63	14	10	7	0	0	17
8	93	69	69	0	10	13	1	0	24
9	56	40	40	0	9	6	1	0	16
10	59	43	34	9	8	5	3	0	16
11	101	84	71	13	7	9	0	1	17
12	97	73	60	13	15	6	1	2	24
13	76	57	50	7	12	7	0	0	19
14	98	79	71	8	7	12	0	0	19
15	86	70	65	5	8	7	0	1	16
16	82	65	64	1	6	10	0	1	17
17	65	53	51	2	7	3	2	0	12
18	83	65	61	4	7	11	0	0	18
19	75	54	50	4	13	7	1	0	21
20	90	68	65	3	10	9	3	0	22
21	84	63	47	16	12	6	3	0	21
22									
23									
**TOTAL**	1685	1295	1168	127	202	153	24	11	390
**AVERAGE**	**80.2**	**61.7**	**55.6**	**6.0**	**9.6**	**7.3**	**1.1**	**0.5**	**18.6**
**SEM**	3.6	3.2	2.7	1.3	0.7	0.6	0.3	0.2	0.9

Supplementary table ST14a

+Mek1/2 inhibitor (PD0325901), GFP mRNA (+OGDB) microinjected									
	TOTAL NUMBER OF CELLS								
#	EMBRYO	TE	Nanog -ve TE cells	Nanog +ve TE cells	ICM				
					Nanog	Gata4	Nanog/Gata4 (co-expressed)	Nanog/Gata4 -ve	TOTAL
§	90	47	46	1	43	0	0	0	43
2	78	54	53	1	24	0	0	0	24
3	71	47	37	10	22	0	0	2	24
4	64	48	42	6	10	4	0	2	16
5	91	55	46	9	35	0	0	1	36
6	61	44	41	3	17	0	0	0	17
7	88	56	52	4	32	0	0	0	32
8	111	58	58	0	51	0	1	1	53
9	78	61	58	3	17	0	0	0	17
10	80	55	55	0	25	0	0	0	25
11	64	34	20	14	30	0	0	0	30
12	68	50	48	2	18	0	0	0	18
13	80	56	51	5	23	0	0	1	24
14	86	62	52	10	22	0	0	2	24
15	70	40	40	0	30	0	0	0	30
16	73	37	32	5	34	0	0	2	36
17	78	51	38	13	27	0	0	0	27
18	75	48	48	0	27	0	0	0	27
19	52	41	29	12	11	0	0	0	11
20	87	62	58	4	23	0	0	2	25
21	66	34	28	6	31	0	0	1	32
22	72	42	36	6	30	0	0	0	30
23	92	60	60	0	29	0	0	3	32
**TOTAL**	1775	1142	1028	114	611	4	1	17	633
**AVERAGE**	**77.2**	**49.7**	**44.7**	**5.0**	**26.6**	**0.2**	**0.0**	**0.7**	**27.5**
**SEM**	2.7	1.8	2.3	0.9	2.0	0.2	0.0	0.2	1.9
Stat. sig. (exp. vs. con. embryos; ST14a) *p<0.05, **p<0.005		**	**		**	**	**		**
p-value (2-tailed students t-test)	4.92E-01	1.96E-03	3.22E-03	4.85E-01	8.55E-10	1.00E-15	1.34E-04	4.43E-01	2.01E-04

Supplementary table ST14b

+DMSO, Mkk6-EE mRNA (+OGDBs) microinjected									
	TOTAL NUMBER OF CELLS								
#	EMBRYO	TE	Nanog -ve TE cells	Nanog +ve TE cells	ICM				
					Nanog	Gata4	Nanog/Gata4 (co-expressed)	Nanog/Gata4 -ve	TOTAL
1	38	30	30	0	2	5	1	0	8
2	50	42	42	0	0	7	1	0	8
3	62	46	45	1	8	8	0	0	16
4	60	53	51	2	0	5	2	0	7
5	55	45	44	1	3	7	0	0	10
6	56	44	44	0	4	8	0	0	12
7	57	45	37	8	4	7	0	1	12
8	65	51	45	6	5	8	1	0	14
9	41	37	37	0	0	4	0	0	4
10	62	51	51	0	3	6	2	0	11
11	58	45	45	0	1	10	0	2	13
12	53	40	40	0	5	8	0	0	13
13	45	28	27	1	4	11	2	0	17
14	48	34	34	0	5	8	1	0	14
15	73	61	61	0	0	12	0	0	12
16	71	56	56	0	2	13	0	0	15
17	55	36	26	10	10	6	2	1	19
18	58	51	51	0	2	5	0	0	7
19	80	70	70	0	0	10	0	0	10
20	61	51	51	0	2	8	0	0	10
21									
22									
23									
TOTAL	1148	916	887	29	60	156	12	4	232
**AVERAGE**	**57.4**	**45.8**	**44.4**	**1.5**	**3.0**	**7.8**	**0.6**	**0.2**	**11.6**
SEM	2.3	2.3	2.5	0.7	0.6	0.5	0.2	0.1	0.8
Stat. sig. (exp. vs. con. embryos; ST14a) *p<0.05, **p<0.005	**	**	**	**	**				**
p-value (2-tailed students t-test)	4.97E-06	3.10E-04	3.74E-03	2.81E-03	1.55E-08	5.16E-01	1.07E-01	1.60E-01	1.40E-06

Supplementary table ST14c

+Mek1/2 inhibitor (PD0325901), Mkk6-EE mRNA (+OGDBs) microinjected

#	TOTAL NUMBER OF CELLS								
	EMBRYO	TE	Nanog -ve TE cells	Nanog +ve TE cells	ICM				
					Nanog	Gata4	Nanog/Gata4 (co-expressed)	Nanog/Gata4 -ve	TOTAL
1	72	54	50	4	9	6	3	0	18
2	99	56	55	1	37	2	2	2	43
3	74	47	43	4	19	5	2	1	27
4	50	36	34	2	11	2	0	1	14
5	75	55	51	4	14	5	1	0	20
6	54	39	37	2	13	0	0	2	15
7	63	43	43	0	13	4	1	2	20
8	56	39	38	1	11	4	2	0	17
9	77	49	44	5	22	2	2	2	28
10	100	72	71	1	19	6	2	1	28
11	71	51	50	1	14	0	4	2	20
12	77	55	49	6	14	5	2	1	22
13	43	34	34	0	9	0	0	0	9
14	55	38	35	3	14	2	0	1	17
15	73	66	65	1	4	2	0	1	7
16	61	45	44	1	11	5	0	0	16
17	74	55	43	12	11	5	3	0	19
18	86	58	53	5	20	6	0	2	28
19	71	49	47	2	5	13	4	0	22
20	84	59	55	4	19	2	2	2	25
21	63	41	40	1	17	2	2	1	22
22	87	64	62	2	20	3	0	0	23
23									
**TOTAL**	1565	1105	1043	62	326	81	32	21	460
**AVERAGE**	**71.1**	**50.2**	**47.4**	**2.8**	**14.8**	**3.7**	**1.5**	**1.0**	**20.9**
**SEM**	3.2	2.2	2.1	0.6	1.5	0.6	0.3	0.2	1.6
Stat. sig. (exp. vs. con. embryos; ST14a) *p<0.05, **p<0.005		*	*	*	**	**			
p-value (2-tailed students t-test)	6.33E-02	5.21E-03	2.04E-02	2.24E-02	3.14E-03	9.77E-05	4.32E-01	1.08E-01	2.16E-01
Stat. sig. (exp. vs. exp. embryos; ST14b) §p<0.05, §§p<0.005					§§	§§	§§		§
p-value (2-tailed students t-test)	1.51E-01	8.41E-01	3.91E-01	5.95E-02	2.17E-05	1.28E-06	9.91E-06	4.30E-01	1.21E-02
Stat. sig. (exp. vs. exp. embryos; ST14c) †p<0.05, ††p<0.005	††				††	††	†	††	††
p-value (2-tailed students t-test)	1.34E-03	1.71E-01	3.53E-01	1.23E-01	1.17E-08	1.17E-05	1.80E-02	1.37E-03	1.18E-05

Supplementary table ST14d

Supplementary tables ST14 (a - d): Individual embryo data used to generate averaged data presented in supplementary figure S15 (Mek1/2 inhibition and Mkk6-EE rescue); *the average number of cells contributing to all blastocyst cell lineages in embryos microinjected in both blastomeres at the 2-cell (E1.5) stage with either control GFP or constitutively active Mkk6-EE mutant mRNA (plus Oregon-green conjugated dextran beads/ OGDBs; injection marker), in vitro cultured to the 8-cell (E2.5) stage and transferred into growth media supplemented with either Mek1/2 inhibitor (+PD0325901) or vehicle control (+DMSO) before being further cultured until the late-blastocyst (E4.5) stage and immuno-fluorescently stained for Nanog and Gata4.*

Supplementary tables ST15 (a&b)

+DMSO E3.5 - E4.5, IF: (Nanog/ Gata6)									
	TOTAL NUMBER OF CELLS								
					ICM				
#	EMBRYO	TE	Nanog -ve TE cells	Nanog +ve TE cells	Nanog	Gata6	Nanog/Gata6 co-expressed)	Nanog/Gata 6 -ve	TOTAL
1	90	63	46	17	8	15	4	0	27
2	99	77	57	20	6	16	0	0	22
3	83	64	48	16	5	7	7	0	19
4	101	77	65	12	5	12	7	0	24
5	81	55	48	7	9	13	4	0	26
6	104	84	70	14	7	11	2	0	20
7	81	63	50	13	4	10	4	0	18
8	75	60	56	4	6	7	2	0	15
9	77	53	50	3	7	9	8	0	24
10	96	75	60	15	7	13	1	0	21
**TOTAL**	887	671	550	121	64	113	39	0	216
**AVERAGE**	**88.7**	**67.1**	**55.0**	**12.1**	**6.4**	**11.3**	**3.9**	**0.0**	**21.6**
**SEM**	3.4	3.3	2.5	1.8	0.5	1.0	0.9	0.0	1.2

Supplementary table ST15a

+Mek1/2 inhibitor (PD0325901) E3.5 - E4.5, (IF: Nanog/ Gata6)									
	TOTAL NUMBER OF CELLS								
					ICM				
#	EMBRYO	TE	Nanog -ve TE cells	Nanog +ve TE cells	Nanog	Gata6	Nanog/Gata6 co-expressed)	Nanog/Gata 6 -ve	TOTAL
1	83	48	46	2	26	0	9	0	35
2	86	63	47	16	15	2	6	0	23
3	109	72	63	9	23	0	14	0	37
4	103	61	48	13	39	0	3	0	42
5	99	62	62	0	24	5	8	0	37
6	103	63	54	9	21	5	14	0	40
7	81	61	58	3	12	3	5	0	20
8	81	63	55	8	14	1	3	0	18
9	92	65	58	7	15	0	12	0	27
10									
**TOTAL**	837	558	491	67	189	16	74	0	279
**AVERAGE**	**93.0**	**62.0**	**54.6**	**7.4**	**21.0**	**1.8**	**8.2**	**0.0**	**31.0**
**SEM**	3.6	2.1	2.1	1.7	2.8	0.7	1.5	0.0	3.0
Stat. sig. (exp vs. con embryos; ST15a) *p<0.05, **p<0.005					**	**	*		*
p-value (2-tailed students t-test)	3.95E-01	2.20E-01	8.96E-01	7.99E-02	4.60E-05	5.64E-07	1.78E-02	1.00E+00	7.92E-03

Supplementary table ST15b

Supplementary tables ST15 (a & b): Individual embryo data used to generate averaged data presented in supplementary figure S16 (Mek1/2 inhibition E3.5 – E4.5, IF: Nanog and Gata6); *the average number of cells contributing to all blastocyst cell lineages in embryos in vitro cultured from the early blastocyst (E3.5) to late blastocyst (E4.5) stages in growth media supplemented with either Mek1/2 inhibitor (+PD0325901) or vehicle control (+DMSO) and immuno-fluorescently stained for Nanog and Gata6.*
